# Far-reaching volcaniclastic density current deposits as evidence of explosive marine eruptions

**DOI:** 10.1038/s41467-026-71658-8

**Published:** 2026-04-30

**Authors:** Jacob A. Nash, Isobel A. Yeo, Michael A. Clare, James Hunt, Andrew B. Cundy, Sally Watson, Richard Wysoczanski, Sarah Seabrook, Kevin Mackay, Shane J. Cronin, Joali Paredes-Mariño, Taaniela Kula, Rennie Vaiomounga, Cían McGuire, Miros S. J. Charidemou

**Affiliations:** 1 Volcanology & Geohazards Laboratory, National Oceanography Centre (NOC), Southampton, UK; 2 School of Ocean and Earth Science, University of Southampton, Southampton, UK; 3 Earth Sciences New Zealand, Auckland, New Zealand; 4 Faculty of Science, University of Auckland, Auckland, New Zealand; 5 Ministry of Lands, Survey, Planning and Natural Resources, Nuku’alofa, Tonga; 6 British Ocean Sediment Core Research Facility (BOSCORF), NOC, Southampton, UK

**Keywords:** Natural hazards, Volcanology, Sedimentology

## Abstract

Underwater sediment density currents triggered by marine volcanic eruptions threaten island communities and infrastructure, while their deposits provide archives of past eruptions. Despite their significance, scarce real-time density current observations and concurrent deposit samples limit our understanding of their behaviour and relationship to varying volcanic mechanisms. Using data acquired following the explosive, VEI 6, shallow-submarine eruption of Hunga Volcano in 2022, we show that syn-eruptive delivery of pyroclastic material into the ocean via low-column collapses and fountaining triggered the multidirectional dispersal of highly-concentrated underwater density currents. Rapid supply of > 6.5 km^3^ of dense pyroclastic material onto the steep volcanic flanks over minutes-to-hours generated currents that maintain high density and velocity 10-100s of kilometres from the volcano. We outline diagnostic criteria to differentiate deposits of shallow-submarine generated underwater currents from other volcanic processes - enabling better reconstruction of the records of volcanic activity in marine sediments and enhancing hazard assessments in submerged volcanic settings worldwide.

## Abstract (web summary)

Volcanic material emplaced onto the seafloor by powerful underwater sediment flows during the largest eruption of the 21st century, Hunga volcano, 2022, enables deposit-based diagnosis of large explosive shallow-submarine eruptions.

## Introduction

Volcaniclastic density currents, powerful seafloor flows that initiate at marine volcanic systems, can transport huge volumes of volcanic material for hundreds of kilometres, modify seafloor topography, and damage critical seafloor infrastructure^1–4^. Despite the risk posed, real-time observations and measurements of such currents remain scarce due to the often-remote or uncharted locations of marine volcanoes^5^ and the logistical challenges in monitoring their eruptions. On the other hand, deposits of such currents are common in the geological record. Thus, correctly interpreting marine volcanic deposits is key to understanding volcanic histories and hazards. Our current understanding of underwater density current initiation, behaviour, and seafloor impacts relies on laboratory-scale modelling^6^ and deposits of ancient events^7,8^. The formation of these currents is attributed to: (1) volcanic eruptions where water mixes with pyroclasts erupted from submarine vents^9^, (2) terrestrially initiated pyroclastic flows that reach the shoreline^10^, or (3) mobilisation of volcaniclastic material during mass-wasting events that may not be related to an eruptive episode (e.g., volcanic flank collapse, oversteepening of the coastal shelf, edifice collapse etc)^11^. Due to the myriad mechanisms of their formation, the subsequent density currents record a broad spectrum of structures, behaviours, and dynamics^2,12^. Here, the term ‘*underwater volcaniclastic density current’* is employed to cover the full-range of density currents produced in marine volcanic settings, from gas-rich and high-temperature pyroclastic density currents, and water-supported and cool volcaniclastic turbidity currents, to debris flows. Coincident direct observations and sampling remain rare, particularly for less frequent large volcanic eruptions; hence, offshore data acquired after the well-observed 2022 shallow-submarine eruption of Hunga Volcano, Kingdom of Tonga, provide an exceptional opportunity to link a major explosive submarine eruption with deposits from resultant powerful density currents^13^.

Here, we provide detailed characterisation of underwater volcaniclastic density current deposits emplaced during the 2022 Hunga eruption. Using repeat seafloor surveys and sediment cores collected two months after the eruption^14,15^, we constrain the behaviour of syn-eruptive, rapidly-dispersed, and multidirectional density currents and appraise how their deposits vary from other density current initiation mechanisms at marine volcanoes. Seafloor volcaniclastic deposits are valuable archives of past volcanic activity; hence, this mechanistic understanding and diagnostic deposit blueprinting of these density currents is critical to aid with the robust reconstruction of the nature and magnitude of hazards posed by different marine volcanoes worldwide.

## Results and discussion

### The 2022 eruption of Hunga Volcano

**Fig. 1:**
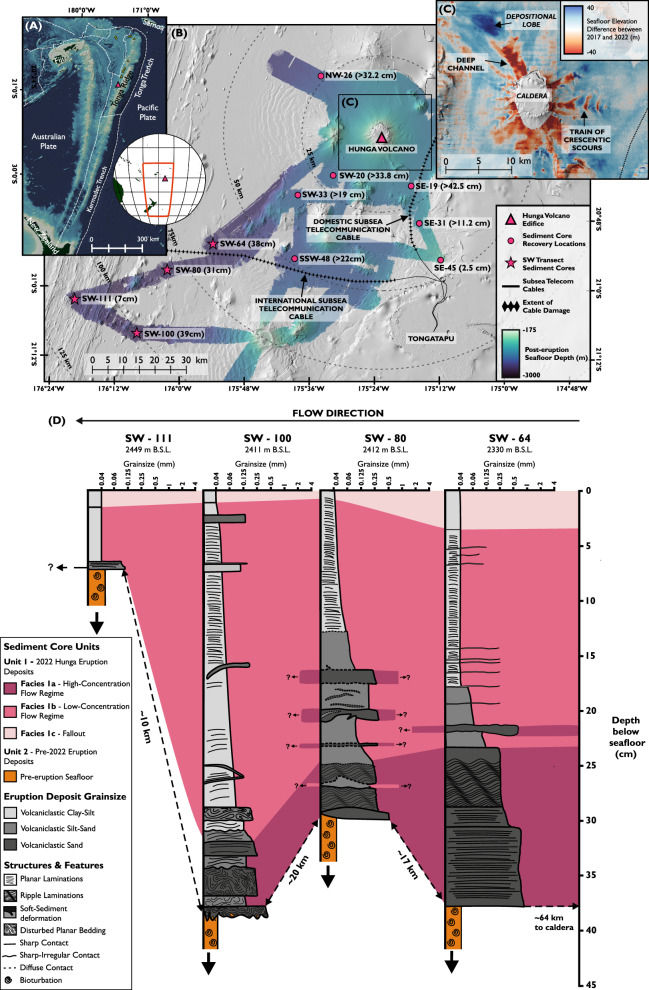
Seafloor impact of underwater volcaniclastic density currents triggered during the January 15th, 2022, Hunga Volcano eruption. **A** Location of Hunga volcano (previously known as Hunga-Tonga Hunga Ha’apai) on the Tonga-Kermadec Arc using the General Bathymetric Chart of the Oceans (GEBCO) 2025 grid^106^ and USGS plate boundary data^107^. **B** Bathymetric data collected 3 months after the eruption on the TESMaP TAN2206 Research cruise with locations of deep-sea telecommunication cable damage and multicoring locations overlain^13–15,102^, all overlaid upon Global Multi-Resolution Topography (GMRT)^105^. Core recovery sites are marked by pink circles. Pink stars mark the location of sediment cores comprising a proximal-to-distal SW transect. Recovered 2022 density current deposit thickness is annotated next to its associated sediment core. Sediment cores where the base of the 2022 deposit was unrecovered, deposit thickness is given as >X cm. **C** Elevation difference map of the Hunga caldera constructed using bathymetric surveys collected before and after the eruption^13–15,102^. Illustrates local radiating excavated channels and trains of crescentic scours down the flanks of Hunga volcano. Lobate deposits are located directly downstream of these channels at distances of approximately 5–6 km from the caldera. **D** Visual sedimentary logs of the sediment cores comprising the SW transect illustrating the evolution of the underwater volcaniclastic density currents as they travelled away from Hunga volcano.

Hunga Volcano is a partially-submerged shallow-submarine caldera on the Tofua Arc (Fig. 1)^16^. The almost entirely submerged volcanic edifice rises from water depths of 1800 m, and prior to the 2022 eruption, recorded a ~ 4 km wide caldera with crater floor depths <200 m below sea level and the rim islands Hunga Tonga and Hunga Ha’apai peaking at 114 m above sea level^16^. Following caldera collapse ~900 years ago volcanism at Hunga had comprised infrequent low-intensity Surtseyan and phreatomagmatic activity^17,18^. On January 15th, 2022, following three weeks of minor Surtseyan volcanic eruptions, activity at Hunga Volcano rapidly escalated into the most explosive volcanic eruption of the 21st century^19,20^. Explosive activity may have been triggered by the intrusion of magma from a deeper reservoir^21^. Erupted glass H_2_O concentrations are consistent with magmatic fragmentation taking places at a subsurface depth of 400–1000 m^22^. Unlike most submarine eruptions, this eruption was extensively documented through satellite monitoring^23^, photography^13^, and remote geophysical networks^24^, providing unparalleled-characterisation. The eruptive phase lasted around 11 h^25^ – with several large explosions between 04:15-04:25 UTC (all times are UTC)^26^, triggering tsunamis^27^ and atmospheric shockwaves that circumnavigated the globe several times^28^. Between 04:15-04:40, increasing mass eruption rate (MER) caused rapid growth of the water-dominated column^22,29,30^ to ~58 km^31^. At ~05:30, caldera collapse formed a ~ 860 m deep caldera^32^. The eruption emplaced at least 6.5 km^3^ of pyroclastic material on the seafloor–largely carried by underwater volcaniclastic density currents. Currents generated early in the eruption achieved speeds of up to 122 km/h, and reshaped the seafloor, smothered benthic communities, and damaged >100 km of seafloor cables, disconnecting Tonga from the internet^13,15^.

### Complex flow regimes revealed from seafloor deposits

Repeat pre- and post-eruption bathymetric surveys and deposits sampled from sediment cores collected two months post-eruption reveal that volcaniclastic sediments were dispersed in multiple directions, radiating from Hunga Volcano for over 100 km (Fig. 1, Supplementary Fig. 1 and Supplementary Table 1). These currents cut deep channels and scours on the upper flanks of the volcano, and deposited <40 m thick lobate deposits at an abrupt slope break 4–5 km from the rim^13^ (Fig. 1). In deeper waters, decimetre-to-centimetre-thick deposits blanket the seafloor with surficial ripple-scale bedforms (Supplementary Fig. 2). To characterise the evolution of these flows in detail, we analyse a transect of four sediment cores (SW-64, SW-80, SW-100, and SW-111) extending up to 111 km southwest of the volcano that record full sequences through the 2022 eruption deposit.

**Fig. 2:**
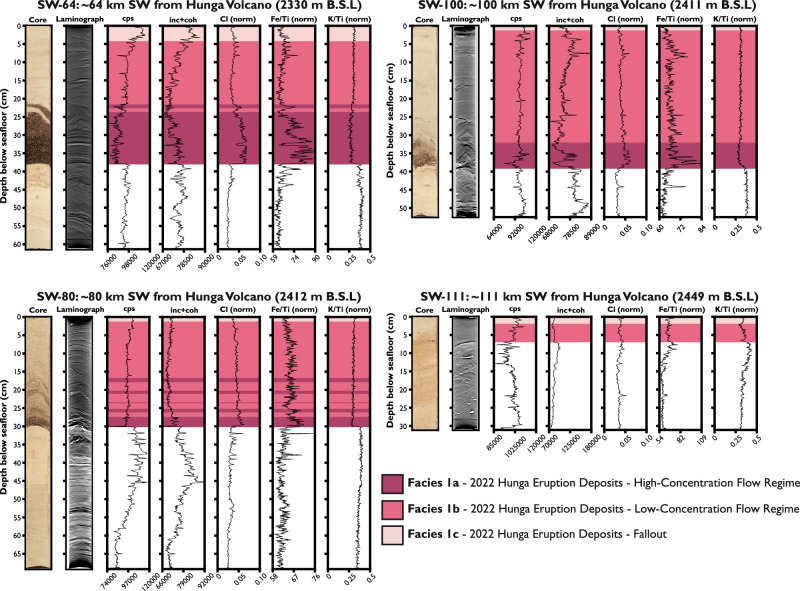
Submarine volcaniclastic density current deposits. A series of panels for each of the sediment cores along the SW transect including brightened core images, laminographs, and downcore Itrax Micro-XRF data enabling identification of the 2022 submarine density current deposits. CPS is counts per second. inc+coh is the incoherent plus coherent scatter. All counts (e.g., Cl) and ratios (e.g., K/Ti) are normalised to inc+coh. Facies 1a, 1b, and 1c are identified – see legend.

**Fig. 3:**
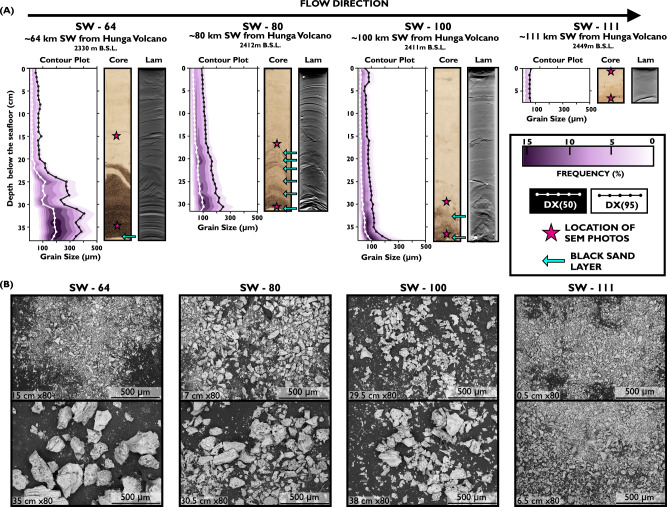
Grainsize analysis of density current deposits along the SW transect. **A** Grainsize distributions as coloured contour plots for the 2022 sediment density currents compared to visual observations from high-resolution core images (IMG) and laminographs (LAM). Dx (50) and Dx (95) lines represent grainsize (μm) that 50% and 95% of the grains are finer at the sampled position in the sediment core, respectively. Circles represent sampling locations. **B** Backscatter scanning electron microscopy (SEM) images of material taken from different depths (pink stars) from the sedimentary cores along the SW transect. Contour plots and SEM images illustrate distinct fining both up-core and moving distally SW away from Hunga. Blue arrows indicate the location of black sand layers indicating the base of a fining upward sequence.

Sediment cores sample two distinct depositional units. The uppermost unit (Unit 1) is interpreted as the 2022 eruption deposits lying unconformably above pre-eruption sediments (Unit 2; Figs. 1 and 2; supplementary information). Unit 1, divided into three Facies 1a, 1b, and 1c, is characterised by stacked fining-upward sequences of volcaniclastic sediments with a sharp basal contact. At the base of Unit 1 is a centimetre-to-decimetre thick layer of black, poorly-to-moderately sorted, sand-silt sized volcaniclastics (Facies 1a). Facies 1a overall displays normal grading, but with some abrupt internal grainsize changes, planar laminations, climbing ripples, and disturbed laminations. Directly above Facies 1a, Facies 1b locally features black sand-silt layers (one to six) of varying thickness (centimetre-to-millimetres) separated by layers of beige silt-clay sized material. The black sand-silt layers are texturally and visually similar to the basal black sand of Facies 1a. Different layers are distinguished by distinct colour (Fig. 2) and grainsize changes (Fig. 3). Beige silt-clay layers are characterised by overall upward-fining, poor-sorting, and millimetre-scale planar laminations. Unit 1 is consistently overlain by Facies 1c, a thin, centimetre (<4 cm), layer of poorly sorted clay, distinguished from material below by a subtle colour change with an absence of internal structures (e.g., the planar laminations of Facies 1b). Facies 1c is not observed/recognised at any other stratigraphic location within the 2022 eruption deposit.

Unit 1 has a distinct geochemical signature, with consistently high Fe/Ti and low K/Ti relative to Unit 2 across all cores (Fig. 2). High iron (Fe) in Unit 1 relates to the high Fe-composition of Hunga glasses^18,22^. Low K/Ti ratios in Unit 1 signify minimal pelagic clay content (e.g., illite; which contains high potassium, K), indicating freshly-emplaced material^33^; further supported by high chlorine (Cl) content, which increases sharply at the contact with Unit 2 (indicating Unit 1 material was sea-water saturated and unconsolidated^34^; Fig. 2). An absence of bioturbation, deposit oxidation, or hemipelagic fallout also support recent emplacement of these deposits^35^.

All facies of Unit 1 are composed of basaltic-andesite to andesitic volcanic glass shards and pumice (85–94%), consistent with compositions of ash sampled immediately following the eruption^15^ (Supplementary Fig. 3, Supplementary Fig. 4, Supplementary Table 2). Unit 1 contains minor components of porphyritic lithics (5–9%), crystal fragments (plagioclase feldspars, pyroxene, and olivine; 1–7%), and biological material (e.g., shell fragments and foraminifera; ~1%). Glasses have variable texture, vesicularity and shape, and include massive glass and microlite-rich forms, bubble wall fragments, and vesicular tubular pumice. Glass shards frequently display step-fractures and curvilinear boundaries characteristic of explosive fragmentation. Microprobe analysis of Unit 1 glasses shows compositional overlap with glasses sampled from 2022 Hunga eruption deposits elswhere^15,22^(Supplementary Fig. 4). Collectively, the geochemical and compositional analysis provide confidence that Unit 1 deposits, which can be correlated between all core sites, are indeed from the 2022 eruption.

Beneath Unit 1 is Unit 2, a compacted, clay-rich but volcanic sourced deposit containing abundant volcanic glass shards. The contact between units is characterised by a pronounced grainsize break and visually distinct colour change. Unit 2 is distinguished from the 2022 eruption deposits by contrasting geochemical signatures (low Fe/Ti, high K/Ti, and low Cl), abundant biogenic material (e.g., shell fragments and foraminifera), alongside intense weathering, oxidation, and bioturbation (Figs. 1 and 2). Instead, these sediments comprised the seafloor prior to the 2022 eruption with a combined source of pelagic fallout and previous volcanic eruptions (from either Hunga or surrounding Kermadec Arc volcanoes).

The combination of normal grading, sharp base, poor-to-moderate sorting, planar and ripple laminations, and volcaniclastic material characterising Unit 1 are diagnostic of emplacement by an underwater volcaniclastic sediment density current; more specifically a volcaniclastic turbidity current^36^. These are sediment gravity currents in which sediment is primarily held in suspension by fluid turbulence, driven to lateral flow by their excess density relative to the surrounding ambient water^37^. The bipartite structure of Unit 1 indicates the parental turbidity current was strongly density stratified with a near-bed high-concentration coarse-grained flow regime dominated by tractional flow and bedload transport (Facies 1a), and a dilute upper layer transporting fine material recorded in the beige planar-laminated muds (Facies 1b)^38–41^. The depositional architecture of Unit 1 is typical of that formed by a high-density turbidity current where turbulent and tractional-bed reworking processes are dampened but not inhibited within a dense sediment-concentrated near bed layer (Facies 1a) and there is a distinct transition into an overlying turbulent dilute flow (Facies 1b), which distinguishes these currents from debris flows^36^. Both Facies 1a and 1b contain disturbed planar laminations (Fig. 1 and Supplementary Fig. 1). The cause of this disturbance is unknown, but can result from syn-depositional shear, rapid sediment loading, fluid escape, or deformation during coring^42–44^.

Moving up-deposit from Facies 1b to 1c, the change in internal deposit structure indicates a shift in depositional mechanism, which has been previously identified as a transition from emplacement by turbulent density currents to eruption plume fallout^45^. Evidence for plume fallout mantling the density current deposits is threefold: all sediment core locations lie within the 2022 eruption plume radius^25^; Facies 1c grainsize and thicknesses (<4 cm) match estimated plume material grainsize (<100 μm) and fallout deposit thicknesses (1–6 cm)^46^; and modelled current velocities indicate density currents reached core sites^13,15^ before plume fallout settled on the seafloor^45^. However, the ungraded veneer of Facies 1c can also be attributed to the uppermost structural division of the turbidite sequence – fallout of fine material suspended in the water-column by a turbulent density current that can remain in suspension for days-to-months^36,47^. This is supported by the occurrence of lithic and biogenic material within Facies 1c that is unlikely to be sourced from plume fallout. Regardless of source, calculated settling rates^45^ and continued settling of suspended particulate documented by video and water-column sampling three months after the eruption^15^, restricts fallout to the top of the deposit. Facies 1c is likely a combination of both plume fallout and settling of material suspended by density currents, that is challenging to conclusively differentiate between them. Despite fallout into the water surrounding Hunga documented during the precursory volcanism at Hunga there is no evidence of a fallout layer between Unit 1 and Unit 2^48^.

### Spatial evolution of underwater density currents

Facies 1a and 1b record sedimentary evidence for deposition from a sediment density current maintaining a high-concentration near-bed layer, with progressive current waning towards the SW (Figs. 2 and 3). Black sand-silt intervals thin and fine distally into centimetre-thick silt-mud layers becoming absent in the most distal core (SW-111). Moving away from the volcanic edifice surface ripples decrease in size before disappearing in the most distal locations (Supplementary Fig. 2). Facies 1a black-sand intervals record a transition from planar to ripple laminations distally and up-core, evidencing contemporaneous bedload transport and high suspended load fallout, diagnostic of turbidity current waning^36^. SW-100 is the most distal location in which the black sand-silt base is identified, indicating maintenance of a high-concentration near-bed flow across at least ~100 km from Hunga Volcano. Beige silt-clay intervals (Facies 1b and 1c) record minimal variation along the transect, showing normal grading, planar lamination, and similar grainsizes, consistent with steady settling of fine material from a dilute suspension^49^ (Fig. 3). Transformation from a high-concentration near-bed to fully turbulent and dilute is characteristic of turbidity current deceleration^50^.

Absence of a black sand-silt base in SW-111 might be due to waning and deposition of the coarsest basal sediment in flow and/or topographic influence. Between Hunga Volcano and the location of SW-111 are a series of high elevation ridges which may have partially impeded the current(s) resulting in segregation of a dilute upper layer that surmounted the ridge without the basal dense component^8^ (Fig. 1). Distal waning and subsequent loss of the ability to maintain coarse and dense particles in suspension may eventually also lead to lofting above the seafloor^51^.

Repeated coarse black sand-silt intervals within Facies 1b consistently thin and fine up-core and along the flow pathways away from Hunga Volcano. Medially (<80 km) contacts between black sand-silt and beige silt-clay layers are sharp and visibly well defined, recording clear grainsize and colour changes, before becoming progressively diffuse with distance from Hunga Volcano (>80 km). These coarser layers reveal repeated reinvigoration of high-concentration near-bed fluxes (Facies 1a) that could be produced by a pulsed turbidity current with temporally-unsteady fluxes, a series of distinct and individually triggered turbidity currents, reflection of flows off surrounding seafloor relief, and/or different travel paths of synchronously-triggered flows^52,53^. A lack of bioturbation or fine-grained muds between these layers indicates a short time interval between their emplacement, while the absence of ripple direction reversal across the cores suggests that black sand-silt repetition is unlikely to be a result of flow reflections. Laboratory experiments show that the depositional signals of distinctly-pulsed turbidity currents or quasi-steady pulsed currents can be obscured or lost as they travel from their source, due to the internal re-organisation of the flow(s) which could explain the amalgamated black sand-silt layers in sediment core SW-100 and the lack of consistency in the absolute number of pulses observed along the transect^52^.

### Rapid entry of voluminous material fuels long-runout currents

Far-reaching volcaniclastic turbidity currents (>100 km) have been attributed to volcanic flank collapse^54^ and water-entering pyroclastic density currents^55^. Yet, there is no sedimentological or bathymetric evidence for either of these processes occurring during the 2022 Hunga eruption. Instead, we observe: (1) caldera collapse^32^; (2) erosive bedforms on the upper volcanic flanks; (3) large-scale deposition (<40 m) following the first slope break; (4) multidirectional deposition of centimetre-to-decimetre-thick volcaniclastic turbidites >111 km from Hunga Volcano.

**Fig. 4:**
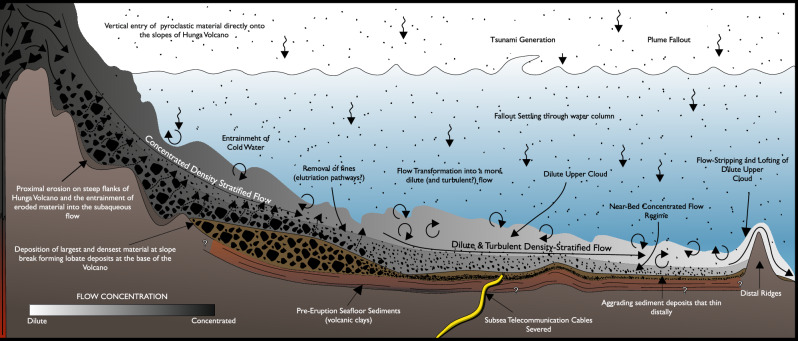
Far-reaching underwater volcaniclastic density currents. Model of underwater volcaniclastic density current structure, behaviour, and evolution, during the January 15th, 2022, Hunga volcano eruption.

Despite the evacuation of ~6.5 km^3^ of magma (Dense Rock Equivalent, DRE) during the eruption, the 58 km high eruption column was tephra poor (<0.1–0.2 km^3^ of erupted volume)^22,29,30^. Instead, the bulk of erupted material entered the ocean^22,46^. We therefore attribute underwater volcaniclastic density current formation to syn-eruptive and vertical delivery of large volumes of erupted material into the ocean via plume collapse^13,15^ and submarine or low-level subaerial fountaining and jetting leading to lateral density current dispersal (Fig. 4)^22,56^. Direct observations of low column-collapses and tsunami onset timings indicate the continued delivery/supply of pyroclastic material into the ocean and the semi-continuous formation of underwater currents during the period of highest MER (timeframe of most rapid column growth between ~04:00 and 05:10) shortly after eruption onset^13,57,58^, supported by unsteady and pulsatory seismic energy records of the eruption^24^. Semi-continuous generation of underwater volcaniclastic density currents, over a short time period supports our interpretation that the repeated fining upward nature of the 2022 deposits is indicative of deposition from a series of quasi-steady pulsed currents. The close timing between the first major explosion (and rapid column growth), visible plume collapses, and cable damage, support low-column collapses as the primary mechanism of initial current formation^13^. However, the precise initiation mechanism(s) (i.e. column-collapse, fountaining, jetting, or combinations thereof typically associated with a large explosive eruption), are all capable of, and likely all contribute to, rapidly and vertically delivering large volumes of dense material into the ocean directly onto submerged volcano flanks, providing an efficient mechanism to trigger pulses of density currents in multiple directions^13^.

Lacking directly comparable field-studies to the 2022 Hunga eruption, we draw on case-studies of eruptions and density-current behaviours that provide valuable analogues for interpreting the 2022 Hunga submarine deposits and density current behaviours. Close to the volcano, density currents may have been gas-supported, high-temperature, high-density flows, before transitioning at some distance or depth into water-supported turbidity currents^6,8,10,59^. Underwater volcaniclastic density currents triggered by lake/ocean entry of hot pyroclastic density currents record deposition of their densest and coarsest material proximally (or in shallow waters) as fines-depleted breccias or lobate deposits, causing elutriation of fine and low-density material, which mixes with ambient water to form a cool and relatively low-concentration turbidity current^8,10^. Cores >20 km from Hunga record no sedimentological evidence of gas-supported flow or high-temperature (>650 °C) emplacement (e.g., welding); hence we infer that at least 20 km from Hunga Volcano the currents were cool, water-supported, turbidity currents (Supplementary Fig. 1). Thick (<40 m) depositional lobes emplaced at the first major slope break away from the volcanic edifice of Hunga (Fig. 1), and coarse/competent deposits that could not be cored close to (<20 km) Hunga^14^, may record similar sudden emplacement of the coarsest/densest material from the initial currents. These lobes potentially mark the location of flow transformation into water-supported turbidity currents, but our data do not allow identification of the transition from hot to cold density currents.

Following flow transformation, turbidity currents dispersed in all directions away from Hunga for over 100 km (Fig. 1). Multidirectional flow of underwater volcaniclastic density currents is a recorded feature of column collapse at other shallow-submarine volcanoes e.g., Santorini^60^. Additionally, terrestrial eruptions of equivalent magnitude to the Hunga 2022 eruption, e.g., 1991 eruption of Mount Pinatubo (3.7–5.4 km^3^ of erupted magma) record column-collapse triggered pyroclastic density currents dispersing radially away from the volcanic edifice^61^. Pre- and post-eruption bathymetry show that the volcaniclastic material around Hunga Volcano was topographically channelled down the volcanic flanks at multiple locations radially around the edifice, steered along pre-existing channels or eroding new ones^13,15^. As they reached deeper waters, the less-confining topography of the seafloor caused currents to spread out forming low-relief morphologies and widespread blanketed deposition over 100 km^62^.

Generating long-runout turbidity currents requires preservation of excess density which can be achieved through a combination of initially high current speeds^63^, large volumes of mobilised material^64^, fine sediment grainsize^65^, erosion leading to flow bulking (ignition)^66^, or a semi-continuous supply of material^67^, all of which might have transpired during, intense fragmentation, low-eruption column collapses, and submarine jetting/fountaining, particularly during a period of sustained column growth. The bulk of sediment (~6.5 km^3^) was likely delivered as semi-continuous supply over a window of ~60 min which provided a sudden release of large volumes of material (1.8 × 10^6^ m^3^/second over 60 minutes).

Bulking through flank erosion (entraining an additional ~3.5 km^3^ as identified by pre- and post-eruption bathymetry^15^ and erosive sedimentological bedforms^13^) would have further enhanced the concentration of these flows, also promoting formation and maintenance of a dense near-bed layer^50,68,69^. High near-bed sediment concentrations were sustained across >100 km, explaining the long flow runout across low-relief areas of seafloor with minimal topographic confinement. Comparable deposit geometries and long-runouts emplaced by terrestrially-sourced pyroclastic density currents triggered by sustained column-collapses or vertical jet dynamics support this interpretation^70^. Given current erosivity, at least proximally, any erupted material subaqueously deposited shortly before the January 15th eruption e.g., ash-fallout on January 13th, was likely eroded, explaining the absence of a preserved record of precursory eruptions between Unit 1 and Unit 2.

Eruption plume analyses indicate maximum MER between ~04:10–05:10, before declining^19,25,30^. The majority of the 6.5 km^3^ of pyroclastic material was deposited onto the seafloor during the ~60-min window of maximum MER, explaining the lack of fine-grained mud capping between black sands. Given this timeframe, it seems unlikely that plume-fallout layers are preserved within the main body of the 2022 eruption deposit^45^. Episodic and waning eruption intensity created variable but overall decreasing delivery of pyroclastic material with time, likely explaining the up-core fining and black sand-silt intervals in Unit 1, with at least six pulses of pyroclastic material delivery recorded (e.g., core SW-80).

### Eruption records in underwater density current deposits

**Fig. 5:**
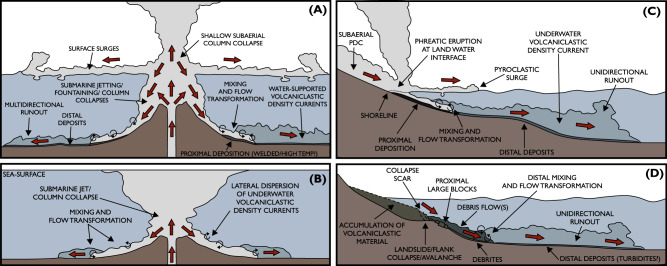
Schematics illustrating the formation of underwater volcaniclastic density currents at different volcanic settings. Schematics are based on our new observations and interpretations and processes discussed in Cas & Wright (1991)^1^, White (2000)^2^, and Freundt (2002)^6^. Red arrows illustrate the direction of travel for all density currents. **A** Formation of underwater volcaniclastic density currents during an explosive shallow-submarine eruption. Collapses of a subaerial or submarine eruption column, alongside jetting and fountaining, from a shallow-vent generates lateral density currents that mix with ambient water to form water-supported volcaniclastic density currents, such as turbidity currents, at some depth or distance from the volcano e.g., Hunga 2022 volcano eruption. **B** Formation of underwater volcaniclastic density currents during a deep-submarine eruption. Collapses of a submarine eruption column or jet from a deep-vent triggers the lateral dispersion of density currents that at some distance from the vent mix with the ambient water to form water-supported volcaniclastic density currents e.g., Havre 2012 eruption^89,90^ and NW Rota-1 2008 eruption^85,86^. **C** Formation of underwater volcaniclastic density currents following the shoreline entrance of a terrestrial-initiated pyroclastic density current (PDC). Turbulent mixing of the hot PDC and ambient water at some depth or distance from the shoreline results in the formation of a water-supported volcaniclastic density currents such as turbidity currents e.g., Soufriere Hills 2003 dome collapse^3^. **D** Mass-wasting events e.g., flank collapse, sector collapse, dome collapse, over steepening of the coastal shelf, remobilises volcaniclastic material laterally. Initially forming debris flows depositing debrites. Mixing with ambient water forms distal water-supported density currents such as turbidity currents e.g., 150 Ka Icod Landslide, Tenerife^95–100^.

Submarine deposits are often the only record of marine eruptions, their triggers, and other associated volcanic processes. The characterisation of the 2022 Hunga Volcano eruption deposits, combined with repeat seafloor bathymetry and seismic records, offers the most comprehensive record of exceptionally rapid, syn-eruptive, pulsed, and multidirectional, delivery of volcaniclastic material during a large explosive (VEI 6) shallow-submarine eruption to date. As such, the spatial distribution and nature of such deposits can act as a guide for interpreting past comparable processes from stratigraphic records at other volcanic sites. This is particularly pertinent as there is an increasing body of work identifying voluminous and far-reaching volcaniclastic deposits and attributing them to density currents generated by large, energetic, and fragmental marine eruptions. However, the efficacy of such deposit-based interpretation depends upon different volcanic mechanisms being distinguishable with no direct observation of the process. Here, we compare the 2022 Hunga submarine deposits with prior exemplar deposit studies that include density currents generated from the ocean-entry of pyroclastic density currents, mass-wasting events, and submarine eruptions (Table 1; Fig. 5).

**Table 1:** Underwater volcaniclastic density current deposit characterisation for different current initiation mechanisms

	Volcano	Volcanic Setting	Event	VEI	Interpreted Trigger	Transported volume	Deposit Geometry (Distribution)	Deposit Extent (Runout)	Deposit Geomorphology	Deposit Structure	Deposit Composition
**Submarine Eruptions**	Hunga Volcano (Tonga, Pacific Ocean)	Shallow-submarine volcano	January 15th, 2022, Eruption	5–6	Low eruption column collapses and low subaerial or submarine jetting and fountaining	~ 6 km^3^ of erupted material ~3.5 km^3^ of eroded and entrained material	Multidirectional	>111 km	*Proximal* – Minimal deposition and erosive bedforms (crescentic scours and linear gullies) on submarine volcano flanks *Medial* – thick ( < 40 m) well-defined lobate deposits (5 km from source) on steep 8–10^o^ slopes *Distal* – widespread, blanketed, with rippled bedforms up to 111 km from source.	*Proximal* – Not sampled *Medial* – Not sampled *Distal* – Stacked sequences of fining upward turbidite silt-sands (turbidite stack) overlain by an ash-fall layer.	Dominated by compositionally homogenous juvenile volcanic glass with little lithic and biogenic material.
	Unknown^9^ (Near Santorini, Aegean Sea, Greece)	Shallow-submarine volcano (unknown depth)	Formation of the Archaeos Tuff	NA	Submarine Jet Collapse	89 ± 8 km^3^	Multidirectional (Origin unknown)	>50 km (Origin unknown)	Infills basins and is locally 150 m thick	Massive-to-diffusely bedded lapilli tuffs and tuffs	Dominated by compositionally homogenous pumice and ash with lesser lithic components
	Black Point^82^ (Lake Russel, California, USA)	Shallow-lacustrine volcano (105 m b.s.l)	13 Ka Black Point eruption	NA	Surtseyan activity and phreatomagmatic explosions	~0.8 km^3^	Multidirectional	>16 km	NA	Dominated by moderate-to-poorly sorted planar laminated beds alternating with climbing ripples. Massive and convoluted beds identified towards the top of the deposit	Dominated by compositionally homogenous basaltic-to-basaltic andesite volcaniclastics
	Pahvant Butte^83^ (Lake Bonneville, Utah, USA)	Shallow-lacustrine volcano (85 m b.s.l)	18.6 Ka eruption	NA	Surtseyan eruption	Unknown	Multidirectional	>25 km	NA	Centimetre-to-decimetre thick moderately-to-poorly sorted ash that records planar laminations, unidirectional ripples, and repeated normally graded beds.	Dominated by volcaniclastic material derived from a single stratified magma chamber
	Havre^89,90^ (Kermadec Arc, New Zealand)	Deep-submarine volcano (900 m b.s.l)	2012 Eruption	1	Extremely dilute suspension flow spread radially from a submarine eruption column	Unknown	multidirectional	>5 km (extent of study area)	Decimetre thick (2–14 cm) deposit. Thickest when sitting on lava flows and caldera floor. Thins on the rims of the caldera.	Decimetre thick massive deposit	Cohesive extremely fine ash. Dominated by glassy vesicular ash with no lithic clasts
	NW Rota-1^85,86^ (Mariana Islands, Pacific Ocean)	Shallow-submarine volcano (512 m b.s.l)	2006 eruptive activity	0	Submarine column collapse	Unknown	Unidirectional	NA	NA	NA	NA
**Water-Entering Subaerial Pyroclastic Flows**	Unknown^55,80^ (Collision zone of paleo-Honshu & Izu-Bonin Arc)	Shallow- submarine volcano (unknown depth)	Formation of the Miocene Nakanai Trough Volcaniclastics	4–5	Major rhyolitic eruptions	NA	NA	150–350 km	NA	Generally massive, unstratified, and moderate-to poorly sorted tuffaceous sandstones	Dominated by compositionally homogenous juvenile volcanic glass and minerals
	Krakatoa^75,76,79^ (Sudra Strait, Indonesia)	Subaerial volcano	1883 Eruption	6	Paroxysmal eruption	~12.4 -30 km^3^	Multidirectional	40 km - based on extent of submarine deposits 80 km - based on the travel distance of pyroclastic surges	Majority of the deposit emplaced within 15 km offshore Krakatau	*Proximal* - composed of a massive and poorly-sorted mixture of pumice and lithics. *Distal* - a rare, well-sorted deposit recording planar laminations and low-angle cross-bedding with tractional structures.	Dominated by pumice and lithic lapilli within a silt-sand ash matrix
	Soufrière Hills Volcano^10^ (Montserrat)	Subaerial volcano	2003 Dome Collapse	3	Dome collapse (block-and-ash flows)	0.2 km^3^	Unidirectional	<40 km	*Proximal* - blocky lobes (60 m thick) within 500 m of the shoreline. *Medial* - metre-to-decimetre thick lobate deposits 5–8 km from shore thinning distally. *Distal* - widespread and featureless deposits from 8-40 km	*Proximal* - normal graded and fines depleted breccia with metre scale blocks present. *Medial* - normally graded fines-poor granules-sands. *Distal* - sand-silt turbidites.	Proximally dominated by compositionally homogenous volcanics with increasing bioclasts distally.
	Stromboli^71^ (Italy)	Subaerial	2022–2023 Volcanic Activity	2	Crater rim/flank collapses, Lava flow front collapses	3.7 × 10^6^ m^3^ (remobilized canyon material)	Unidirectional	NA	Proximally a subaerial erosive canyon extending from the volcanic crater down the entirety of the volcanic flank to the shoreline (1–16 m deep). Immediately beyond the shoreline (and partially subaerial) is a lobate deposit that extends to at least 400 m below sea level. No distal analysis	NA	NA
**Mass-Wasting Events**	Las Candedas Volcano^54,95–101^ (Tenerife, Canary Islands)	Subaerial volcano	150–170 ka Icod Landslide	NA	Sector Collapse	320 km^3^	Unidirectional	860–1300 km	*Proximal* – Failure scar infilled by later lava flows. Minimal deposition and an erosion chute ( ~ 10 km wide and 400 m deep) that extends into the subaerial Icod Valley. *Medial* – Steep-sided elongate deposit with a minimum thickness of 45 m that runs out for 160 km. Peripheral halo of coarse blocks (up to 1.5 km in diameter). Entire deposits surface covered with blocks. Flow structure are present such as linear structures and aligned blocks. *Distal* – Thin and widespread deposition up to 1300 km from source.	*Proximal* – Not sampled *Medial* – Debris avalanche or debris flow deposits *Distal* – stacked sequences of fining upward turbidite sands (turbidite stack) separated by clays	Heterogenous volcanic glass composition. Significant carbonate, organic, and lithic components.
	Ritter Island^92^ (Papua New Guinea)	Partially-submerged volcano ( ~ 100–140 m a.s.l)	1888 Landslide	NA	85 km	13 km^3^	Unidirectional	85 km	*Proximal –* Subaerial and submarine collapse scar. Metre scale dense blocks. Metre-scale mounds are present resulting from seafloor deformation. An incised channel network is present on the margin of these mounds *Medial –* cannot be traced, if present <6 m thick *Distal –* 40 km from source a lobate deposit 15 km across and 16 m thick thins downslope towards a break in slope. Second lobate deposit present following slope break. Beyond the lobate deposits the most distal deposit extends up to 85 km from source and has a sheet like morphology ( < 10 m thick).	*Proximal –* Hemipelagic mud with decimetre blocks poking through. *Medial –* seafloor draped in mud *Distal –* The two lobate deposits are debris flows. The most distal blanketed deposit is a stack of turbidites	*Proximal* – Not Sampled *Medial* – Not Sampled *Distal* – Lobate deposits are comprised of medium-grained volcaniclastic sands. The top few cm of the turbidite deposits comprised a well-sorted, fine grained volcaniclastic sand. Volcaniclastic components are compositionally heterogenous. High bioclastic content.
	Monowai^94^ (Kermadec Arc, New Zealand)	Shallow-Submarine volcano ( ~ 100 m b.s.l)	1998-2007 Sector Collapses	NA	Sector Collapse	0.04–0.09 km^3^	Unidirectional	>5.5 km	Proximal collapse scars (1.5-2 km long and 500–900 m wide). Thickest deposits and 70% of their volume is within 1.5 km downslope of scars. Thinner deposits extend at least 5.5 km from the slide headwall. Downslope of the collapse scar the deposits are smooth with hummocks and levees absent.	NA	Likely fragmented pyroclastic material
	Havre^85,86^ (Kermadec Arc, New Zealand)	Deep-submarine volcano (900 m b.s.l)	Post- 2012 Eruption	NA	Dome Collapse	Unknown	Unidirectional	Unknown	*Proximal* – surrounds existing dome with metre scale blocks present. *Medial to distal* – deposit extends and thins downslope away from the dome	Deposit grainsize decreases distally	Dominated by microcrystalline ash with no lithic clasts.
	Havre^89,90^ (Kermadec Arc, New Zealand)	Deep-submarine volcano (900 m b.s.l)	Post- 2012 Eruption	NA	Partial Caldera wall collapse	Unknown	Unidirectional	Unknown	Proximal scallop-shaped scarp truncating lava flows	Coarse deposits that finer away from the collapse scar	Dominated by microcrystalline ash with no lithic clasts
	Kick em’ Jenny^4,93^ (Grenada, Lesser Antilles	Shallow-submarine Volcano (~200 m b.s.l)	2015 Eruption	0	Partial Flank Collapse	9.6 × 10^3^ m^3^	Unidirectional	18.9 km	*Proximal* – failure scars and an erosion chute >100 m wide and >1 km long	NA	NA

Submersion of terrestrial-initiated pyroclastic density currents (PDCs) at the shoreline is a common feature at near-shore volcanoes (e.g., following strombolian eruptive activity at Stromboli 2019–2022^71–73^ and eruption column collapse at La Soufrière^74^). Subsequent transformation of PDCs into underwater volcaniclastic density current is recorded in numerous submarine deposits (e.g., 1883 Krakatau eruption^75,76^) and has been observed directly (e.g., 2003 dome collapses at the Soufriere Hills Volcano, Montserrat^10,77^). Deposits of water-entering PDCs are typically unidirectional when parent flows are triggered by smaller, localised, events including dome collapses^3^ and crumbling lava flow fronts^78^. In contrast, larger and more explosive eruptions can trigger radial dispersions of water-entering PDCs and ensuing underwater currents (e.g., following column collapse during the VEI 6 1883 Krakatau eruption)^76,79^. For minor, low-explosivity, volcanic activity that triggers discrete shoreline-crossing PDCs (e.g., Soufriere Hills Volcano dome collapses or recent activity at Stromboli), the subsequent unidirectional underwater currents have shorter runouts than identified at Hunga (Table 1). For larger eruptions (of similar VEI or greater to Hunga), density currents have been inferred to travel equivalent (or greater) distances, albeit with primarily unidirectional runouts except in the case of relatively small ocean island volcanoes^55,80^. Near-shore deposition and only minor subaerial erosion associated with individual water-entering PDCs from small and low-explosivity eruptions differs greatly from the proximal flank erosion at Hunga (Fig. 1). Distally, the Hunga 2022 deposits record stacked fining-upward sequences distinct from the single-layered, typically normally-graded and massive deposits recording infrequent planar and ripple laminations observed in deposits sampled offshore Montserrat^10,76^. This differing deposit structure likely reflects the contrast between continuous, pulsed, and multidirectional flows sustained by high eruption rates from a shallow vent (e.g. Hunga), as opposed to a discrete PDC generated during small-scale volcanic activity (e.g. Montserrat).

Submarine volcanoes record a variety of eruption styles that trigger underwater-density currents^81^. Very-shallow (<~100 m b.s.l.) Surtseyan eruptions, e.g., the 13 ka Black Point eruption^82^ and 18.6 ka Pahvant Butte eruption^83^, driven by explosive phreatomagmatic fragmentation generate density currents with multidirectional runout, creating deposits that fine upwards, with planar and ripple laminations, comprising compositionally homogenous volcaniclastic material. However, they have far-shorter runouts (>14–25 km) likely resulting from the order of magnitude smaller eruptive volumes (e.g. 0.8 km^3^) than the 2022 Hunga eruption. This underscores the control of eruption volume and mass flux in controlling resultant density current runout.

Magmatic volatile-driven explosive shallow-submarine eruptions (<500–1000 m b.s.l.) can trigger density currents via combinations of submarine eruption-column collapse and fountaining^2,84^. The size and runout of currents triggered during relatively small eruptions (VEI 0–2; e.g. 2008 North-West Rota-1 eruption), are far smaller than those identified at Hunga^85,86^. No field studies document all diagnostic criteria for more voluminous eruptions at these depths. Proximal deep-sea coring and in-situ deposits often record massive (or diffusively bedded) pumice-rich deposits^9^, may display upward-coarsening^87^, and, in some cases, evidence welding indicative of heat-retention and gas-supported flow^88^. Challenges in sampling close (<20 km from the volcano) to Hunga Volcano prevents direct comparison, but we see no evidence of high-temperature emplacement. Furthermore, whilst eruptions of similar or greater magnitude to Hunga 2022 (e.g., Archaeos Tuff forming event) can generate multidirectional currents with similar runouts to Hunga, they record no distal preservation of pulsed flows in the form of stacked fining upward sequences^9^.

At deep-submarine volcanoes (>500–1000 m b.s.l), suppression of explosive magmatic fragmentation due to water-pressure largely limits density current formation to triggering by mass-wasting. However, at these depths fuel-coolant interactions can trigger explosive eruptions such as the 2012 Havre eruption, where the lateral dispersion of dilute volcaniclastic density currents from a submarine eruption column emplaced multidirectional, thin (2–14 cm), and massive volcaniclastic deposits of lithic-poor cohesive ash^89–91^. However, the short runout (>5.5 km), and absence of both fining-upward sequences and erosional bedforms distinguish these deposits from those observed at Hunga.

Mass-wasting events at marine volcanoes can produce underwater volcaniclastic density currents, even during quiescent periods absent of eruptive activity^92^. Small mass-wasting events, associated with localised dome and caldera-wall collapses, frequently occur at submarine volcanoes including Havre^89,90^, Kick em’ Jenny^4,93^, and Monowai^94^. Larger mass-wasting events relate to volcanic flank collapses such as the 150 ka Icod landslide, Tenerife^95–100^, and the 1888 Ritter Island lateral collapse, Papua New Guinea^101^. Despite variation in mobilised volume (<0.1–320 km^3^) and runout (5.5–1300 km), mass-wasting deposits are consistently unidirectional and share congruent morphologies: proximal fault scar, proximal large blocks (decimetre-to-kilometre diameters), and a medial-to-distal elongate and spreading lobate morphology, unrecognised in the Hunga bathymetry. Medially-to-distally, mass-wasting deposits are characterised by debrites^90,101^, unrecorded in any Hunga sediment cores. The distal limits of mass-wasting deposits commonly record volcaniclastic turbidites e.g., Ritter Island^101^ and Icod landslide^54^. Distal turbidites of the Icod landslide feature stacked fining upward sequences like those observed in the Hunga 2022 deposits; however, these sequences are attributed to successive phases of flank collapse, which can be distinguished via unidirectional runout and heterogenous lithology. Mass-wasting deposits, specifically those associated with secondary remobilisation of previously erupted volcaniclastics, record heterogenous volcanic compositions and high proportions of lithic and biogenic components – in contrast to the dominant homogenous juvenile composition and minimal lithic and biogenic proportions of the Hunga deposits.

The 2022 Hunga submarine eruption deposits document density currents generated during a large (VEI 5-6), explosive shallow submarine eruption characterised by high mass eruption rates (MER) and large erupted volumes (>6.5 km^3^). A clear distinction exists between these deposits and deposits associated with density currents triggered by small-volume and low mass-flux volcanic activity e.g., shoreline-crossing PDCs associated with dome collapses, minor flanks collapses, Surtseyan eruptions, or low-explosivity deep-submarine eruptions (Table 1), recognised by deposit extent and dispersion. For volcanic activity of comparable size and mobilised volumes to the January 15th eruption, density current runouts, dispersal, and deposit structures are far more analogous, regardless of current initiation mechanism (large volume mass wasting events, explosive submarine eruptions, and shoreline crossing PDCs; Table 1). Despite some shared characteristics, we conclude that deposits of density currents triggered by the rapid and sustained supply of volcaniclastic material directly onto the submerged flanks of the volcano during a large explosive shallow-submarine eruption, can be reliably distinguished from other density current initiation mechanisms. This is on the basis of combined multidirectional dispersion, extensive runout, stacked fining-upward sequences (following unsteady and pulsed delivery of material), and the proximal erosive scours and bedforms that sculpt the flanks of the volcano (trains of crescentic scours and incised channels). However, due to the size of the January 15th eruption, not all observations or diagnostic criteria from this event will necessarily be transferrable to smaller eruptions.

We have shown that syn-eruptive and semi-continuous supply of large volumes of erupted pyroclastic material delivered vertically onto the upper submerged flanks of a volcano over a period of minutes-to-hours generates exceptionally fast underwater volcaniclastic density currents capable of maintaining a dense component for over 100 km. Their sustained power across vast distances poses challenges to the safe routing of subsea infrastructures such as telecommunication cables (that carry >99% of digital data traffic), highlighting a need for greater investment in understanding the associated hazards and implementing back-up systems in active marine volcanic regions.

Density currents emplaced during the 2022 Hunga eruption are distinct from deposits related to other initiation mechanisms, identifiable from their pulsed nature, long-runouts and multidirectional dispersion. Beyond density current behaviour, the 2022 Hunga submarine eruption deposits preserve a record that correlates with observed mass eruption rate changes. The long-term eruption record held by these submarine deposits are a tool to unravel past eruption dynamics.

Our findings enable the development of diagnostic criteria to identify the triggering mechanism for submarine volcaniclastic density currents created by large volcanic eruptions, enabling more robust interpretations of volcanic activity and associated hazards based on deposits from many other marine volcanoes worldwide. These insights contribute to improving existing knowledge of eruption processes and shallow-submarine volcanoes globally.

## Methods

### Field Survey And Data Acquisition

Post-eruption acquisition of bathymetric data, seafloor imagery, and sediment cores was previously conducted during the TESMaP (Tonga Eruption Seabed Mapping Project) TAN2206 research cruise onboard the R/V Tangaroa to the site of the Hunga eruption in April to May 2022^14,15,102^.

### Bathymetric surveys

Multibeam bathymetric data detailing the post-eruption topography of Hunga Volcano, and the surrounding seafloor (Fig. 1) was collected using a Kongsberg Simrad EM302 multibeam echosounder on the RV Tangaroa during the TAN2206 Cruise in April and May 2022^14,15,102^. Seafloor elevation changes relating to the January 15th, 2022, eruption of Hunga volcano were determined by comparing this post-eruption bathymetric data with a digital elevation map (DEM) comprised of pre-eruption bathymetric data acquired both remotely and on two separate research expeditions^13,15^: (1) 2017 satellite-derived bathymetry^103^, (2) the May 2016 FK160407 Expedition on the RV Falkor using a Kongsberd Simrad EM302^104^, and (3) November 2015 small boat survey using a WASSP multibeam echosounder^16^. The vertical resolution of these data is typically 1% of the water depth. The pre-and post-bathymetric data is available online at Zenodo^102^. Bathymetric data from the different surveys were horizontally grided to 50 × 50 m. These pre- and post-eruption DEMs were imported into ArcPro GIS where the post-eruption surface was subtracted from the pre-eruption surface to create an elevation difference raster of the seafloor around Hunga that visualises regions of erosion (negative values)and deposition (positive values)^13,15^. As the South-West transect profile crosses areas of seafloor not covered by the 2022 bathymetric survey, we utilise the Global Multi-Resolution Topography (GMRT; dataset Version 4.2, released May 2024^105^), to illustrate topographic change across the transect (Fig. 1). The location of Hunga volcano on the Tonga-Kermadec arc, and its relation to local plate tectonics, was illustrated in Fig. 1a using General Bathymetric Chart of the Oceans (GEBCO) 2025 Grid^106^ and the U.S. Geological Survey plate boundary data^107^.

### Seafloor imaging

Seafloor imagery was collected using the National Institute of Weather and Atmosphere’s (NIWA) Deep Towed Instrument System (DTIS). The DTIS continuously records high-definition digital video at 1080p at 60 FPS, whilst synchronously taking high-definition (24 Megapixel) images every 15 s. DTIS location was tracked in real-time using the KONGSBERG Ultrashort Baseline Transponder. Seabed position of the DTIS was plotted in real-time using the Ocean Floor Observation Protocol (OFOP) software.

### Sediment core collection and non-destructive analysis

Collection of seafloor sediment cores sampling the January 15th, 2022, Hunga Volcano eruption deposits was performed using an Ocean Instruments MC-800 multicorer system. Coring was carried out at eleven sites encompassing proximal-to-distal distribution of erupted volcanic material around the volcano and along two dedicated transects away from the vent (Fig. 1). Specific sites were targeted for coring, including locations of known international and domestic subsea telecommunication damage and/or burial (Fig. 1). Multicorer position was tracked during deployment using a Kongsberg HiPAP 500 ultra-short baseline (USBL) system. Cores were sub-sampled from the recovered wide multicore tubes with narrower 5 cm-wide push-core tubes (80 cm long polycarbonate core liners) then sealed with absorbent foam and capped. The cores were CT scanned at Boulcott Hospital in Wellington, New Zealand, then bisected and parted longitudinally using cheese-wire to create two core-halves with flat surfaces suitable for imaging, alongside visual and sedimentological analysis. One core-half from each sediment core was delivered to the British Ocean Sediment Core Research Facility (BOSCORF) located within the National Oceanography Centre (NOC), Southampton, UK.

At BOSCORF all sediment cores were visually logged: recording sedimentary structures, sedimentary architecture, composition, lithology, and colour to begin identifying subunits and facies within the deposits (Supplementary Fig. 1). Visual grain size classification utilised a grain size comparator, which records the coarsest 5% of the grain size distribution^108^.

High-resolution imaging of the sediment cores was performed using a Geotek-Multi Sensor Core Logger (MSCL) line scan Core Imaging System at BOSCORF. Imaging provides a precise digital archive of the sediment cores before colour changes associated with oxidation become prevalent or before material is removed for further analysis. Digital modification of the images (e.g., brightening or changes in contrast) was performed in CorelDRAW or ImageJ, to aid visual identification of composition and grain size changes. Back-scatter electron images of sediment core material were taken using the Hitachi TM4000Plus Mk II Tabletop Scanning Electron Microscope at BOSCORF (Fig. 3).

X-ray computed laminography images were acquired using a Geotek ScoutXcan X-ray scanner at BOSCORF. The system is equipped with a 65 W Thermo Kevex 130 kV microfocus X-ray source with a tungsten target, and a Varex Imaging flat panel detector featuring a 1536 × 1920-pixel matrix. For this study, the X-ray tube was operated a 102 kV and 350 μA. A 0.5 mm copper (Cu) filter was positioned in the beam path to attenuate low-energy X-rays and enhance image contrast. The detector was operated in 2 × 2 binning mode. The resulting beam spot size was 54 μm, and the effective image pixel size was 82 μm. Computed laminographic images were reconstructed from the raw radiographic projection data acquired for each core sample using the Geotek Reconstructor proprietary software. The reconstructed laminography images represent slices from different planes along the long axis of the core samples.

Sediment cores were analysed using the Itrax μX-ray Fluorescence (XRF) core scanner with the aim to fingerprint the geochemical signature of the eruption deposits, constrain deposit subunits, correlate between cores, and differentiate the 2022 deposits from the pre-eruption seafloor material (Supplementary Fig. 5). Cores were scanned using a COX Analytical Systems Itrax XRF Scanner (Settings at 30 kV 30 & mA, 15 s exposure time and 1000 μm step size)^109^.

XRF element intensities and element intensity ratios enable a qualitative geochemical comparison between the sediment cores. The presence and abundance of certain elements can be used as proxies for geological and environmental processes^33^. With the volcanic origin of the deposit material^13,15^ and previous geochemical studies at Hunga Volcano and neighbouring volcanoes recording basaltic to andesitic compositions^18^, we focus on iron (*Fe*), potassium (*K*), and titanium (*Ti*), to identify the 2022 Hunga eruption deposits. All three elements have previously been used as indicators of volcanic material in sediment records such as ash and tephra layers^110–112^. With high potassium related to acidic igneous sources, and iron and titanium linked to basaltic igneous sources, ratios of these elements (e.g., *K/Ti* and *Fe/*Ti) assess the contribution of acidic and basaltic sources to the sediments^113–115^. Based on preliminary analyses of the Hunga sediment cores at OTAGO University (New Zealand) we find that due to its conservative nature titanium (*Ti*) provides the best element to normalise to. We also consider chlorine (*Cl*) intensities to distinguish between freshly deposited submarine sediments and the older material they emplace upon. The intensity of chlorine, a major component of seawater, has previously been used as a direct proxy for interstitial seawater content within marine sediment cores^34^. As freshly supplied submarine sediments deposit onto pre-existing material they compact the older deposit lowering both porosity and amount of interstitial seawater. This compaction is recorded in the μXRF as a decrease in Cl intensity in the older compacting sediments relative to the newly deposited material above^34^. In addition, potassium (*K*) is also suitable for identifying weathered material due to its abundance within illite clays^33^. To address the possibility of variations in elemental excitation and scattering that can occur in wet sediments or those with significant grain size changes we normalise all elemental counts to the incoherent plus coherent (inc+coh) scattering^116^.

### Grain size analysis

Quantitative grain size analyses of the Hunga eruption deposits were carried out using a Malvern Mastersizer 3000 and Malvern G Hydro accessory Unit at the NOC, Southampton. The Malvern 3000 utilises laser diffraction to detect particle grainsizes between 0.01 and 3500 μm and calculate particle size distributions within a sample. Grainsize analysis was conducted to aid characterisation of the 2022 Hunga eruption deposits, understand deposit evolution with distance (proximal-to-distal) and time (up-core), and constrain deposit forming processes^117–119^. Samples of the 2022 Hunga eruption deposits were taken from cores along the SW-transect (Fig. 1, SW-64, SW-80, SW-100, and SW-111) at centimetre intervals from the top of the core to the base of the eruption deposits. Each sample comprised approximately 1 cm^3^ of material. No washing of the sample was done prior to analysis to best reflect the true particle sizes at deposition. Samples were mixed with reverse osmosis (RO) water and shaken to ensure suspension of all sediment before being added to the Malvern G Hydro accessory Unit. During the analyses, a laser target obscuration between 2 and 20 % was achieved for each sample. Ten measurements were carried out on each sample, from which an average sample grain size distribution profile was built, providing grain size properties for each sample (e.g., mean, sorting, proportions of clay, silt, sand etc.). Grain size distribution contour plots for the south-west transect sediment cores were created using MATLAB (Fig. 3).

### Glass shard analysis

Individual volcanic glass shards were extracted from sediment core SW-80 and were analysed using high resolution electron probe microanalysis (EPMA) with an aim to identify the composition of the magmas involved in the 2022 Hunga eruption and record any up-core compositional changes.

Twelve samples were taken from SW-80 at a variety of depths and from different facies. The samples were wet sieved using Milli-Q water (purified water) to obtain the grain size fraction 64–125 μm. To break-up clays and remove them from the surface of the volcanic glasses, the sieved samples were placed in small glass containers (28 ml) filled with Milli-Q water and added to an ultrasonic bath for 15 min. After 15 min the samples were removed from the bath, and the Mili-Q water was pipetted out. These steps were repeated. Following the final pipetting the wet samples were placed in an oven at 30 °C until all water had evaporated from the sample.

Dried samples were sent to Royal Holloway University (London, UK), where they were prepared for quantitative microbeam analysis adapting the methods described in Hall and Hayward^120^. Blank resin stubs were created using *EpoThin 2* Resin and *SamplKups*. A standard drill was used to drill 3 × 3 mm holes into the stubs. The stub was then secured onto a glass slide and silicon sheeting using Vaseline. Each of the twelve samples was placed into a predrilled hole. The drilled holes were subsequently filled with resin and cured in a pressure chamber. To section shards a P2500 paper was used. The stub was polished using Al_2_O_3_ powder and a Texmet polishing cloth.

EPMA analysis was conducted on the Cameca SX100 electron probe microanalyser at the Tephra Analysis Unit located in School of Geosciences, University of Edinburgh, UK. The methods used here are outlined in detail in Hayward^121^. All sample analyses were performed using a 5 μm diameter beam at 15 kV External glass standards: (basalt BCR-2G and LIPARI Rhyolite) were run daily before and after each session of EPMA to check values were within error of certified values. For each sample 15°C20 shards were analysed. Backscatter SEM images were used to identify crystalline glass shards. Highly crystalline glass shards were excluded from EPMA. For glass shards where minor crystals were present, EPMA was performed well away from the crystals. Where possible analyses were conducted as close to the centre of the shards as possible (avoiding crystals) to guarantee that the beam was not sampling shard edges (curved surface) or the stub. Shards of all sizes were analysed to ensure the best representation of glass compositions in the sample.

### Componentry

Five samples were collected from sediment core SW-64 at a variety of depths and facies, from both the submarine eruption deposits and pre-eruption seafloor. Samples were wet-sieved to obtain the grainsize fraction >64 μm and to remove clay material coating the deposits. Dried samples were sent to Royal Holloway University (UK), where they were mounted into resin stubs, following the same methods described in Hall and Hayward^120^. Componentry analysis was conducted at both the University of Auckland, New Zealand (November 2024), and NOC, UK, (2025). Analyses were done via particle counting to a minimum of 300 grains using the TESCAN CLARA High-Resolution Field-Emission Gun Scanning Electron Microscope (FEG-SEM) and Leo 1450VP Scanning Electron Microscope at the University of Auckland and NOC, respectively. Componentry was based on macroscopic external characteristics (morphology, vesicularity and crystallinity) and geochemical composition. Geochemical composition was determined from energy-dispersive X-ray spectroscopy (EDS), using the Oxford instruments 65 mm^2^ active area energy dispersive spectrometer and Oxford instruments X-act 10 mm^2^ area SEM-energy dispersive spectrometer at the University of Auckland and NOC, respectively.

## Supplementary information

Supplementary Information

Transparent Peer Review file

## Source data

source data

## Data Availability

All Coordinates, seafloor photos, core logs, core photos, laminographs, and SEM photos, are provided in the supplementary information document. The electron microprobe analysis, grainsize, Itrax μX-ray Fluorescence (XRF), and componentry data generated in this study are provided as a source data file within the supplementary material. Pre- and post-eruption bathymetric data of Hunga volcano and the surrounding seafloor can be accessed in Zenodo (https://zenodo.org/records/7456324)^102^. Global Multi-Resolution Topography (GMRT) data is open access (https://www.gmrt.org/about/#access)^105^. General Bathymetric Chart of the Oceans (GEBCO) gridded ocean land and terrain models is open access (gebco.net/data-products/gridded-bathymetry-data)^106^. U.S. Geological Survey (USGS) plate boundaries dataset is open access (https://earthquake.usgs.gov/learn/plate-boundaries.kmz)^107^. Source data are provided with this paper.
