# Supplementary material for: Far-reaching volcaniclastic density current deposits as evidence of explosive marine eruptions
Source: Nat Commun. 2026 Apr 30;17:5919. doi: 10.1038/s41467-026-71658-8 (PMC13338445; doi:10.1038/s41467-026-71658-8)
Supplement: Supplementary file 1 — Supplementary Information [file 41467_2026_71658_MOESM1_ESM.pdf]

Supplementary Information

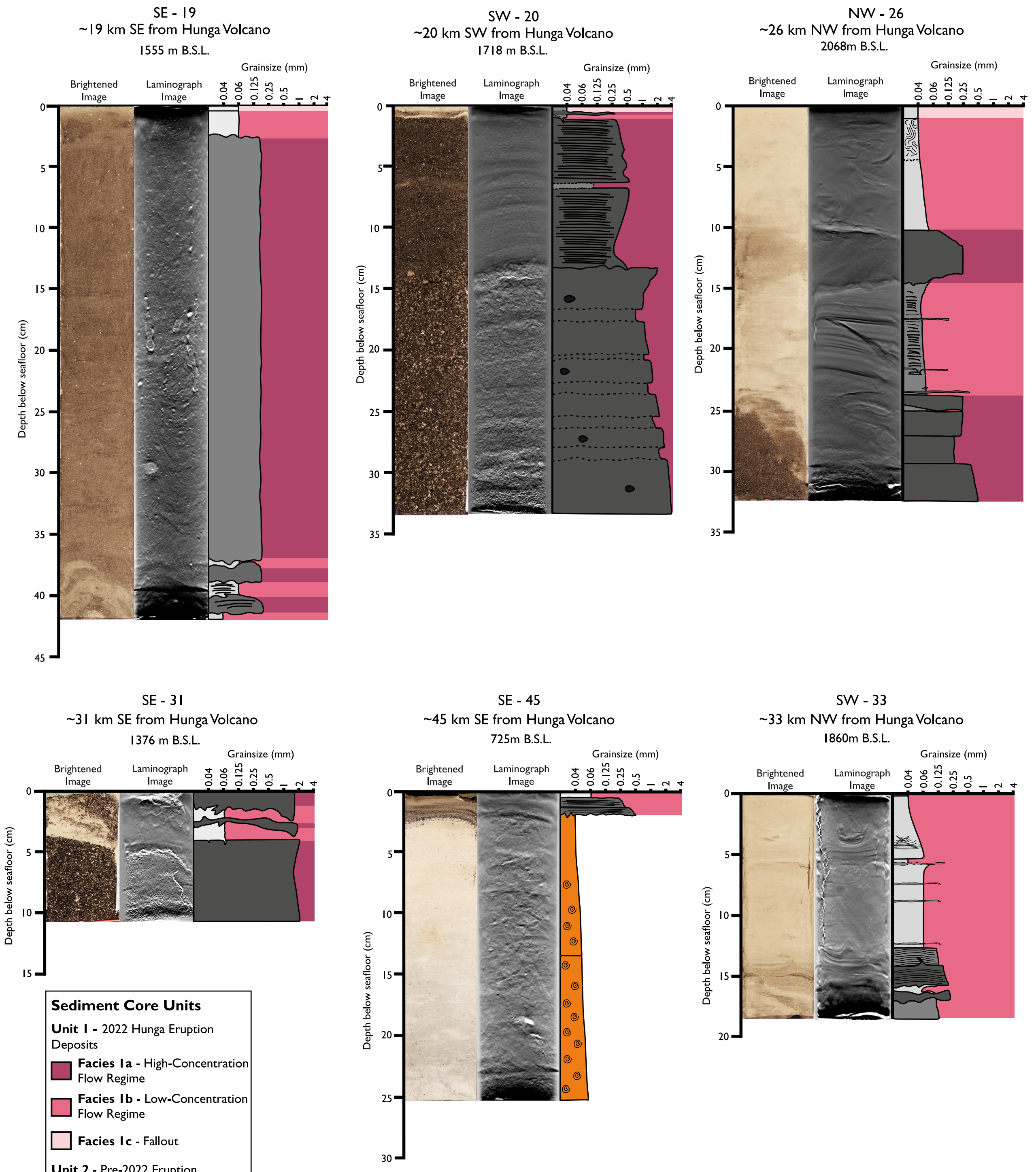

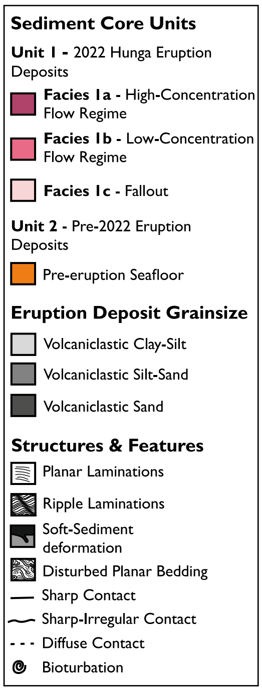

Supplementary Figure 1. 2022 Hunga eruption deposits. Brightened images, laminogrpahs, and visual logs of all eleven sediment cores collected on the TESMaP TAN2206 Research cruise. Locations of each core relative to the others can be seen in Figure 1. B.S.L. - below sea level. Subunits 1a, 1b, and 1c are identified - refer to legend

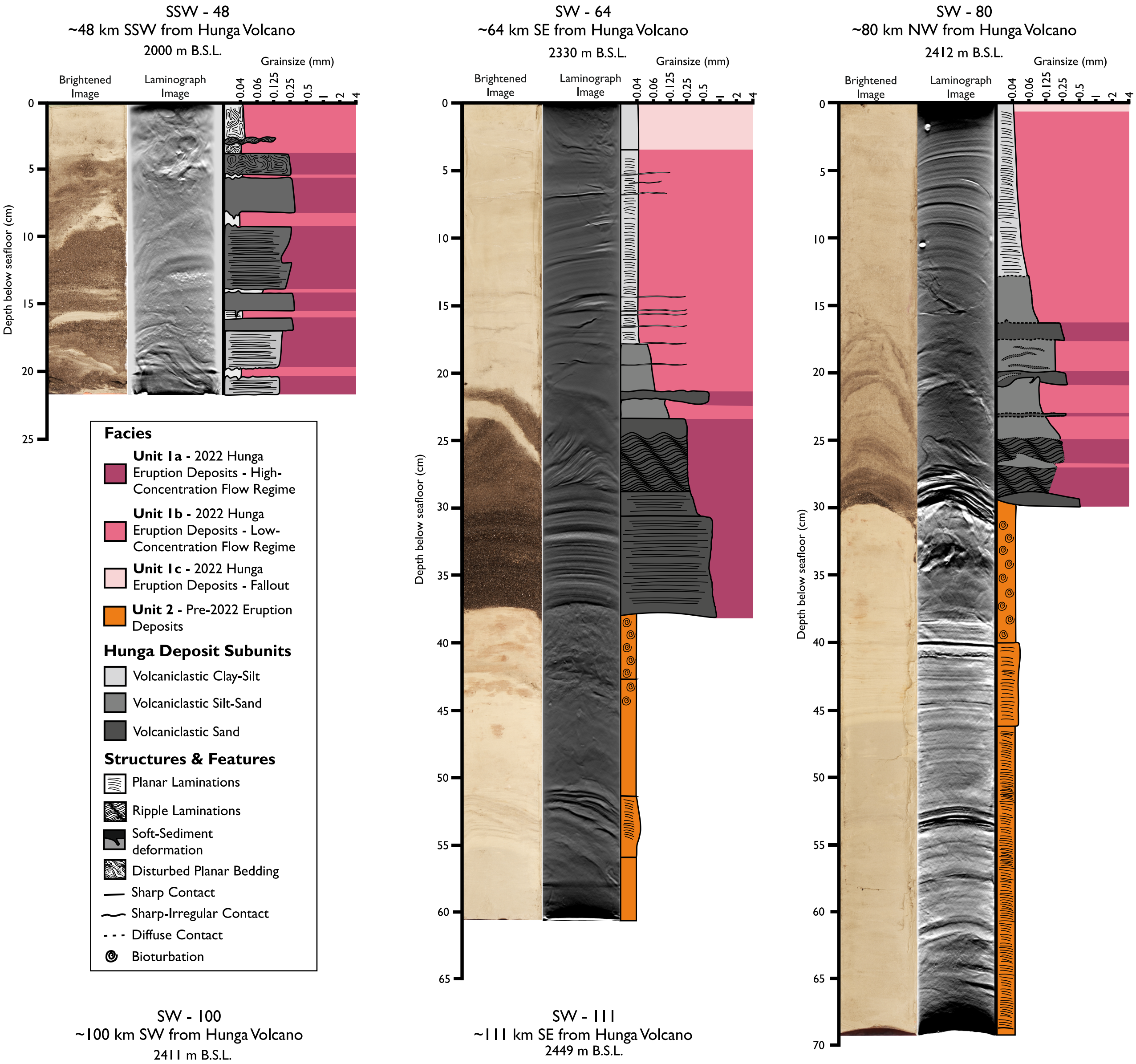

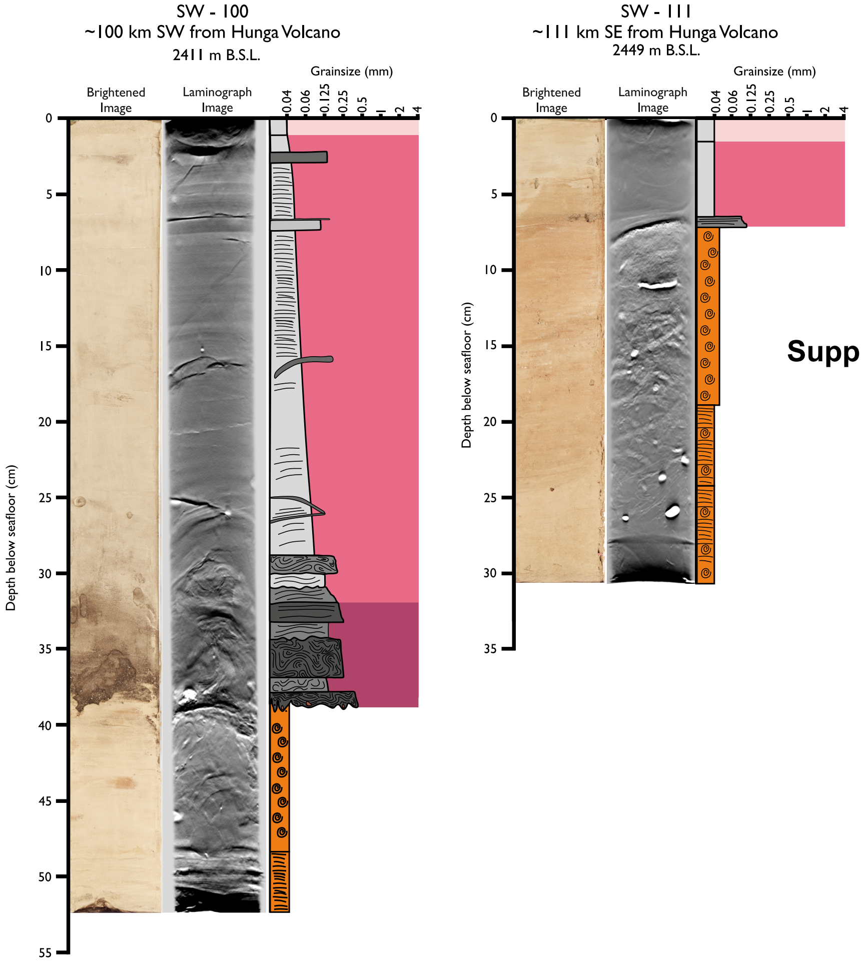

Supplementary Figure 1 Continued

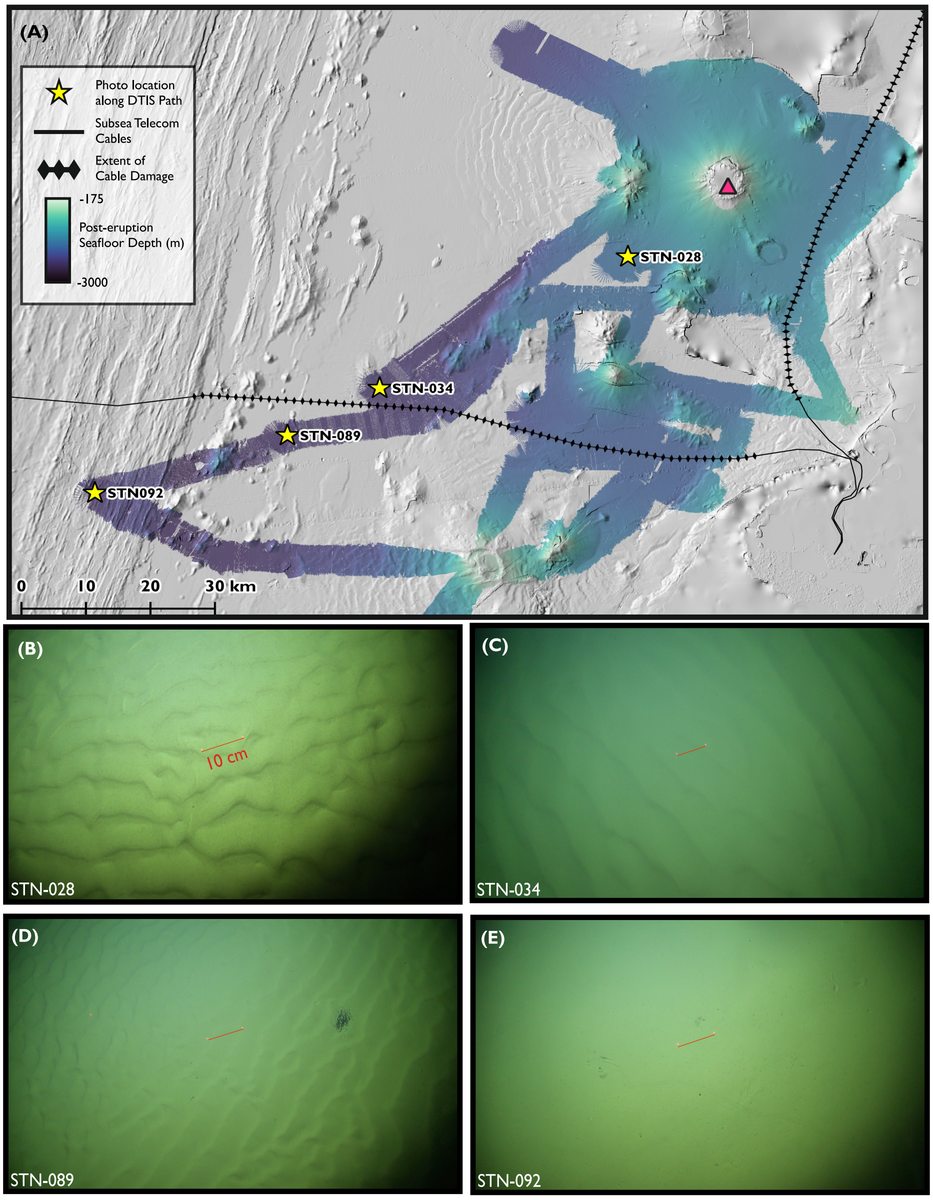

Supplementary Figure 2. Seabed images along the Southwest transect following the January 15th, 2022, Hunga volcano eruption. Images collected using NIWA's Deep Towed Instrument System (DTIS). (**A**) Location of images taken along DTIS pathways displayed on bathymetric data collected 3 months after the eruption of the TESMaP TAN2206 research cruise[1-4], overlain on Global Multi-Resolution Topography (GMRT)[5]. (**B-D**) Muddy sediments with well-defined ripples present over 85 km from Hunga volcano. (**E**) Featureless muddy sediment images west of high-relief ridges. The red scale bar in each inset is 10 cm.

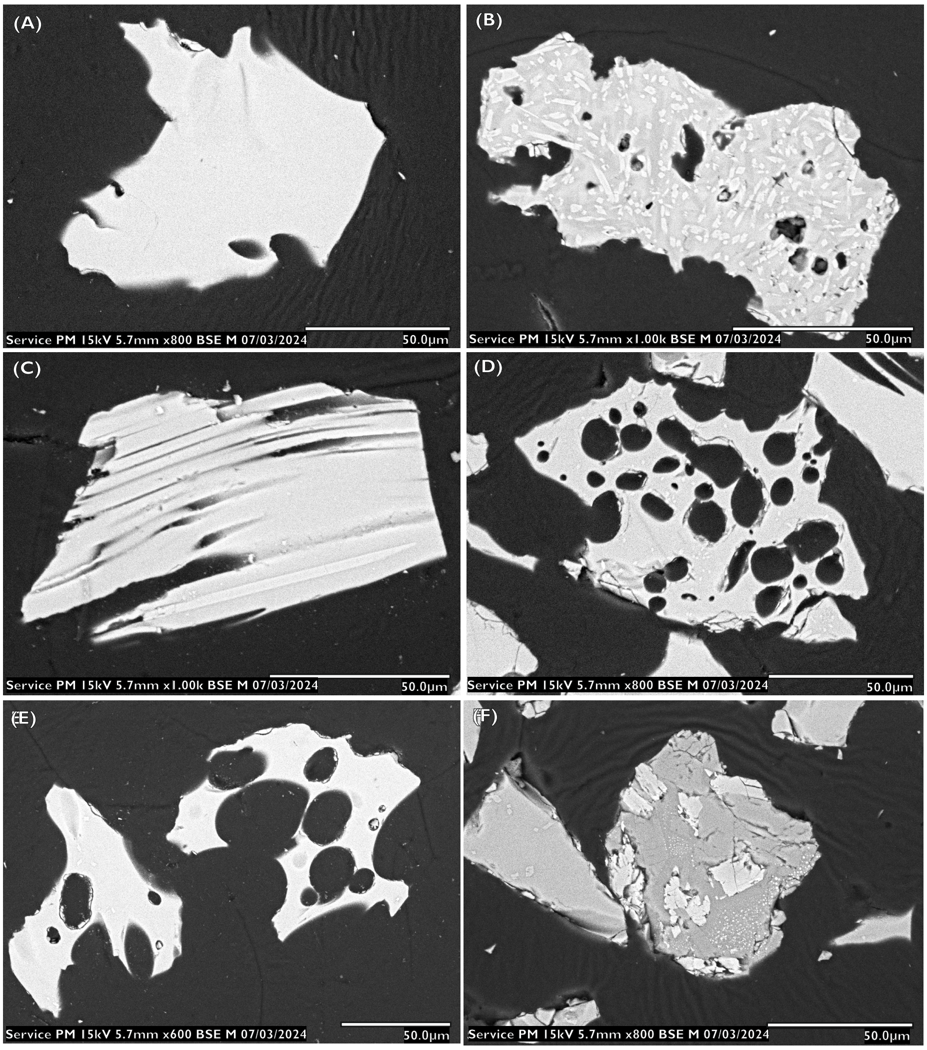

Supplementary Figure 3. Scanning electron microscope (SEM) images of material within the 2022 Hunga volcano eruption deposits from sediment core SW-80. (A) Massive and blocky glass. (B) Microlite rich glass. (C) Tube pumice. (D) Bubble rich glass. (E) Irregular and bubbly glass. (F) Lithic.

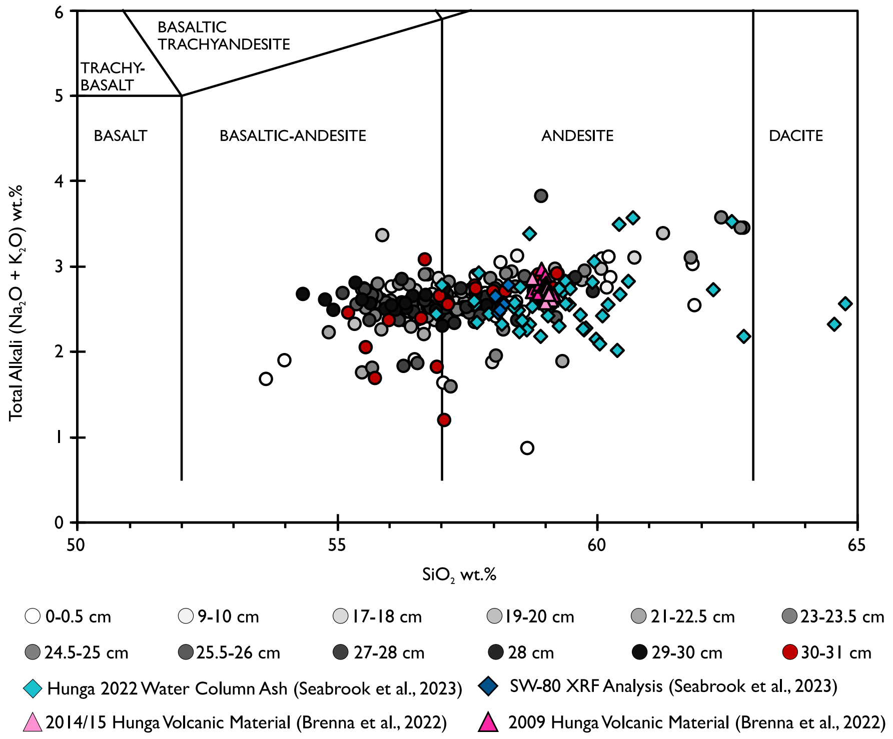

Supplementary Figure 4. Volcanic glass composition of the 2022 Hunga submarine eruption deposits. Total Alkali Silica (TAS) diagram plotting electron probe micro-analyses (EPMA) measurements of density current deposit samples taken from core SW-80 (circles). Also plotted are XRF analyses of SW-80 (dark blue diamonds)[3], submarine ash-samples (light blue diamonds)[3] collected 3 months after the eruption, and tuffs from the 2009 (light pink triangles) and 2014/15 (dark pink triangles) events at Hunga volcano[6].

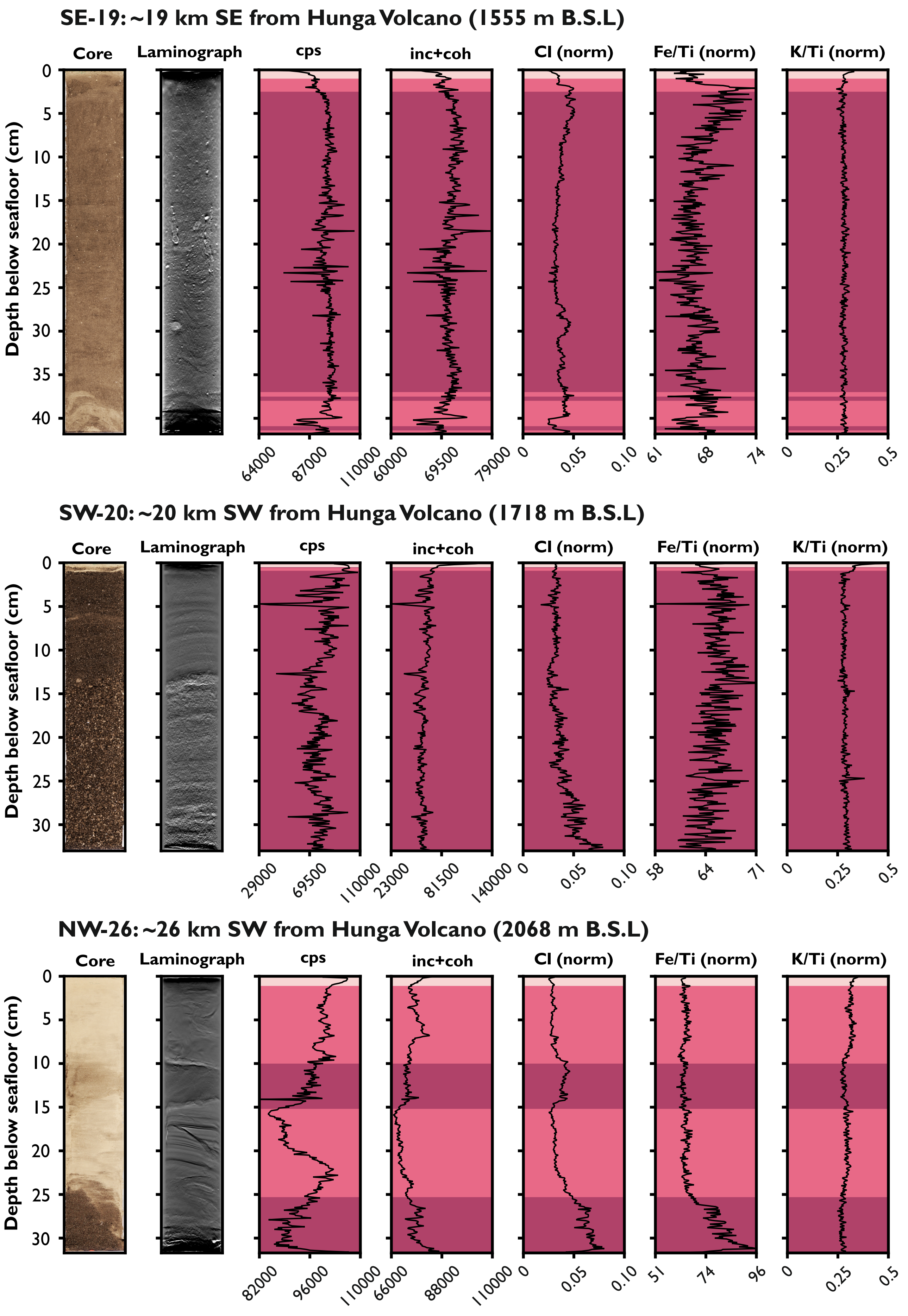

Supplementary Figure 5. Geochemical signature of the 2022 Hunga eruption deposits. Core panels for all sediment cores collected on the TESMaP TAN2206 Research cruise. Brightened cores images, laminographs and down-core Itrax micro-XRF. Subunits 1a, 1b, and 1c identified - see legend.

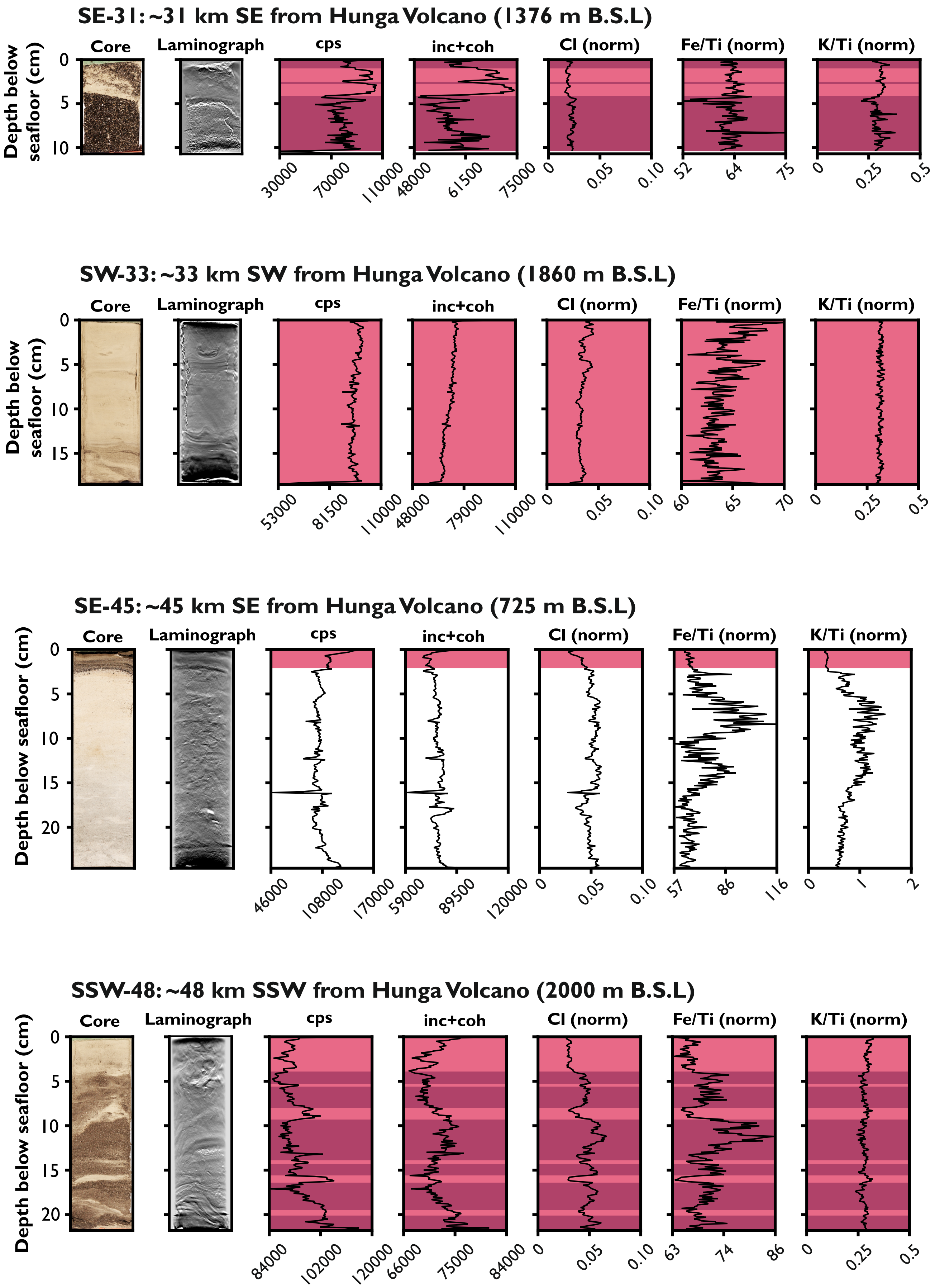

Supplementary Figure 5 Continued

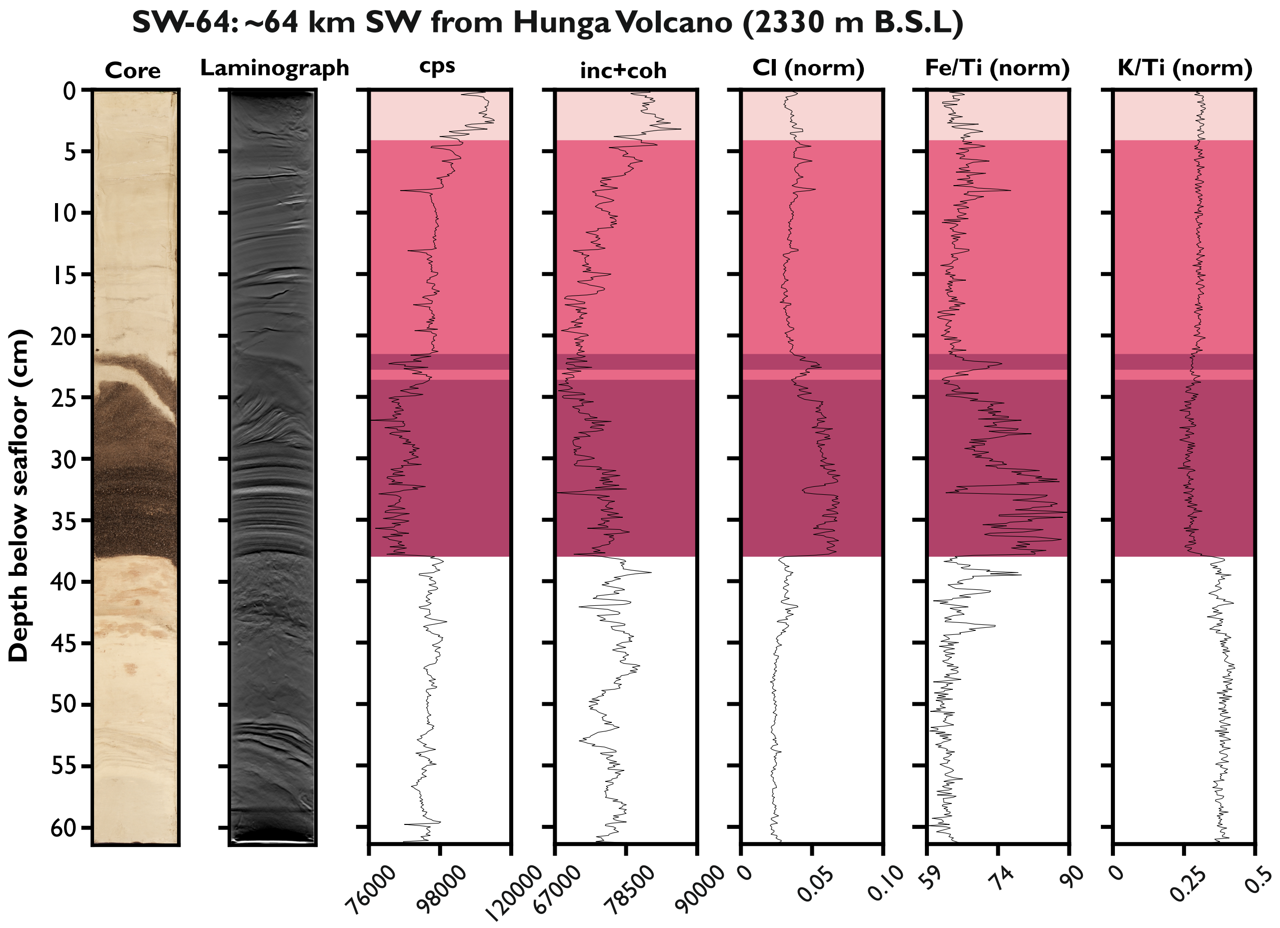

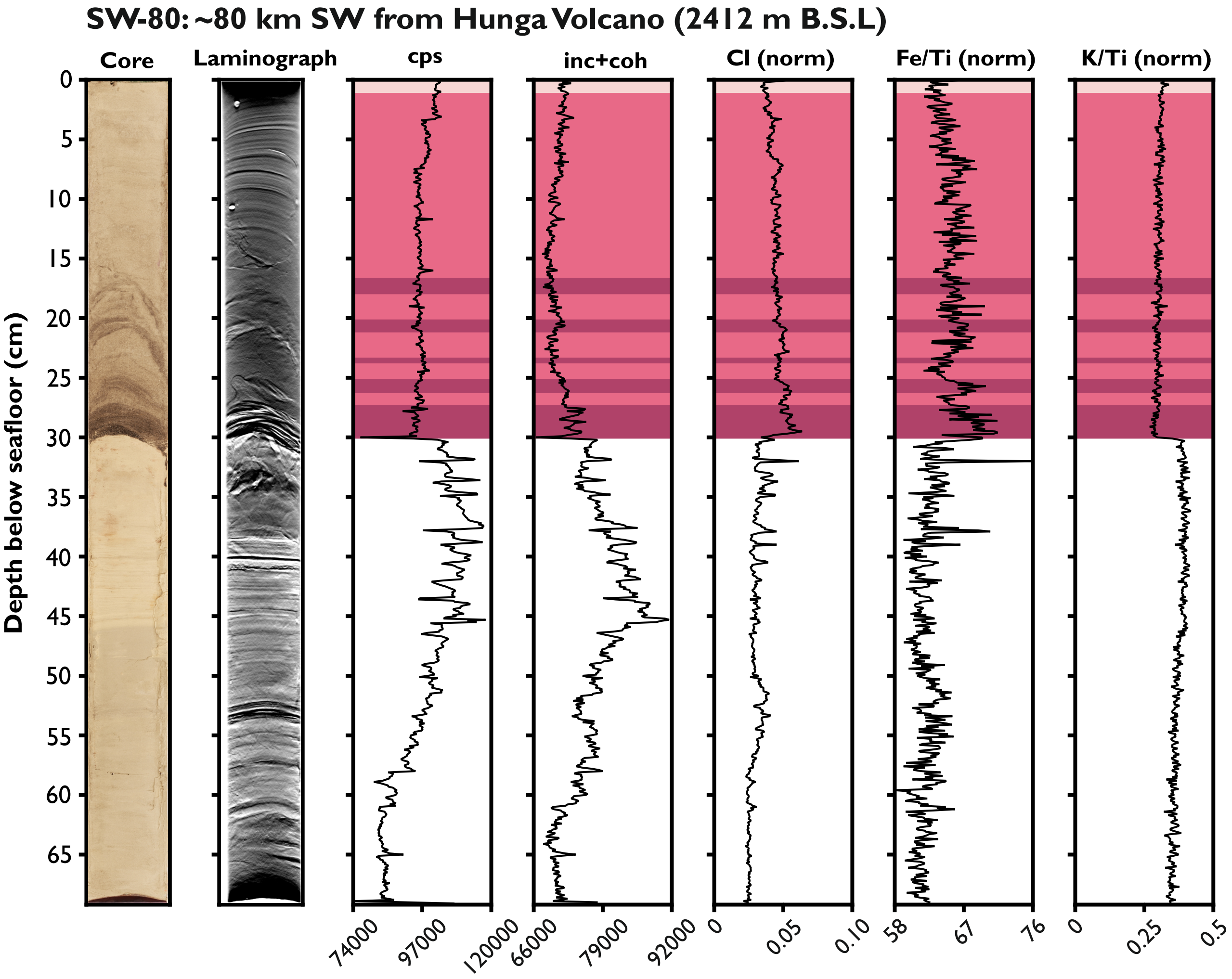

Supplementary Figure 5 Continued

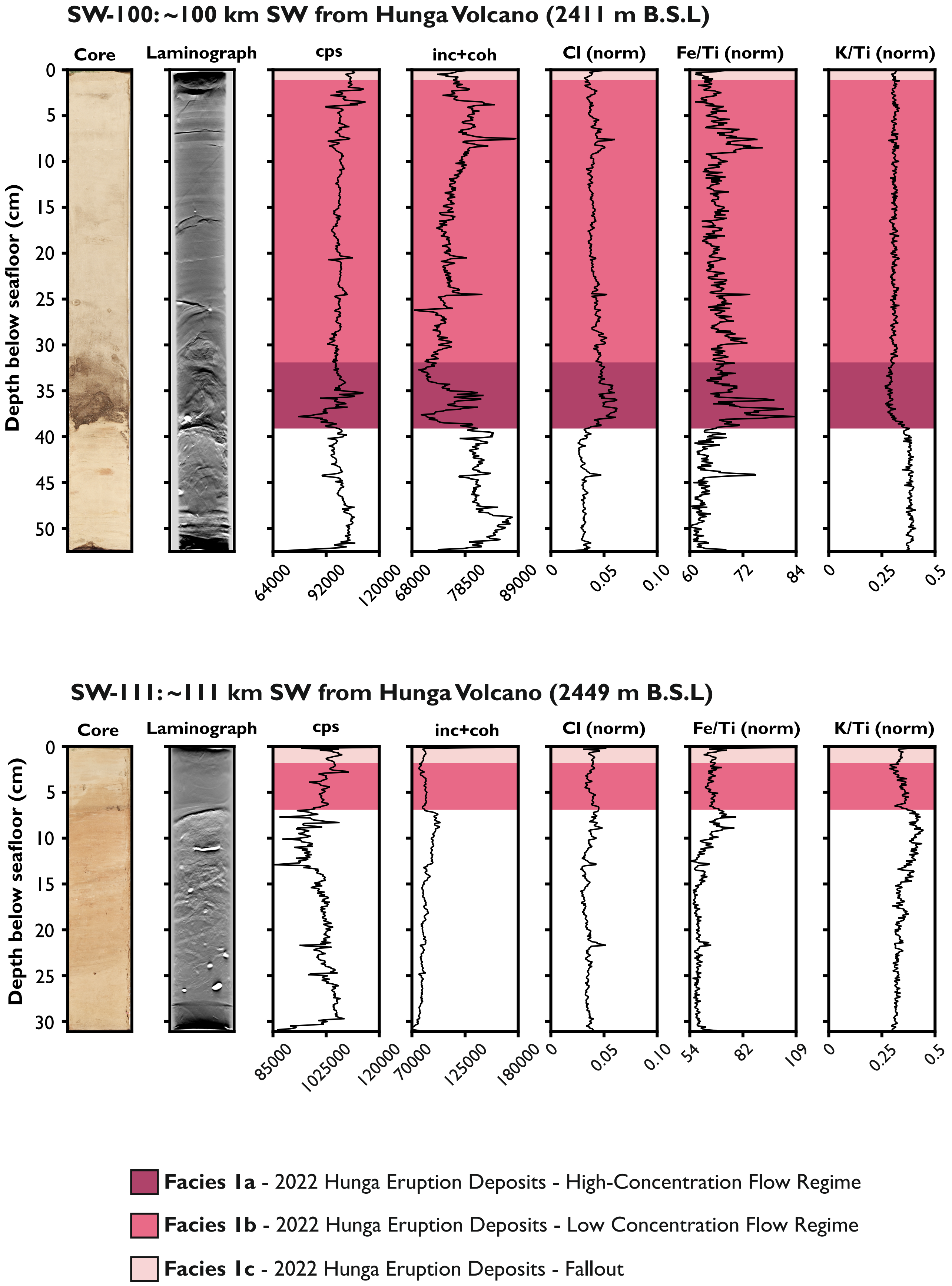

Supplementary Figure 5 Continued

Core Name	Recovery Station	Recovery Site	Core ID	Latitude	Longitude	Distance from Hunga Caldera (km)	Core Recovery (cm)	Deposit Thickness (cm)	Water Depth (m)
**SE – 19**	22	16	STN22-SITE16	-20.7047	-175.2922	19.314	42.5	>42.5	1555
**SW – 20**	31	20	STN31-SITE20	-20.6763	-175.5322	19.982	33.8	>33.8	1718
**NW – 26**	65	30	STN65-SITE30	-20.3917	-175.572	26.377	32.2	>32.2	2068
**SE – 31**	70	17	STN70-SITE17	-20.8123	-175.267	31.096	11.2	>11.2	1376
**SW – 33**	87	21	STN87-SITE21	-20.7338	-175.6398	32.7	18.5	>18.5	1860
**SE – 45**	76	18	STN76-SITE17	-20.916	-175.1985	44.527	25.4	2.45	725
**SSW – 48**	83	04	STN83-SITE04	-20.9177	-175.6427	48.201	22	>22	2000
**SW – 64***	36	22	STN36-SITE22	-20.8772	-175.8988	63.886	62	38	2330
**SW – 80***	90	23	STN90-SITE23	-20.9525	-176.0388	80.620	69.5	30.5	2412
**SW – 100***	96	31	STN96-SITE31	-21.1357	-176.131	100.369	61.5	48.5	2411
**SW – 111***	95	24	STN95-SITE24	-21.0385	-176.323	110.883	31.4	6.5	2449

Table S1 – Summary of sediment cores collected during the TESMaP TAN2206 Research Cruise. Samples recovered using the Ocean Instruments MC-800 multicorer systems. Core recovery location, water depth, and recovered core thickness are detailed. Core name is a combination of the direction (e.g., SE) and distance in km (e.g. 20) from the centre of the Hunga volcano caldera the core was recovered. The thickness of the density current deposit attributed to the 2022 Hunga eruption in the sediment cores is noted. For sediment cores where the base of the deposit was unrecovered thickness is given as > X cm. Sediment cores comprising the SW transect detailed in this report are noted with an asterisk (*).

Sample #	Core Name	Core Depth (cm)	Material	Grains Counted (n)	Massive Glass (n)	Crystal-Rich Glass (n)	Crystals (n)	Pumice (n)	Lithics (n)	Organic (n)	Massive Glass (%)	Crystal-rich Glass (%)	Crystals (%)	Pumice (%)	Lithics (%)	Bio/Fora (%)
**1**	SW-64	10-11	2022 Deposit	308	263	16	4	8	16	1	85.4	5.2	1.3	2.6	5.2	0.3
**2**	SW-64	22 - 23	2022 Deposit	300	202	37	13	17	28	3	67.3	12.3	4.3	5.7	9.3	1.0
**3**	SW-64	28 - 29	2022 Deposit	315	193	65	20	12	25	0	61.3	20.6	6.3	3.8	7.9	0.0
**4**	SW-64	34 - 35	2022 Deposit	305	168	71	23	19	23	1	55.1	23.3	7.5	6.2	7.5	0.3
**5**	SW-64	41 - 42	Pre-2022	300	100	34	9	16	21	120	33.3	11.3	3.0	5.3	7.0	40.0

Supplementary Table 2. Componentry of sediment core SW-64

The electron microprobe analysis, grainsize, Itrax µX-ray Fluorescence (XRF), and componentry data generated in this study are provided within an accompanying source data excel file.

Supplementary References

1. Mackay, K. et al. Environmental Impacts of the 2022 Eruption of Hunga Tonga - Hunga Ha'apai: Voyage Report of Part 1 of the TesMAP Survey of the Region in April-May 2022 (TAN2206). (National Institute of Water & Atmospheric Research Ltd, Wellington, 2022).

2. Mackay, K. Digital Elevation Models of Hunga Volcano; pre- and post- 15 January 2022 eruption. https://doi.org/10.5281/zenodo.7456323 (2022) doi:10.5281/zenodo.7456323.

3. Seabrook, S. et al. Volcaniclastic density currents explain widespread and diverse seafloor impacts of the 2022 Hunga Volcano eruption. Nature Communications 14, 7881 (2023).

4. Clare, M. et al. Fast and destructive density currents created by ocean-entering volcanic eruptions. Science (New York, N.Y.) 381, 1085–1092 (2023).

5. Ryan, W. B. F. et al. Global Multi-Resolution Topography synthesis. Geochemisty, Geophysics, Geosystems 10, 2008GC002332 (2009).

6. Brenna, M. et al. Post-caldera volcanism reveals shallow priming of an intra-ocean arc andesitic caldera: Hunga volcano, Tonga, SW Pacific. Lithos 412–413, 1–21 (2022).
