# Supplementary material for: Far-reaching volcaniclastic density current deposits as evidence of explosive marine eruptions
Source: Nat Commun. 2026 Apr 30;17:5919. doi: 10.1038/s41467-026-71658-8 (PMC13338445; doi:10.1038/s41467-026-71658-8)
Supplement: Supplementary file 2 — Transparent Peer Review file [file 41467_2026_71658_MOESM2_ESM.pdf]

nature portfolio

Peer Review File

Far-reaching volcaniclastic density current deposits as evidence of explosive marine eruptions

Corresponding Author: Mr Jacob Nash

This file contains all reviewer reports in order by version, followed by all author rebuttals in order by version.

Version 0:

Reviewer comments:

Reviewer #1

(Remarks to the Author)
This manuscript discusses volcanic particle dispersal to form seabed deposits drawing on observations from the large 2022 Hunga Tonga–Hunga Haʻapai (HTHH) eruption. It focuses on submarine pyroclastic density currents and potential secondary debris flows as tephra distribution methods, which do seem to make some impressive deposits. While the study seems well-executed and could be a welcome addition to the still-comparatively-small submarine volcano literature, and because the 2022 event provides a scientifically valuable opportunity to study a large event in this setting, I am having difficulty getting fully behind this manuscript formulation at this time for several key reasons I explain below. While there are multiple potential revision pathways, the current case-study formulation is not a good match for the broad-audience manuscript format of Nature Communications, nor does the presentation fully deliver on the topical breadth implied by the title.

1. Topical breadth:
While the manuscript title suggests a broad integrative study that draws on extensive literature and compares multiple volcanoes and volcanic styles and settings to formulate new generalizations beyond the current state of knowledge, it does not achieve that goal. As titled, one might expect a broad synthesis drawing extensively on literature and comparing behaviors across multiple sites to derive general conclusions beyond what is currently established. Yet the actual manuscript has a much narrower focus. The construction and framing of the text currently give a misleading impression of its scope or importance
Currently, the work functions primarily as a case study of debris flows and their associated deposits from a single volcanic eruption, which would be suitable for a specialist journal such as Bull. Volc., or Marine Geology. And I do believe a paper that leans into this narrower focus would have value, especially for specialists. But the authors don't do enough to generalize these observations to meet reader expectations of this current venue in my opinion.

2. Referencing:
Related to breadth, the limited referencing focused on a small number of mostly terrestrial analogs falls short of establishing how these observations fit into the diversity of phenomena associated with pyroclastic and hydroclastic processes beneath the sea (e.g., "explosive" submarine volcanism)
By failing to adequately compare and contrast with an extant literature they largely ignore, including nearby examples that could provide important comparisons (notably on historical "explosive" volcanism and deposits and Havre, West Mata, and Monowa volcanoes, for example, as well as farther afield volcanoes such as i Kick 'em Jenny.Even important previously published observations from the same eruption at the same volcano, are also not incorporated such as the recent Barone et al., 2023 GRL paper showing that Fall deposits are also an important part of this overall eruption story.
I hesitate to mention it, but the current lax approach to placing these results within the broader literature seems designed to overemphasize the supposed uniqueness of the insights presented, a framing that does not come across as particularly scholarly, or collegial. The literature review is especially limited. It draws on a small set of terrestrial or littoral analogs and "old timey" literature, and yet omittis a substantial body of work from other "explosive" (a dI use that term broadly) submarine volcanoes.

3. Volcanological terminology
This is used in a way that feels overly broad and imprecise. For example, the term explosive encompasses a wide range of fragmentation and dispersal conditions, from conduit and vent processes to column dynamics and surface interactions.

Greater precision in defining and applying such terms would help clarify the processes the authors intend to emphasize and better link them to relevant mechanisms.
Given how few papers there are on the topic, the authors could define and apply volcanological terms more precisely, especially those relating to fragmentation and dispersal processes.
The term “explosive”, for instance, is applied in a generic way without clarifying which aspects of fragmentation and dispersal processes are relevant in this case and different or like other notable cases. Plus, the loose application of terrestrial analogue terms to underwater phenomena involving volcanic product dispersal (both lavas and clastics) tends to under-emphasize the importance of key differences between the easier to observe terrestrial settings, and more difficult but volumetrically important submarine cases. Processes that arise from conduit and vent dynamics and eruption column dynamics as they operate under air, water, or ice, all lead to tephra and tephra deposits, each of which have different implications for interpreting the deposits, are essentially left assumed here. It has even recently been shown that deep submarine volcanism leading to substantial clastic aprons at nearby West Mata volcano result from implosive, rather than explosive volcanism (Murch et al.2022 and references therein). This is not to say that some or even most phenomena in the 2022 HTTH eruption were not explosive, but instead used to point out that the term “explosive” is to vague when being used to presume that all tephra was produced in explosive fashion, and too terrestrial centric to be used in the generic way that is being done here.

One can also make the case that by forcing subaqueous primary and secondary volcaniclastic sequence description into the terrestrial analogue naming convention the manuscript is losing detail about differences. One should be able to compare and contrast to illuminate the range of operating processes. Of course, wider recognition of literature would help a lot here too,

Summary
Given these points, the current framing raises concerns about fit for this publication venue. A more accurate title like “An Example of Submarine Pyroclastic Density Currents: Observations and Inferences from the HTHH 2022 Eruption” would better reflect the work’s current scope, though it and also highlight its relatively limited appeal.
To strengthen the manuscript for a wide audience venue such as this, I suggest:

++ Broadening literature engagement, particularly with submarine volcanic deposits from both similar and contrasting settings.

++ Incorporating relevant prior observations from the same eruption.

++ Define key volcanological terms with more precision.

++ Draw out the importance of this study for sea bed science more generally

With a clearer framing, richer literature engagement, greater terminological precision, and a non-specialist angle to the important consequences and implications of these observations, this manuscript could better meet the expectations for publication in this journal.

Reviewer #2

(Remarks to the Author)
I have finally got the chance to go through the paper by Nash et al. on submarine geological record of Hunga volcano, which erupted heavily in 2022, and have found it very interesting. In particular, I have enjoyed reading it, as the paper is well structured and innovative, and timely too. My only major concern is about some absence in recalling the methodology in the main body of the text. I say this because looking at the main parts of the paper it just seems a field work paper, which is OK, but I would highlight more the bathymetric survey also in the main body too; the methods adopted for this work are essential to get to the results, etc... In terms of interpretation, I agree that longer flow runouts may depend of eruptive mechanisms, which is something enounced clearly (and from the field deposits) in
https://onlinelibrary.wiley.com/doi/epdf/10.1111/ter.12561, which will strenghten interpretation.
Best regards

Reviewer #3

(Remarks to the Author)
This paper is fluently written and well structured, and it addresses a topic of actual and wide interest in the volcanological community. It adds to the growing body of research publications on the large Hunga 2022 eruption the investigation of sediment cores collected around and away from the Hunga caldera. The focus is on the investigation of four cores representing a medial to distal radial profile to the SW through the syneruptive volcaniclastic turbidite facies.

I suggest to include Hunga 2022 in the title because the strong point of this paper is actually what it can contribute to the understanding of that eruption. This is not a review of seafloor deposits of submarine eruptions in general, as the title suggests. The last paragraph aims a bit in that direction but I will comment on that further below.

The interpretation that the turbidity current is fed by jetted and collapsed material from the eruption column is, of course, an important result that will also be valuable for future work elsewhere.

Line 45: “shallow” should be quantified here, indicate the actual water depths/range.

Line 66: the main focus is on the four cores named but the other (more proximal) cores are shown with their data in the supplement. Although most of these do not penetrate the entire deposit they still provide valuable information, e.g. on where the stratified coarse facies occurs, where the upper fine facies is thin or thick, … so I don’t quite understand why this information is not exploited in the text.

By the way, in the supplement Fig. 5 the cores SE-45 and SW-33 have been mixed up with respect to Fig. 4.

In the description and discussion I find it less helpful that the entire deposit is called unit 1, unit 2 being the pre-eruption sediment. Earlier work of Seabrook et al. (2023), partly involving the same authors, has already already characterized unit 2 as pelagic clay sediment underlain by older volcaniclastic sediment; this could briefly be mentioned here.
I then suggest to divide unit 1 into three subunits, say 1A, 1B, 1C, with appropriate labels in Figs. 1-3, to then interpret these three parts as done in the text. 1A would then become the deposit of the high-concentration flow regime, 1B the low concentration flow regime, and 1C the final fallout over some months. For example, Seabrook et al. identify the uppermost 10 cm of core SW-80 as the late ash fall, similar to the interpretation of push cores in Chaknova et al. (2025; also involving some of the present authors). This is not recognizable in the present presentation where it is difficult to identify in the figures the boundaries mentioned in the text. If this is done because the authors interpret the top part (of core 80 and others) differently, then this must be discussed.

The use of “high-concentration” should perhaps be discussed a bit more. While it the textural and structural changes upward clearly reflect a decrease in the current’s sediment concentration, the concentration near base was not so high that formation of tractional bed-load processes was suppressed.
When discussing a possibly pulsed turbidity current more specific reference to the repetitions particularly in core 80 and 100 should be made. For example, the graphic interpretation of core 80 in Fig. 1 suggests 6 pulses but the upward grain size decrease in Fig. 3 appears to be fairly steady. What is the origin of the convolute bedding in core 100? Can this be enhanced by topography?

The ”waning flow” interpretation should make use of the ripple images in suppl. Fig. 1 (otherwise, what are they good for??)

Line 147: replace “material” by “magma” because I assume that DRE has been calculated as lithic-free as usual.
Line 162: …. trigger pulses of density ….
Lines 168 ff. would be one place where reference to the proximal core informations from the supplement could be helpful in moving this section a bit closer to observation rather than speculation….

Line 182: … caused currents to ….
Line 190: delete “as”

To be honest, I do not like the section lines 195-240 very much. The first paragraph up to line 204 summarizes the results for this Hunga case. This would be a good place to deepen previous discussion, for example by addressing the question of the different influences on the fining upward and with distance such as progressive loss of coarser particles from the flow, waning eruption, or the distally fining fallout from the plume (Fig. 4). Or are the “pulses” seen in cores 80 and 100 (but also 48, 33 in suppl) related to unsteady current formation, effects of topography on current, or unsteady eruption? I think such kind of discussions could deepen the impact of the paper.

On the other hand, I find comparison with Montserrat not helpful. This is not a typical or representative case for pyroclastic flows entering the ocean globally, it is a small dome eruption 50 times smaller in transported mass than the Honga case. There are other cases of much more voluminous pyroclastic flows that entered the sea radially and produced approximately radial distribution of turbidity currents (e.g., around Gran Canaria). Comparison of Honga with these deposits would yield totally different results. Similarly with the comparison with flank failures. Naturally these do not occur radially although their turbidity currents can spread sectorially. The spreading of primary volcaniclastic turbidity currents as at Honga depends on the morphology of the volcano and the surrounding seafloor and need not always be radial. So the major difference to flank failure is the heterogeneity of the volcaniclastic particles, which is not a new recognition.

Thus my advice would be to skip these comparisons and rather focus on a more in-depth interpretation of the Honga deposits.

Fig. 1: B,C: replace outlined font by solid font. I found the outlined font very hard to read
C: the bathymetric work and interpretation was originally done in Seabrook et al. (2023) and this should be cited here. In fact, the related elaborate method section (line 413 ff..) suggests that this is original work by the present authors which is apparently not the case.
D: insert subunit labels as I suggest in the text…

Report water depth below the core label at each profile (the same in suppl. Fig. 4)

In all profile figure replace Depth by “Depth below seafloor” or “Depth in core”, in figures 1-3 as well as in the supplement figure 4 and 5 (suppl Fig. 5 has no vertical axis labels at all)

Fig. 2: what is the advantage of showing IMG and BRIGHT core images? Why not show each core image at the exposure that shows best the features which should be seen???
(it is not a true color presentation anyway…)

Fig. 3: avoid outlined font but, most of all, correct the scale of 500 centimeter ;-)

Supplement:
Check core 33 and 45 mixed up between Figs. 4 and 5
Fig. 3 caption has a reference 15 to a citation in the main text but to 1 and 2 in the supplement reference list. Either always refer to references as numbered in the main text, or refer to a supplement reference list for all citations.

Reference list:
I am not sure about the rules for Nature Communications but in my experience other journals expect the full DOI link to be provided for each reference.

Reference 32: Cartigny et al. is (2013)

Version 1:

Reviewer comments:

Reviewer #1

(Remarks to the Author)
Manuscript#: NCOMMS-25-43051A Reviewer: Professor Kenna Harmony Rubin
Corresponding Author: Jacob Alexander Nash Due Date: 25-Nov- 25
Title: Voluminous, far-reaching, and multidirectional volcaniclastic density current deposits are key records of explosive marine eruptions

comments for authors
This is a resubmitted manuscript on pyroclastic density currents (PDCs) from the 2022 Hunga Tonga–Hunga Ha'apai (HTHH) eruption. I served as a referee on the previous version. The authors have revised the manuscript substantially in response to comments from both reviewers and have provided a point-by-point rebuttal. The new title more appropriately reflect the content.

My comments fall into two general categories:
(a) those concerning the technical details and conclusions of the study, for the authors, and (b) those regarding the fit of the topic and manuscript, as currently constructed, for this particular journal, for the editors.

1. Technical Assessment
Overall, the authors have significantly improved the manuscript. The eruption is now better contextualized, and the description of the deposits, including their distribution, lithology, and componentry, is clearer and more detailed. As a specialist, I found these sections particularly valuable. However, I am less certain that this level of detail maintains a smooth narrative flow for a more generalist readership and better be found in a supplement.

The manuscript's primary conclusion, that voluminous PDC deposits are present at HTHH, is convincingly documented. The addition of the table summarizing similar events elsewhere is helpful. While not comprehensive, it adequately demonstrates that long-runout PDCs are not unique to this eruption and have been recognized at other submarine or mixed submarine–subaerial systems.

That said, I am not entirely persuaded by the implication that long-runout PDCs are a characteristic or diagnostic feature of energetic submarine fragmental eruptions in general. The evidence here supports the presence of such deposits at HTHH, but the broader generalization remains somewhat speculative.

2. The Missing Context of Fall Deposits
One important aspect remains insufficiently addressed, despite being raised in my previous review: the role, volume, stratigraphic significance, and broader implications of fall deposits in this eruption. Their omission in the prior form of the manuscript, titled "Title: The seafloor record of explosive submarine volcanic eruptions" was inexplicable, and yet even with the narrower retitled focus, I am still at a loss as to why they are not discussed.

In their response to reviewers (specifically comment #4), the authors cite my prior comment, but do not address its substance. This is a missed opportunity for contextualizing the PDCs within the full eruptive sequence.

The stratigraphic columns in the MS clearly show that Unit 1b (PDC deposits) constitutes the majority of the volume, but Unit 1c (fall deposits) are also substantial (approximately ~20% by my visual estimate). This raises several key questions:
• What is the volumetric and spatial relationship between fall deposits and PDC deposits?
• Is this ratio diagnostic of the eruption style or specific to this geologic setting?
• Do fall deposits strictly postdate the PDCs, or might they be cryptically interbedded?

• How do thinning, fining, and dispersion patterns in the fall deposits compare with expectations given the dynamics of plume dispersal, wind, and ocean currents?

Although not the central focus of the manuscript, the fall deposits remain an essential component of the eruptive record and tephra-dispersal system, and of other submarine volcanoes generally. Their omission here risks implying that long-runout PDCs are the sole mechanism of regional tephra transport.

Relevant published work underscores Fall deposit significance. Barone et al. (2022) – a paper I referred the authors to previously - document satellite-observed ocean-surface changes (opacity, color, temperature, chlorophyll, plankton count) attributable to ashfall from the eruption, implying a minimum regional ash thickness of ~20–197 mm. Kelly et al. (2024) estimate a minimum fall-deposit volume of ~1.8 km^3 (out of >6.3 km^3 total erupted volume). This proportion is consistent with the stratigraphic estimate I made based on data in the current manuscript.

Given this, the manuscript could at least briefly:
• Describe the fall deposits and cite the relevant studies,
• Note their position(s) in the eruptive sequence (Barone et al. report two separate fall events),
• Discuss the partitioning of tephra between PDCs and fallout, and
• Comment on whether this partitioning is typical of submarine, subaerial, or hybrid eruptions.
Such additions would provide a more complete picture of the eruption and better situate the PDC deposits within the broader volcanological context. They could also highlight useful avenues for future work.

References:
Barone et al. (2022), Geophysical Research Letters, 49(17), e2022GL099293.
Kelly et al. (2024), Bulletin of Volcanology, 86:59.

Reviewer #3

(Remarks to the Author)
My (and other reviewers‘) comments appear to have been adequately considered in the revised manuscript. Particularly the discussion section about comparison between various modes of volcaniclastic turbidity current formation and their deposits has been greatly improved. I do, however, still have some minor comments.
(1) Line 249: another important factor for long runout is fine grain size, i.e. intense fragmentation in/near vent.
(2) A brief description of the pre-eruption unit 2 sediments should be included on page 3. This becomes relevant when entrainment of such sediment is invoked as a means to extend the runout distance of the currents (lines 256 ff). The volume of ~3.5 km3 of entrained sediment (line 257) needs to be explained and a reference added (presumably no. 15 ??). Can this sediment be distinguished from the newly erupted material, and if so, was it recognized in the cores?
(3) Reference 24 is used several times in the context of seismic signals supporting the present model of initiation and motion of the turbidity currents. However, this information is not accessible (in pre-print but no information where it might eventually be found). Is there a way to briefly show relevant seismic evidence in this paper?
(4) This may be important additional evidence because I still find the identification of the “stacked fining-up sequences” a bit weak. Not that I doubt the unsteady nature of material delivery during the eruption but how this is deduced from the deposits is not so easy to follow while this is used as a major distinguishing criterion in the last discussion section. In distal cores it is not seen because of the overall finer grain size, in the most proximal core 64 the laminograph (Fig. 2) shows change from upper plane bed to ripple structure while grain size indicates possible three cycles (Fig. 3). In core 80 there is visual evidence of repeated darker layers (Fig. 2) but no real grain-size evidence (Fig. 3). So I think it would be worth while to more clearly present the evidence for (at least 6) stacked cycles.
(5) Color coding in the XRF profiles Fig. 2: Unit 1a is used here as Facies 1a, a unit has a fixed stratigraphic position while a facies can occur repeatedly. Should not be confused. Fig. 2 has nice light core photographs with well-visible structures while those in Fig. 3 are too dark! Lighten up!
(6) Lines 295 ff: distinguishing “continuous, pulsed” (Honga) from “single large volume PDC) appears a bit artificial. This can be made for the comparison with some deposits but not with others. Where large PDCs entered the ocean, ignimbrite on land mostly shows several flow units, i.e. “pulse-like” emplacement rather than “at once”. Consequently submarine turbidites from such events can well contain fining-up cycles (e.g., Schindlbeck et al. 2013, Kutterolf et al. 2014, Freundt & Schmincke 1998). One aspect that is not discussed here is that submarine tuffs from large-magnitude PDC eruptions can show vertical density grading of clasts.
(7) Line 298: Miocene Gran Canaria had radial distribution of PDCs into the ocean and this is not a particularly small island. The more important factor would be volume flux versus depth of topography, I guess.

Version 2:

Reviewer comments:

Reviewer #1

(Remarks to the Author)
I have had the opportunity to reread the revised manuscript and the authors’ extensive documentation addressing the editorial changes in this version. The manuscript resolves all my previous concerns. The manuscript is now clearly written,

methodologically sound, and strikes an appropriate balance between specialist detail and broader accessibility. As I said in prior reviews, the topic is inherently engaging. I feel that this is an important study, one that forms what will likely be a widely read contribution to the journal. I therefore recommend publication in its present form.

I also wish to note that the authors' discussion of fall deposits and the admixture of lithic fragments, including those of biogenic origin, together with the settling-velocity arguments, provides a compelling and well-supported explanation for why these materials were not a primary focus of the paper. I also accept the nuances they have added about sea-surface crossing versus entirely submarine PDCs

Upon reflection, my earlier comments about fall deposits may have been too influenced by my own observations from other volcanic systems. In particular, I believe there is an important distinction between fall deposits derived from eruption columns that breach the air–sea interface and those that do not. The latter may be significant at other volcanoes, especially proximal to the volcanic vent, but they do not appear to have been central to this specific eruption, especially at vent distal locales.

I also feel that the discussion of observations elsewhere has improved and makes he case that this is a common phenomenon. Yet, since most of the examples given are for large eruptions, I would be tempted in the body of the paper to recognize in a sentence that not all observations from this event will necessarily be transferable to smaller eruptions.,, and then perhaps even speculate that the proportion of PDC to Fall deposition may be nonlinear with mass discharge rate.

If I were to offer one other minor suggestion to further strengthen an already excellent write-up, it would be to emphasize in the abstract, perhaps even in the very first sentence, that this event represents the largest observed eruption on Earth this century. Its marine setting and exceptional level of documentation provide unprecedented clarity into the behavior of such large-scale systems.

To summarize, overall, this is a strong and impactful contribution, and I fully support its publication.

-Kenna Rubin

Reviewer #3

(Remarks to the Author)
My comments on the first revision have now been adequately addressed in the second revision and I have nothing more to add.

Open Access This Peer Review File is licensed under a Creative Commons Attribution 4.0 International License, which permits use, sharing, adaptation, distribution and reproduction in any medium or format, as long as you give appropriate credit to the original author(s) and the source, provide a link to the Creative Commons license, and indicate if changes were made.
In cases where reviewers are anonymous, credit should be given to 'Anonymous Referee' and the source.
The images or other third party material in this Peer Review File are included in the article's Creative Commons license, unless indicated otherwise in a credit line to the material. If material is not included in the article's Creative Commons license and your intended use is not permitted by statutory regulation or exceeds the permitted use, you will need to obtain

permission directly from the copyright holder.
To view a copy of this license, visit https://creativecommons.org/licenses/by/4.0/

Overview

This is the response to reviewers for Manuscript *NCOMMS-25-43051-A*.

All line references in the following document correspond to the version of the manuscript called "*RevisedManuscript_NCOMMS-25-43051-A*" with tracked changes enabled (Simple Markup view in Microsoft word).

We sincerely appreciate and thank the reviewers for their evaluation of our manuscript and their detailed and constructive comments. Their suggestions have contributed substantially to improving the quality, clarity, and precision of the manuscript.

Major manuscript changes

The main manuscript changes include:

(1) Title Change – "*Voluminous, far-reaching and multidirectional density current deposits are key records of explosive marine eruptions*"

(2) A significantly revised discussion and more comprehensive literature analysis. We expand the number of case-studies incorporated in our review of distinguishing density-current deposits formed by differing volcanic processes. Specifically, we introduce and discuss different syn-eruptive density current forming processes at shallow and deep-submarine volcanoes.

(3) Addition of *Figure 5* to the discussion to illustrate the different volcanic processes that trigger underwater volcaniclastic density currents.

Editorial Changes:

Title – We have revised the title to one that befits the focus and scope of the manuscript.

- Previous title: "*The seafloor record of submarine eruptions*"
- New title: "*Voluminous, far-reaching and multidirectional density current deposits are key records of explosive marine eruptions"*

Authorship

- No changes to authorship
- Minor changes to author names
- Two institutional changes:
  - "*[1]OceanBioGeoscience, National Oceanography Centre (NOC), Southampton, UK*" to *"[1]Volcanology & Geohazards Laboratory, National Oceanography Centre (NOC), Southampton, UK"*
  - *"[3]National Institute of Water and Atmospheric Research (NIWA), Auckland, New Zealand"* to *"[3]Earth Sciences New Zealand, New Zealand"*

Abstract

- Minor changes to sentence structure
- Shortening of abstract to adhere to 150-word limit

Introduction

- Added definition of an "*Underwater Volcaniclastic Density Current.*"
- Re-characterizing/re-naming flank collapse events to mass-wasting events (which incorporates more localised events such as caldera wall, lava flow front, or dome collapses).
- Minor sentence reconstruction to improve clarify and focus.

Results & Discussion

The 2022 eruption of Hunga Volcano

This section was expanded to incorporate:

- Extended and detailed description of the Hunga volcano edifice including depth and caldera geometry.
- Extended background on historic eruptions at Hunga volcano.
- Introduced the likely triggers of the January 15th, 2022, eruption.
- Referenced the likely fragmentation depth range.
- Additional references when discussing the eruption column height and composition.

Complex flow regimes revealed from seafloor deposits

Minor changes were made to this section:

- Separation of Unit 1 (2022 eruption deposits) into three subunits, 1a, 1b, and 1c, to improve 2022 eruption deposit description and clarity.
- Direct reference to the number of black sand layers present in the deep-sea cores.
- Added a definition of a "*Turbidity Current.*"
- Specific clarification that the 2022 Hunga eruption deposit was emplaced by a high-density turbidity current.
- Added a definition of a *"High-density turbidity current"* and how it differs from a debris flow.
- Addition of depth values for the ash fallout subunit (1c).
- Included sentence on disturbed planar bedding and likely causes (including references).

Spatial evolution of underwater density currents

Minor changes made to incorporate separation of the 2022 eruption deposit into subunits (1a-1c) within the text. A sentence was added to highlight the surficial deposit ripples as evidence of a distally waning flow:

- Lines 129-130: *Moving away from the volcanic edifice deposits surface ripples decrease in size before disappearing in the most distal locations (Figure S1).*

Rapid entry of voluminous material fuels long-runout currents

- Firstly, the subtitle was changed from '*Vertical entry of volcanic material as a trigger of multidirectional and long-runout volcaniclastic turbidity currents*' to '*Rapid entry of voluminous material fuels long-runout currents.*' This was done to achieve the 60-character limit for subtitles.
- Minor changes to improve clarity and grammar.
- Extended the section discussing flow transformation to include that non-identical case-studies are required to interpret the Hunga 2022 eruption deposits as there are no directly comparable examples.
- Extended the section discussing the radial dispersion of the 2022 Hunga eruption deposits to specifically highlight both submarine and subaerial examples of eruption column collapse triggering the multidirectional dispersion of volcaniclastic density currents.
- A paragraph discussing the record of variable and waning mass-eruption rate (MER) record within the 2022 Hunga eruption deposits was moved from the next section to the end of this section.

<u>Eruption records in underwater density current deposits</u>

This section was substantially revised and restructured:

- Firstly, the title was changed from '*submarine deposits as an eruption record'* to '*Eruption records in underwater density current deposits"* to mark the change in scope of this section and to adhere to the 60-character limit for subtitles.
- A paragraph talking about the record of mass-eruption rate in the 2022 eruption deposits was moved into the previous section **(Lines 217-223).**
- We expand on the 2022 Hunga eruption to emphasize that is it more than a case study of a single eruption that triggered underwater density currents but the most comprehensive case-study of an explosive volcanic process and the subsequent utility of the eruption deposits in identifying similar volcanic settings.
- We have significantly modified Table 1 (See section below called Table 1) to include 16 case studies of underwater volcaniclastic density currents triggered by different volcanic processes.
- Major restructuring of this section to shift the focus to the various mechanistic processes that produce underwater density-currents rather than comparisons with two case studies (2003 dome collapse at Soufriere Hills and the 150-170 ka Icod Landslide).
- Sections comparing the Hunga 2022 eruption deposits with (i) water-entering pyroclastic density currents, (ii) submarine eruptions, and (iii) mass-wasting events are expanded to incorporate additional comparable case-studies.

- The submarine-eruption section is significantly expanded to included comparisons to surtseyan eruptions, shallow-submarine explosive eruptions, and deep-submarine explosive eruptions.
- Addition of Figure 5 to illustrate the different processes that can trigger underwater volcaniclastic density currents at marine volcanic settings.

Conclusions

- Minor changes and additions to sentence structure.
- Section added at the end of the conclusion that underlines the record of changing eruption processes and eruption evolution (e.g., mass eruption rate variability) within the submarine Hunga eruption deposits:
    - **Lines 341-343:** *Beyond density current behaviour, the 2022 Hunga submarine eruption deposits preserve a record that correlates with observed mass eruption rate changes. The long-term eruption record held by these submarine deposits are a tool to unravel past eruption dynamics.*

Methods

- Minor additions and restructuring of sentences to improve clarity.
- Small re-write and re-arrangement of the section "*Bathymetric Surveys"* to make it clear that the elevation difference map DEM was created by Seabrook et al., (2023) and Clare & Yeo et al., (2023).
- Shortening and refining of the section "*Sediment Core Collection and Non-Destructive Analysis"* to improve clarity.

Figures and Tables

<u>Figure 1</u>

- **1A-1C** – Outlined text was removed. Instead, a semi-transparent background was added behind text to provide clarity
- **1D**
    - Depth below sea level values were added beneath core names.
    - Y-axis label was changed from "*Depth (cm)*" to "*Depth below seafloor (cm)*."
    - Green background identifying the 2022 eruption deposits replaced with three separate colours identifying the eruption deposit subunits 1a – dark pink, 1b -pink, 1c – light pink.
    - Unit 2 colour changed from a dark brown-orange to a lighter orange.
    - Figure legend modified to incorporate subunits 1a, 1b, and 1c.
    - Sections highlighting the recurrence of subunit 1a up-core are highlighted by dark pink bands. Annotations with arrows and question marks indicate that the lateral extent of these layers is unknown.

Figure 2

- Modification to text, titles, and labels to improve overall figure clarity
- Core image removed – only brightened and laminograph images kept
- Re-arrangement of labels and axis titles to improve clarity on the XRF plots
- Green background identifying the 2022 eruption deposits replaced with three separate colours identifying the eruption deposit subunits 1a – dark pink, 1b -pink, 1c – light pink
- *"Depth (cm)"* replaced with "*Depth below the seafloor (cm)*" on the y-axis.

Figure 3

- Outlined text on the SEM images removed and replaced with plain black text with a transparent white background to improve eligibility
- Error of "*cm*" on the scale bar and x-axis of the contour plots rectified and replaced with correct "*μm*" units
- *"Depth (cm)"* replaced with "*Depth below the seafloor (cm)*" on the y-axis of the contour plots

Figure 4

No changes made

Figure 5

New addition. Figure 5 provides illustrations of the volcanic processes that trigger underwater volcaniclastic density currents at marine volcanic settings.

Table 1

Significantly modified Table 1:

- Table was flipped such that eruption and deposit characteristics are represented by columns and specific case studies are the rows
- Additional columns included: Volcano, Core Availability, Repeat Seafloor Surveys, VEI
- Number of case-studies was expanded from three to sixteen
- The following lists the case-studies for each density-current triggering volcanic process:
    - ***Surtseyan eruption*** – 13 Ka Black Point eruption, 18.6 Ka Pahvant Butte eruption.
    - ***Shallow-Submarine eruption*** – Archaeos Tuff forming eruption, NW Rota-1 2012 eruption, Hunga 2022 eruption.
    - ***Deep-Submarine eruption***– 2012 Havre eruption.
    - ***Mass wasting events*** - 150-170 Ka Icod Landslide, 1888 Ritter Island landslide, 1998-2007 sector collapses at Monowai volcano, localised collapses at Havre post-2012 eruption, 2015 Kick em' Jenny eruption.
    - ***Water-entering pyroclastic density currents*** – 1883 Krakatau eruption, 2003 Soufriere Hills dome collapse, 2022-2023 volcanic activity at Stromboli, and the formation of the Miocene Nakanai trough volcanics.

References

34 new references are added. Here we list the new references and group them based on the reason for their addition. The number of each reference is identified by the bracketed number at the beginning of the reference e.g., (12):

Addition to underwater volcaniclastic density current background and previous research:

- (11) Kühn, M. *et al.* Volcanic Flank Collapse, Secondary Sediment Failure and Flow-Transition: Multi-Stage Landslide Emplacement Offshore Montserrat, Lesser Antilles. *Geochemisty, Geophysics, Geosystems* **25**, e2024GC011564 (2024).

- (12) Carey, S. N. & Schneider, J.-L. Volcaniclastic Processes and Deposits in the Deep-Sea. in Developments in Sedimentology vol. 63 457–515 (Elsevier, 2011).

Additions to Hunga volcano history and 2022 eruption processes:

- (17) Colombier, M. et al. Vesiculation and Quenching During Surtseyan Eruptions at Hunga Tonga-Hunga Ha'apai Volcano, Tonga. JGR Solid Earth 123, 3762–3779 (2018).
- (21) Wu, J. et al. Edifice weakening and decompression triggered deep magma rise and the catastrophic 15 January 2022 Hunga eruption. Earth and Planetary Science Letters (2025).
- (23) Carr, J. L., Horváth, Á., Wu, D. L. & Friberg, M. D. Stereo Plume Height and Motion Retrievals for the Record-Setting Hunga Tonga-Hunga Ha'apai Eruption of 15 January 2022. *Geophysical Research Letters* **49**, e2022GL098131 (2022).
- (24) Cronin, S. J. et al. Submarine caldera subsidence creates extreme tsunami hazard. in pre-print (2025).
    - This is an updated version of – (17) Cronin, S. J. et al. Multiproxy evidence for syn-eruptive submarine caldera collapse during the January 2022 Hunga eruption. In pre-print (2025).
- (29) Baron, A. et al. Early Evolution of the Stratospheric Aerosol Plume Following the 2022 Hunga Tonga-Hunga Ha'apai Eruption: Lidar Observations From Reunion (21°S, 55°E). Geophysical Research Letters 50, e2022GL101751 (2023).
- (32) Ribó, M. et al. Time-series insights of the geomorphologic changes of the Hunga submarine caldera. Preprint at https://doi.org/10.21203/rs.3.rs-7736926/v1 (2025).

Additions to turbidity current processes and deposition:

- (37) Meiburg, E. & Kneller, B. Turbidity Currents and Their Deposits. Annu. Rev. Fluid Mech. 42, 135–156 (2010).
- (42) Jutzeler, M. et al. Coring disturbances in IODP piston cores with implications for offshore record of volcanic events and the Missoula megafloods. Geochem. Geophys. Geosyst. 15, 3572–3590 (2014).

- (43) Butler, R. W. H., Eggenhuisen, J. T., Haughton, P. & McCaffrey, W. D. Interpreting syndepositional sediment remobilization and deformation beneath submarine gravity flows; a kinematic boundary layer approach. JGS 173, 46–58 (2016).

- (44) Shanmugam, G. Global case studies of soft-sediment deformation structures (SSDS): Definitions, classifications, advances, origins, and problems. Journal of Palaeogeography 6, 251–320 (2017).

Additions to discussions around density current formation during the 2022 Hunga eruption:

- (54) Kelly, L. J. et al. Airfall volume of the 15 January 2022 eruption of Hunga volcano estimated from ocean color changes. Bull Volcanol 86, 59 (2024).

- (56) Metcalfe, A. et al. Submarine ash megabed fed by far-traveled, shoreline-crossing pyroclastic currents from a large explosive volcanic eruption. Science AdvAnceS (2025).

- (66) Doronzo, D. M., Giordano, G. & Palladino, D. M. Energy facies : A global view of pyroclastic currents from vent to deposit. Terra Nova 34, 1–11 (2022).

Additions to discussions regarding the deposits of underwater volcaniclastic density currents formed by different volcanic processes:

- (67) Casalbore, D. et al. Very fast canyon formation and evolution along active volcanic flanks: A case from Stromboli Island. Geomorphology 488, 109961 (2025).

- (68) Calvari, S. et al. Multi-parametric study of an eruptive phase comprising unrest, major explosions, crater failure, pyroclastic density currents and lava flows: Stromboli volcano, 1 December 2020–30 June 2021. Frontiers in Earth Science 10, 1–24 (2022).

- (69) Giordano, G. & De Astis, G. The summer 2019 basaltic Vulcanian eruptions (paroxysms) of Stromboli. Bull Volcanol 83, 1 (2021).

- (70) Cole, P. D. et al. Explosive sequence of La Soufrière, St Vincent, April 2021: insights into drivers and consequences via eruptive products. SP 539, 81–106 (2024).

- (71) Mandeville, C. W., Carey, S., Sigurdsson, H. & King, J. Paleomagnetic evidence for high-temperature emplacement of the 1883 subaqueous pyroclastic flows from Krakatau

Volcano, Indonesia. Journal of Geophysical Research: Solid Earth 99, 9487–9504 (1994).

- (72) Mandeville, C. W., Carey, S. & Sigurdsson, H. Sedimentology of the Krakatau 1883 Submarine Pyroclastic Deposits. Bulletin of Volcanology 57, 512–529 (1996).

- (74) Calvari, S. et al. Overflows and Pyroclastic Density Currents in March-April 2020 at Stromboli Volcano Detected by Remote Sensing and Seismic Monitoring Data. Remote Sensing 12, 3010 (2020).

- (75) Self, S. & Rampino, M. R. The 1883 eruption of Krakatau. Nature 294, 699–704 (1981).

- (76) Kutterolf, S. et al. Large volume submarine ignimbrites in the Shikoku Basin: An example for explosive volcanism in the Western Pacific during the Late Miocene. Geochem Geophys Geosyst 15, 1837–1851 (2014).

- (85) Carey, R. et al. The largest deep-ocean silicic volcanic eruption of the past century. Sci. Adv. 4, e1701121 (2018).

- (78) Verolino, A., White, J. D. L., Dürig, T. & Cappuccio, F. Black Point – Pyroclasts of a Surtseyan eruption show no change during edifice growth to the surface from 100 m water depth. Journal of Volcanology and Geothermal Research 384, 85–102 (2019).

- (79) Verolino, A., White, J. D. L. & Brenna, M. Eruption dynamics at Pahvant Butte volcano, Utah, western USA: insights from ash-sheet dispersal, grain size, and geochemical data. Bull Volcanol 80, 81 (2018).

- (81) Chadwick, W. W. et al. Direct video and hydrophone observations of submarine explosive eruptions at NW Rota-1 volcano, Mariana arc. J. Geophys. Res. 113, 2007JB005215 (2008).

- (82) Walker, S. L. et al. Eruption-fed particle plumes and volcaniclastic deposits at a submarine volcano: NW Rota-1, Mariana Arc. J. Geophys. Res. 113, 2007JB005441 (2008).

- (86) Murch, A. P., White, J. D. L. & Carey, R. J. Characteristi9cs and Deposit Stratigraphy of Submarine-Erupted Silicic Ash, Havre Volcano, Kermadec Arc, New Zealand. Front. Earth Sci. 7, 1 (2019).

- (87) Dürig, T. et al. Deep-sea eruptions boosted by induced fuel–coolant explosions. Nature Geoscience 13, 498–503 (2020).

- (88) Volcanic Debris Avalanches: From Collapse to Hazard. (Springer International Publishing, Cham, 2021). doi:10.1007/978-3-030-57411-6.

- (89) Allen, R. W. et al. 30 Years in the Life of an Active Submarine Volcano: A Time-Lapse Bathymetry Study of the Kick-’em-Jenny Volcano, Lesser Antilles. Geochem Geophys Geosyst 19, 715–731 (2018).

- (90) Chadwick, W. W. et al. Cyclic eruptions and sector collapses at Monowai submarine volcano, Kermadec arc: 1998–2007. Geochem Geophys Geosyst 9, 2008GC002113 (2008).

- (97) Watt, S. F. L. et al. From catastrophic collapse to multi-phase deposition: Flow transformation, seafloor interaction and triggered eruption following a volcanic-island landslide. Earth and Planetary Science Letters 517, 135–147 (2019).

Acknowledgements

- Removal of C. McGuire and M. Charidemou as they are listed as authors
- Addition of Richard Pearce at the NOC for his support in collecting SEM images

Author contributions

- No changes made

Data and Material Availability

- No changes made

Competing interests

No changes made

Supporting Information

Supplementary Figure 1

- Was previously Supplementary Figure 4 but following revisions was reference earlier in the main manuscript text.
- Green background identifying the 2022 eruption deposits replaced with three separate colours identifying the eruption deposit subunits 1a – dark pink, 1b -pink, 1c – light pink.
- Figure legend modified to incorporate subunits 1a, 1b, and 1c.
- Depth below sea level added below core name and location.
- Y-axis label changed from ‘*Depth (cm)’* to ‘*Depth below seafloor (cm).’*

Supplementary Figure 2

- Was previously Supplementary Figure 1.

Supplementary Figure 3

- Was previously Supplementary Figure 2.

Supplementary Figure 4

- Was previously Supplementary Figure 3.

Supplementary Figure 5

- Green background identifying the 2022 eruption deposits replaced with three separate colours identifying the eruption deposit subunits 1a – dark pink, 1b -pink, 1c – light pink.
- Figure legend modified to incorporate subunits 1a, 1b, and 1c.
- Depth below sea level added below core name and location.
- Y-axis label changed from ‘*Depth (cm)’* to ‘*Depth below seafloor (cm).’*

Supplementary Table 1

- No changes made.

Supplementary Table 2

- No changes made.

Point-to-point response

All line references correspond to the updated version of the manuscript

---

Reviewer #1

Comment 01: This manuscript discusses volcanic particle dispersal to form seabed deposits drawing on observations from the large 2022 Hunga Tonga–Hunga Ha'apai (HTHH) eruption. It focuses on submarine pyroclastic density currents and potential secondary debris flows as tephra distribution methods, which do seem to make some impressive deposits. While the study seems well-executed and could be a welcome addition to the still-comparatively-small submarine volcano literature, and because the 2022 event provides a scientifically valuable opportunity to study a large event in this setting, I am having difficulty getting fully behind this manuscript formulation at this time for several key reasons I explain below. While there are multiple potential revision pathways, the current case-study formulation is not a good match for the broad-audience manuscript format of Nature Communications, nor does the presentation fully deliver on the topical breadth implied by the title.

Response 01: Thank you for your review and valuable comments. In the accompanying updated manuscript, we have considered your concerns and suggested revisions and have made changes that aim to better clarify and focus our research aims, findings, and conclusions. We believe this study is valuable to a broad audience, as this eruption is one of only a very few well documented explosive eruptions in the marine environment. The impacts of this study are therefore two-fold, firstly they provide insights into processes that have only recently been recognised but which are relevant to interpreting deposits from explosive marine eruptions globally, secondly, the recurrence rates of explosive marine eruptions are almost universally unknown, yet as demonstrated by the eruption of Hunga Volcano, can have devastating and wide reaching impacts, compounded by the fact that almost none of these volcanoes are actively monitored. Thus, the key to understanding the hazard they pose is interpreting their deposits in terms of the size and styles of eruption, and this contribution provides some of the first, well characterised information on how to distinguish and interpret explosive eruptions like Hunga from other processes, a key knowledge gap in understanding volcanic hazard globally.

Comment 02: Topical breadth: While the manuscript title suggests a broad integrative study that draws on extensive literature and compares multiple volcanoes and volcanic styles and

settings to formulate new generalizations beyond the current state of knowledge, it does not achieve that goal. As titled, one might expect a broad synthesis drawing extensively on literature and comparing behaviours across multiple sites to derive general conclusions beyond what is currently established. Yet the actual manuscript has a much narrower focus. The construction and framing of the text currently give a misleading impression of its scope or importance. Currently, the work functions primarily as a case study of debris flows and their associated deposits from a single volcanic eruption, which would be suitable for a specialist journal such as Bull. Volc., or Marine Geology. And I do believe a paper that leans into this narrower focus would have value, especially for specialists. But the authors don't do enough to generalize these observations to meet reader expectations of this current venue in my opinion.

Response 02: Thank you for raising this point. We agree that the title does not wholly-represent the results or discussions presented in the manuscript. To address this, we have significantly revised the title from '*The Seafloor record of submarine explosive eruptions*' to "*Voluminous, far-reaching and multidirectional density current deposits are key records of explosive marine eruptions"* to better represent the contents of this manuscript.

We have also reframed much of the later discussion present in this manuscript and extended the number of comparative events (although we are limited by what is available and well characterised). The section previously subtitled "*Seafloor deposits as an eruption record*" has be renamed to "*Eruption records in underwater density current deposits"* to better highlight the specific processes and hazards we are comparing.

We have aimed to illustrate that the Hunga eruption deposits are the most comprehensive record of a highly explosive (VEI 6), phreatomagmatic, caldera-forming eruption that triggered the radial dispersion of far-reaching submarine density currents – and specifically, how we might distinguish these deposits from other current forming processes in the historic rock record. The Hunga 2022 eruption is not just a single case-study of an explosive shallow-submarine eruption but the best constrained characterization of an explosive marine volcanic eruption and thus provides a unique opportunity to apply the learnings from this eruption to interpret volcanic histories at other locations. As we mention above, these eruptions are potentially devastating, occurring at volcanoes that are poorly monitored, and yet we have a poor understanding of their recurrence rates. Comparison with other density current forming processes is key to the identification of similar volcanic processes elsewhere (through core and bathymetry comparisons) and will allow better interpretation of marine volcanic records elsewhere that can improve our understanding of global volcanic hazards. However, we do

recognise that it would be useful to include wider comparison of other settings and expand on this below.

Comment 03: ***Referencing***: Related to breadth, the limited referencing focused on a small number of mostly terrestrial analogues falls short of establishing how these observations fit into the diversity of phenomena associated with pyroclastic and hydroclastic processes beneath the sea (e.g., “explosive” submarine volcanism). By failing to adequately compare and contrast with an extant literature they largely ignore, including nearby examples that could provide important comparisons (notably on historical “explosive” volcanism and deposits and Havre, West Mata, and Monowai volcanoes, for example, as well as farther afield volcanoes such as Kick ‘em Jenny.

We agree with the reviewer that the case studies used to interpret both the Hunga eruption deposits and associated parental current behaviour, as well as in distinguishing these deposits from density currents triggered by other mechanisms, could be expanded. We also acknowledge that some of the case-studies used are indeed terrestrial analogues (or at least initially). For example, we include the deposits of the 2003 Soufriere Hills dome collapses and the Pavey Ark ignimbrite, both water-entering pyroclastic density currents, to infer the location of flow transformation during the 2022 Hunga eruption:

- **Lines 201-205:** *Underwater volcaniclastic density currents triggered by lake/ocean entry of hot pyroclastic density currents record deposition of their densest and coarsest material proximally (or in shallow waters) as fines-depleted breccias or lobate deposits, causing elutriation of fine and low-density material, which mixes with ambient water to form a cool and relatively low-concentration turbidity current[8,10].*

We do this, because there is no currently existing field-study that details flow transformation of underwater volcaniclastic density currents at a wholly-submarine or shallow-submarine eruption to compare to Hunga (at least one where there is detailed deposit geomorphology, proximal-to-distal sampling, repeat seafloor surveys). As such, we rely on “terrestrial analogues” and different current-forming mechanisms to infer some of the current behaviours at Hunga volcano. However, we understand that it was not clear in the manuscript as to why non-identical case-studies were being used. As such, we remedy this absence, by including:

- **Lines 197-199:** *Lacking directly comparable field-studies to the 2022 Hunga eruption, we draw on case-studies of eruptions and density-current behaviours that provide valuable analogues for interpreting the 2022 Hunga submarine deposits and density current behaviours.*

Furthermore, we agree that in the discussion the case-studies that were used to represent different density current forming mechanisms are limited. We focused on the 2003 dome collapses at Soufriere Hills volcano because it is one of the only field-studies where there are pre- and post-eruption bathymetric surveys, detailed density current deposit characterization, and direct observations of subaerial-to-submarine flow transformation. Direct observations and well constrained deposits are absent from many other eruptions and volcanic processes. This is also the case for mass-wasting events – the 150-170 Ka Icod landslide was exceptionally well constrained. Whilst looking at every volcanic or eruptive process that deposits volcaniclastic/clastic material on the flanks of a submarine volcano is beyond the scope of this manuscript, we agree that a more robust comparison with other settings would be useful. A wider-range of case-studies that blueprint a broader range of density-current forming mechanisms would help to accurately identify the distinguishing features of the Hunga 2022 eruption deposits. To address this gap, we have increased the number of case-studies discussed and included in Table 1 from three to sixteen. We consider all case-studies presented here to be critical to both distinguishing different mechanistic processes producing underwater density currents but also highlighting the paucity of complete deposit records for many underwater density currents. We believe this expanded comparison includes a large number of relevant studies where there is at least some comparative field data that can be compared to the Hunga deposits.

The newly included case-studies include density currents triggered by:

- *Surtseyan eruptions* – 13 Ka Black Point eruption & 18.6 Ka Pahvant Butte eruption
- *Explosive shallow-submarine eruptions* – Archaeos Tuff forming eruption, NW-Rota 2012 eruption, and the Hunga 2022 eruption
- *Deep-submarine eruptions* - 2012 Havre eruption
- *Mass-wasting events* - 150-170 Ka Icod Landslide, 1888 Ritter Island landslide, 1998-2007 sector collapses at Monowai volcano, localised collapses at Havre post-2012 eruption, 2015 Kick em' Jenny eruption
- *Water-entering pyroclastic density currents* – 1883 Krakatau eruption, 2003 Soufriere Hills dome collapse, 2022-2023 volcanic activity at Stromboli, and the formation of the Miocene Nakanai trough volcanics.

We incorporate these additional case-studies in the discussion to better distinguish the Hunga 2022 density current deposits:

- **Lines 246-330:** *Submarine deposits are often the only record of marine eruptions, their triggers, and other associated volcanic processes. The characterization of the 2022 Hunga Volcano eruption deposits, combined with repeat seafloor bathymetry and seismic records,*

offers the most comprehensive record of exceptionally rapid, syn-eruptive, pulsed, and multidirectional, delivery of volcaniclastic material during an explosive shallow-submarine eruption to date. As such, these deposits can act as a tool for interpreting past comparable processes from stratigraphic records at other volcanic sites. However, the efficacy of such deposit-based interpretation depends upon different volcanic mechanisms being distinguishable with no direct observation of the process. Here, we compare the 2022 Hunga submarine deposits with prior exemplar deposit studies that include density currents generated from the ocean-entry of pyroclastic density currents, mass-wasting events, and submarine eruptions (Table 1; Figure 5).

Submersion of terrestrial-initiated pyroclastic density currents (PDCs) at the shoreline is a common feature at near-shore volcanoes (e.g., following strombolian eruptive activity at Stromboli 2019-2022[67–69] and eruption column collapses at La Soufrière[70]). Subsequent transformation of PDCs into underwater volcaniclastic density current is recorded in numerous submarine deposits (e.g., 1883 Krakatau eruption[71,72]) and has been observed directly (e.g., 2003 dome collapses at the Soufriere Hills Volcano, Montserrat[10,73]). Deposits of water-entering PDCs are typically unidirectional when parent flows are triggered by smaller, localized, events including dome collapses[3] and crumbling lava flow fronts[74]. In contrast, column collapses (e.g., the 1883 Krakatau eruption) can trigger radial dispersions of water-entering PDCs and ensuing underwater currents deposits[72,75]. For eruptions smaller than Hunga, runouts associated with shoreline crossing PDCs are much shorter (Table 1). In larger eruptions (of similar VEI to Hunga) density currents have been inferred to travel equivalent (or greater) distances, albeit with primarily unidirectional runouts except in the case of relatively small ocean island volcanoes[52,76]. Near-shore deposition and only minor subaerial erosion associated with water-entering PDCs differs greatly from the proximal flank erosion at Hunga (Figure 1). Distally, the Hunga 2022 deposits record stacked fining-upward sequences distinct from the single-layered, typically normally-graded and massive deposits recording infrequent planar and ripple laminations observed in deposits sampled offshore Montserrat and Krakatau[10,72]. This differing deposit structure is likely due to the contrast between continuous, pulsed, and multidirectional flows sustained by high eruption rates from a shallow vent (e.g. Hunga), as opposed to a single large volume PDC (e.g. Montserrat).

Submarine volcanoes record a variety of eruption styles that trigger underwater-density currents[77]. Very-shallow (< ~100 m b.s.l.) Surtseyan eruptions, e.g., the 13 ka Black Point eruption[78] and 18.6 ka Pahvant Butte eruption[79], driven by explosive phreatomagmatic fragmentation generate density currents with multidirectional runout, creating deposits that

fine upwards, with planar and ripple laminations, comprising compositionally homogenous volcaniclastic material. However, they have far-shorter runouts (>14-25 km) likely resulting from the order of magnitude smaller eruptive volumes (e.g. 0.8 km^3) than the 2022 Hunga eruption. This highlights the importance of eruption volume and mass flux in controlling resultant density current runout.

Magmatic volatile-driven explosive shallow-submarine eruptions (<500-1000 m b.s.l.) can trigger density currents via combinations of submarine eruption-column collapse and fountaining[2,80]. The size and runout of currents triggered during relatively small eruptions (VEI 0-2; e.g. 2008 North-West Rota-1 eruption), are far smaller than those identified at Hunga[81,82]. No field studies document all diagnostic criteria for more voluminous eruptions at these depths. Proximal deep-sea coring and in-situ deposits often record massive (or diffusively bedded) pumice-rich deposits[9], may display upward-coarsening[83], and, in some cases, evidence welding indicative of heat-retention and gas-supported flow[84]. Challenges in sampling close (<20 km from the volcano) to Hunga Volcano prevents direct comparison, but we see no evidence of high-temperature emplacement. Furthermore, whilst eruptions of similar or greater magnitude to Hunga 2022 can generate multidirectional currents with similar runouts to Hunga, they record no distal preservation of pulsed flows in the form of stacked fining upward sequences[9].

At deep-submarine volcanoes (> 500-1000 m b.s.l), suppression of explosive magmatic fragmentation due to water-pressure largely limits density current formation to triggering by mass-wasting. However, at these depth fuel-coolant interactions can trigger explosive eruptions such as the 2012 Havre eruption, where the lateral dispersion of dilute volcaniclastic density currents from a submarine eruption column emplaced multidirectional, thin (2-14 cm), and massive volcaniclastic deposits of lithic-poor cohesive ash[85–87]. However, the short runout (>5.5 km), and absence of both fining-upward sequences and erosional bedforms distinguish these deposits from those observed at Hunga.

Mass-wasting events at marine volcanoes can produce underwater volcaniclastic density currents, even during quiescent periods absent of eruptive activity[88]. Small mass-wasting events, associated with localised dome and caldera-wall collapses, frequently occur at submarine volcanoes including Havre[85,86], Kick em' Jenny[4,89], and Monowai[90]. Larger mass-wasting events relate to volcanic flank collapses such as the 150 ka Icod landslide, Tenerife[91–96], and the 1888 Ritter Island lateral collapse, Papua New Guinea[97]. Despite variation in mobilized volume (<0.1-320 km^3) and runout (5.5-1300 km), mass-wasting deposits are consistently unidirectional and share congruent morphologies: proximal fault

scar, proximal large blocks (decimetre-to-kilometre diameters), and a medial-to-distal elongate and spreading lobate morphology, unrecognised in the Hunga bathymetry. Medially-to-distally, mass-wasting deposits are characterized by debrites[86,97], unrecorded in any Hunga sediment cores. The distal limits of mass-wasting deposits commonly record volcaniclastic turbidites e.g., Ritter Island[97] and Icod landslide[51]. Distal turbidites of the Icod landslide feature stacked fining upward sequences like those observed in the Hunga 2022 deposits; however, these sequences are attributed to successive phases of flank collapse, which can be distinguished via unidirectional runout and heterogenous lithology. Mass-wasting deposits, specifically those associated with secondary remobilization of previously erupted volcaniclastics, record heterogenous volcanic compositions and high proportions of lithic and biogenic components – in contrast to the dominant homogenous juvenile composition and minimal lithic and biogenic proportions of the Hunga deposits.

Despite some shared characteristics, we conclude that deposits of density currents triggered by the vertical entry of volcaniclastic material, via column collapse and fountaining, can be reliably distinguished from other initiation mechanisms. This is on the basis of their multidirectional dispersion, extensive runout, repeated stacks of fining-upward sequences following unsteady, pulsed delivery of material, and the particularly erosive scours and bedforms that sculpt the flanks of the volcano (trains of crescentic scours and incised channels).

Comment 04: Even important previously published observations from the same eruption at the same volcano, are also not incorporated such as the recent Barone et al., 2023 GRL paper showing that Fall deposits are also an important part of this overall eruption story.

Response 04: Thank you for this comment. We have modified and extended the section "*The 2022 eruption of Hunga volcano*" to incorporate additional information and observations of the eruption: (1) more detailed description of the volcanic edifice and associated caldera depths, (2) previous volcanic activity, and (3) eruption triggers and processes.

- **Lines: 56-65:** *Hunga Volcano is a partially-submerged shallow-submarine caldera on the Tofua Arc (Figure 1)[16]. The almost entirely submerged volcanic edifice rises from water depths of 1800 m, and prior to the 2022 eruption, recorded a ~4 km wide caldera with crater floor depths < 200 m below sea level and the rim islands Hunga Tonga and Hunga Ha'apai peaking at 114 m above sea level[16]. Post-caldera collapse ~900 years ago volcanism at Hunga had comprised infrequent low-intensity Surtseyan and*

phreatomagmatic activity[17,18]. On January 15th, 2022, following three weeks of minor Surtseyan volcanic eruptions, activity at Hunga Volcano rapidly escalated into the most explosive volcanic eruption of the 21st century[19,20]. Explosive activity was likely triggered by seawater ingress into the magmatic system and the intrusion of a more primitive magma[21]. Erupted glass H_2O concentrations are consistent with magmatic fragmentation taking places at a subsurface depth of 400-1000 m[22]

Additional references are added to further illustrate that the eruption plume was water-vapour rich, such as Baron et al., (2023), which supports the work done by Wu et al., (2025) and Mastin et al., (2024):

- **Lines 67-71:** *The eruptive phase lasted around 11 hours[25] – with several large explosions between 04:15-04:25 UTC (all times are UTC)[26], triggering tsunamis[27] and atmospheric shockwaves that circumnavigated the globe several times[28]. Between 04:15-04:40 increasing mass eruption rate (MER) caused rapid growth of the water-dominated column[22,29,30] to ~58 km[31].*

We have also referenced Kelly et al., (2024) to further highlight that most of the erupted material was directly supplied into the water surrounding Hunga volcano based on minimal airfall deposits, supporting our hypothesised methods of density current formation.

- **Lines 178-180**: *Despite the evacuation of ~6.5 km^3 of magma (Dense Rock Equivalent, DRE) during the eruption, the 58 km high eruption column was tephra poor (<0.1-0.2 km^3 of erupted volume)[22,29,30]. Instead, the bulk of erupted material entered the ocean[22,24,54].*

Comment 05: I hesitate to mention it, but the current lax approach to placing these results within the broader literature seems designed to overemphasize the supposed uniqueness of the insights presented, a framing that does not come across as particularly scholarly, or collegial. The literature review is especially limited. It draws on a small set of terrestrial or littoral analogues and “old timey” literature and yet omits a substantial body of work from other “explosive” (a dI use that term broadly) submarine volcanoes.

Response 05 Thank you for this comment. This was not the intention.

Comment 06 Volcanological terminology: This is used in a way that feels overly broad and imprecise. For example, the term explosive encompasses a wide range of fragmentation and

dispersal conditions, from conduit and vent processes to column dynamics and surface interactions. Greater precision in defining and applying such terms would help clarify the processes the authors intend to emphasize and better link them to relevant mechanisms. Given how few papers there are on the topic, the authors could define and apply volcanological terms more precisely, especially those relating to fragmentation and dispersal processes. The term “explosive”, for instance, is applied in a generic way without clarifying which aspects of fragmentation and dispersal processes are relevant in this case and different or like other notable cases. Plus, the loose application of terrestrial analogue terms to underwater phenomena involving volcanic product dispersal (both lavas and clastics) tends to under-emphasize the importance of key differences between the easier to observe terrestrial settings, and more difficult but volumetrically important submarine cases. Processes that arise from conduit and vent dynamics and eruption column dynamics as they operate under air, water, or ice, all lead to tephra and tephra deposits, each of which have different implications for interpreting the deposits, are essentially left assumed here. It has even recently been shown that deep submarine volcanism leading to substantial clastic aprons at nearby West Mata volcano result from implosive, rather than explosive volcanism (Murch et al.2022 and references therein). This is not to say that some or even most phenomena in the 2022 HTTH eruption were not explosive but instead used to point out that the term “explosive” is too vague when being used to presume that all tephra was produced in explosive fashion, and too terrestrial centric to be used in the generic way that is being done here. One can also make the case that by forcing subaqueous primary and secondary volcaniclastic sequence description into the terrestrial analogue naming convention the manuscript is losing detail about differences. One should be able to compare and contrast to illuminate the range of operating processes. Of course, wider recognition of literature would help a lot here too.

Response 06: Many thanks for this comment, we agree that terminology is important and that it’s usage can be challenging. We clarify the proposed explosive processes that triggered the 2022 Hunga eruption and reference them within the manuscript:

- **Lines 63-65:** *Explosive activity was likely triggered by seawater ingress into the magmatic system and the intrusion of a more primitive magma*[21]. *Erupted glass H_2O concentrations are consistent with magmatic fragmentation taking places at a subsurface depth of 400-1000 m*[22].

We have reworded the manuscript throughout to almost entirely remove all usages of the phrase “*explosive eruption*” and instead refer to the process that is occurring. For example:

- **Lines 215-219:** *Multidirectional flow of underwater volcaniclastic density currents is a recorded feature of column collapse at other shallow-submarine volcanoes e.g.,*

Santorini[57]. Additionally, terrestrial eruptions of equivalent magnitude to the Hunga 2022 eruption e.g., 1991 eruption of Mount Pinatubo (3.7-5.4 km^3 of erupted magma) record column-collapse triggered pyroclastic density currents dispersing radially away from the volcanic edifice[58].

- **Lines 257-259:** *Submersion of terrestrial-initiated pyroclastic density currents (PDCs) at the shoreline is a common feature at near-shore volcanoes (e.g., following strombolian eruptive activity at Stromboli 2019-2022[67–69] and eruption column collapses at La Soufrière[70]).*

- **Lines 259-262:** *Subsequent transformation of PDCs into underwater volcaniclastic density current is recorded in numerous submarine deposits (e.g., 1883 Krakatau eruption[71,72]) and has been observed directly (e.g., 2003 dome collapses at the Soufriere Hills Volcano, Montserrat[10,73]).*

- **Lines 264-265***: In contrast, column collapses (e.g., the 1883 Krakatau eruption) can trigger radial dispersions of water-entering PDCs and ensuing underwater currents, akin to the Hunga 2022 eruption deposits[72,75].*

- **Lines 277-281:** *Submarine volcanoes record a variety of eruption styles that trigger underwater-density currents[77]. Very-shallow (< ~100 m b.s.l.) Surtseyan eruptions, e.g., the 13 ka Black Point eruption[78] and 18.6 ka Pahvant Butte eruption[79], driven by explosive phreatomagmatic fragmentation generate density currents with multidirectional runout, creating deposits that fine upwards, with planar and ripple laminations, comprising compositionally homogenous volcaniclastic material.*

- **Lines 286-287:** *Magmatic volatile-driven explosive shallow-submarine eruptions (<500-1000 m b.s.l.) can trigger density currents via combinations of submarine eruption-column collapse and fountaining[2,80]*

- **Lines 298-303**: *At deep-submarine volcanoes (> 500-1000 m b.s.l), suppression of explosive magmatic fragmentation due to water-pressure largely limits density current formation to triggering by mass-wasting. However, at these depth fuel-coolant interactions can trigger explosive eruptions such as the 2012 Havre eruption, where the lateral dispersion of dilute volcaniclastic density currents from a submarine eruption column*

emplaced multidirectional, thin (2-14 cm), and massive volcaniclastic deposits of lithic-poor cohesive ash[86-87].

We have outlined what is means by the term "*underwater volcaniclastic density current*" and included the reference to the paper that first described these:

- **Lines 34-42:** T*he formation of these currents is attributed to: (1) volcanic eruptions where water mixes with pyroclasts erupted from submarine vents[9], (2) terrestrially initiated pyroclastic flows that reach the shoreline[10], or (3) mobilization of volcaniclastic material during mass-wasting events that may not be related to an eruptive episode (e.g., volcanic flank collapse, oversteepening of the coastal shelf, edifice collapse etc)[11]. Due to the myriad mechanisms of their formation the subsequent density currents record a broad spectrum of structures, behaviours, and dynamics[2,12]. Here, the term 'underwater volcaniclastic density current' is employed to cover the full-range of density currents produced in volcanic settings, from gas-rich and high-temperature pyroclastic density currents, and water-supported and cool volcaniclastic turbidity currents, to debris flows.*

We have provided definitions of turbidity currents and high-density turbidity currents:

- **Lines 118-129:** *The combination of normal grading, sharp base, poor-to-moderate sorting, planar and ripple laminations, and volcaniclastic material characterising Unit 1 are diagnostic of emplacement by an underwater volcaniclastic sediment density current; more specifically a volcaniclastic turbidity current[36]. These are sediment gravity currents in which sediment is held in suspension by fluid turbulence, driven to lateral flow by their excess density relative to the surrounding ambient water[37]. The bipartite structure of Unit 1 indicates the parental turbidity current was strongly density stratified with a near-bed high-concentration coarse-grained flow regime dominated by tractional flow and bedload transport (Unit 1a), and a dilute upper layer transporting fine material recorded in the beige planar-laminated muds (Unit 1b)[38–41]. The depositional architecture of Unit 1 is typical of that formed by a high-density turbidity current where turbulent and tractional-bed reworking processes are dampened but not inhibited within a dense sediment-concentrated near bed layer (Unit 1a) and there is a distinct transition into an overlying turbulent dilute flow (Unit 1b), which distinguishes these currents from debris flows[36].*

We have added Figure 5, which illustrates, through four schematics, the different processes at marine volcanic settings that may trigger underwater volcaniclastic density currents. These schematics are provided to facilitate clearer comprehension for readers and reduce ambiguity in our usage of terminology and processes.

As discussed above, we have also greatly expanded the literature comparisons included.

Comment 07 Summary: Given these points, the current framing raises concerns about fit for this publication venue. A more accurate title like "An Example of Submarine Pyroclastic Density Currents: Observations and Inferences from the HTHH 2022 Eruption" would better reflect the work's current scope, though it and also highlight its relatively limited appeal. To strengthen the manuscript for a wide audience venue such as this, I suggest:

- Broadening literature engagement, particularly with submarine volcanic deposits from both similar and contrasting settings.
- Incorporating relevant prior observations from the same eruption
- Define key volcanological terms with more precision.
- Draw out the importance of this study for seabed science more generally With a clearer framing, richer literature engagement, greater terminological precision, and a non-specialist angle to the important consequences and implications of these observations, this manuscript could better meet the expectations for publication in this journal.

Response 07: Many thanks for this summary. As outlined in our responses above we have:

(1) Broadened the literature included
(2) Included discussion of previous eruptions
(3) Defined our terms more clearly or removed them where they were too vague
(4) Reframed the discussion and changed the title.
(5) Additional figure illustrating the complex processes we discuss

We hope this satisfies the reviewers concerns and we welcome any further discussion on any of these points.

Reviewer #2

Comment 1: I have finally got the chance to go through the paper by Nash et al. on submarine geological record of Hunga volcano, which erupted heavily in 2022, and have found it very interesting. I have enjoyed reading it, as the paper is well structured and innovative, and timely too.

Response 1: Thank you for your review and valuable comments. In the accompanying updated manuscript, we have considered your concerns and suggested revisions and have made appropriate changes to better clarify and focus our research aims, findings, and conclusions. We have expanded on the bathymetric acquisition to make sure the data presented can be adequately assessed.

Comment 2: My only major concern is about some absence in recalling the methodology in the main body of the text. I say this because looking at the main parts of the paper it just seems a field work paper, which is OK, but I would highlight more the bathymetric survey also in the main body too; the methods adopted for this work are essential to get to the results, etc...

Response 2: Thank you for this comment. We agree that the bathymetric surveys play an essential role in identifying the impact of the eruption on the seafloor, characterising the underwater volcaniclastic density current deposits, and differentiating different current initiation mechanisms. We would argue that we recall and reference the results of the bathymetric surveys numerous times throughout the manuscript both in the main text and through reference to Figure 1 to support our interpretations:

Bathymetry is recalled when interpreting the structure of the 2022 eruption deposit:

- **Lines 150-154**: *Absence of a black sand-silt base in SW-111 might be due to waning and deposition of the coarsest basal sediment in flow and/or topographic influence. Between*

Hunga Volcano and the location of SW-111 are a series of high elevation ridges which may have partially impeded the current(s) resulting in segregation of a dilute upper layer that surmounted the ridge without the basal dense component[8] (Figure 1).

Bathymetry is recalled when illustrating the runout, distribution, and geomorphology of 2022 eruption deposit:

- **Lines 77-82**: Repeat pre- and post-eruption bathymetric surveys and deposits sampled from sediment cores collected two months post-eruption reveal that volcaniclastic sediments were dispersed in multiple directions, radiating from Hunga Volcano for over 100 km (Figure 1, Supplementary Figure 1 and Supplementary Table 1). These currents cut deep channels and scours on the upper flanks of the volcano, and deposited < 40 m thick lobate deposits at an abrupt slope break 4-5 km from the rim[13] (Figure 1).

- **Lines 208-211**: *Thick (<40 m) depositional lobes emplaced at the first major slope break away from the volcanic edifice of Hunga (Figure 1), and coarse/competent deposits that could not be cored close to (<20 km) Hunga[14], may record similar sudden emplacement of the coarsest/densest material from the initial currents.*

- **Lines 219-222**: *Pre- and post-eruption bathymetry show that the volcaniclastic material around Hunga Volcano was topographically channelled down the volcanic flanks at multiple locations radially around the edifice, steered along pre-existing channels or eroding new ones[13,15]*

We recall the bathymetry when comparing the deposits of density currents emplaced by different mechanism:

- **Lines 172-177**: *Far-reaching volcaniclastic turbidity currents (> 100 km) have been attributed to volcanic flank collapse[51] and water-entering pyroclastic density currents[52]. Yet, there is no sedimentological or bathymetric evidence for either of these processes occurring during the 2022 Hunga eruption. Instead, we observe: (1) caldera collapse[24,53,15]; 2)) erosive bedforms on the upper volcanic flanks; (3) large-scale deposition (<40 m) following the first slope break; (4) multidirectional deposition of centimetre-to-decimetre-thick volcaniclastic turbidites >111 km from Hunga volcano.*

- **Lines 269-270**: Near-shore deposition and only minor subaerial erosion associated with water-entering PDCs differs greatly from the proximal flank erosion at Hunga (Figure 1).

- **Table 1 –** geomorphology of the Hunga eruption deposits is constrained from the bathymetry

---

Comment 3: In terms of interpretation, I agree that longer flow runouts may depend on eruptive mechanisms, which is something enounced clearly (and from the field deposits) in https://onlinelibrary.wiley.com/doi/epdf/10.1111/ter.12561 , which will strengthen interpretation.

Response 3: Thank you for this comment. This is an interesting paper, that does broadly support our discussion on volcanic processes directly impacting flow runout and distribution. As such, we have extended our discussion on flow runout and added this reference:

- **Lines 235-237:** *Comparable deposit geometries and long-runouts emplaced by terrestrially-sourced pyroclastic density currents triggered by sustained column-collapses or vertical jet dynamics support this interpretation[66].*

Reviewer #3

Comment 01: This paper is fluently written and well structured, and it addresses a topic of actual and wide interest in the volcanological community. It adds to the growing body of research publications on the large Hunga 2022 eruption the investigation of sediment cores collected around and away from the Hunga caldera. The focus is on the investigation of four cores representing a medial to distal radial profile to the SW through the syn-eruptive volcaniclastic turbidite facies.

Response 01: Thank you for your review and valuable comments. In the accompanying updated manuscript, we have considered your concerns and suggested revisions and have made appropriate changes to better clarify and focus our research aims, findings, and conclusions. In response to your review we have changed the title, modified our unit classifications and added clarifications to our core descriptions and figures which we hope clarify most of the points raised.

Comment 02: I suggest including Hunga 2022 in the title because the strong point of this paper is what it can contribute to the understanding of that eruption. This is not a review of seafloor deposits of submarine eruptions in general, as the title suggests. The last paragraph aims a bit in that direction, but I will comment on that further below.

Response 02: Thank you for raising an excellent point. We agree that the title does not wholly-represent the results or discussions presented in the manuscript. To address this, we have significantly revised the title from "*The Seafloor record of submarine explosive eruptions"* to "*Voluminous, far-reaching, and multidirectional volcaniclastic density current deposits are key records of explosive marine eruptions*" We address your comments regarding contents and direction of the last two paragraphs below. See Response 12, and Reviewer 1 responses 02-03.

Comment 03: The interpretation that the turbidity current is fed by jetted and collapsed material from the eruption column is, of course, an important result that will also be valuable for future work elsewhere.

Response 03: Thank you for this comment.

Comment 04: In the description and discussion, I find it less helpful that the entire deposit is called unit 1, unit 2 being the pre-eruption sediment. Earlier work of Seabrook et al. (2023), partly involving the same authors, has already characterized unit 2 as pelagic clay sediment underlain by older volcaniclastic sediment; this could briefly be mentioned here. I then suggest dividing unit 1 into three subunits, say 1A, 1B, 1C, with appropriate labels in Figs. 1-3, to then interpret these three parts as done in the text. 1A would then become the deposit of the high-concentration flow regime, 1B the low concentration flow regime, and 1C the final fallout over some months.

Response 04: Thank you for this useful observation. We agree that separating the 2022 eruption deposit into separate subunits aids visualization of the eruption deposits and the specific density current dynamics/processes responsible for the deposit structure. To address this comment, we separate the eruption deposits into three units,1a, 1b, and 1c which represent the high-concentration near bed layer, the dilute upper portion of the flow, and the fallout, respectively. These three units are described and interpreted in the following sections:

- **Lines 86-98:** *Sediment cores sample two distinct depositional units. The uppermost unit (unit 1) is interpreted as the 2022 eruption deposits lying unconformably above pre-eruption sediments (unit 2; Figure 1 and Figure 2; supplementary information). Unit 1, divided into three subunits 1a, 1b, and 1c, is characterized by stacked fining-upward sequences of volcaniclastic sediments with a sharp basal contact. At the base of Unit 1is a centimetre-to-decimetre thick layer of black, poorly-to-moderately sorted, sand-silt sized volcaniclastics (Unit 1a). Unit 1a overall displays normal grading, but with some abrupt internal grainsize changes, planar laminations, climbing ripples, and disturbed laminations. Directly above Unit 1a, Unit 1b locally features black sand-silt layers (one to six) of varying thickness (centimetre-to-millimetres) separated by layers of beige silt-clay sized material. The black sand-silt layers are texturally and visually similar to the basal black sand of Unit 1a. Different layers are distinguished by distinct colour (Figure 2) and grainsize changes (Figure 3). Beige silt-clay layers are characterized by overall upward-fining, poor-sorting, and millimetre-scale planar laminations. Unit 1 is topped by Unit 1c, a thin, centimetre (< 4 cm), layer of structureless clay.*

- **Lines 118-135**: *The combination of normal grading, sharp base, poor-to-moderate sorting, planar and ripple laminations, and volcaniclastic material characterising Unit 1 are diagnostic of emplacement by an underwater volcaniclastic sediment density current; more specifically a volcaniclastic turbidity current[36]. These are sediment gravity currents in which sediment is held in suspension by fluid turbulence, driven to lateral flow by their excess density relative to the surrounding ambient water[37]. The bipartite structure of Unit 1*

indicates the parental turbidity current was strongly density stratified with a near-bed high-concentration coarse-grained flow regime dominated by tractional flow and bedload transport (Unit 1a), and a dilute upper layer transporting fine material recorded in the beige planar-laminated muds (Unit 1b)[38–41]. The depositional architecture of Unit 1 is typical of that formed by a high-density turbidity current where turbulent and tractional-bed reworking processes are dampened but not inhibited within a dense sediment-concentrated near bed layer (Unit 1a) and there is a distinct transition into an overlying turbulent dilute flow (Unit 1b), which distinguishes these currents from debris flows[36]. Both unit 1a and 1b contain disturbed planar laminations (Figure 1 and Supplementary Figure 1). The cause of this disturbance is unknown, but can result from syn-depositional shear, rapid sediment loading, fluid escape, or deformation during coring[42–44].Continued settling of suspended sediments was documented by video and water-column sampling three months after the eruption[14,15]. The uppermost layer of thin clay (<4 cm) relates to this settling material sourced either from ash-fallout or material lofted by density currents or some combination of both (Unit 1c)[45].

- **Lines 156-163**: *Repeated coarse black sand-silt intervals within Unit 1b consistently thin and fine up-core and along the flow pathways away from Hunga Volcano. Medially (<80 km) contacts between black sand-silt and beige silt-clay layers are sharp and visibly well defined, recording clear grainsize and colour changes, before becoming progressively diffuse with distance from Hunga Volcano (>80 km). These coarser layers reveal repeated reinvigoration of high-concentration near-bed fluxes (Unit 1a) that could be produced by a pulsed turbidity current with temporally-unsteady fluxes, a series of distinct and individually triggered turbidity currents, reflection of flows off surrounding seafloor relief, and/or different travel paths of synchronously-triggered flows[49,50].*

These three subunits are identified in the following figures:

Figure 1 – Sedimentary logs have been edited to illustrate the locations of subunits 1a-c up core and between the cores of the proximal-to-distal SW transect.

Figure 2 – XRF plots have been edited to illustrate the locations of subunits 1a-c up-core. The green background has been replaced with three distinct shades of pink.

Supplementary Figure 1 - Green background identifying the 2022 eruption deposits replaced with three separate colours identifying the eruption deposit subunits 1a – dark pink, 1b -pink, 1c – light pink

---

Supplementary Figure 5 - Green background identifying the 2022 eruption deposits replaced with three separate colours identifying the eruption deposit subunits 1a – dark pink, 1b -pink, 1c – light pink

---

Comment 05: For example, Seabrook et al. identify the uppermost 10 cm of core SW-80 as the late ash fall, similar to the interpretation of push cores in Chaknova et al. (2025; also involving some of the present authors). This is not recognizable in the present presentation where it is difficult to identify in the figures the boundaries mentioned in the text. If this is done because the authors interpret the top part (of core 80 and others) differently, then this must be discussed.

Response 05: Thank you for this comment. We agree that the location and thickness of the ash-fall subunit within the 2022 eruption deposits was ambiguous in the current figures and text. To address this:

- The ash-fall unit has been re-named as unit 1c in the main manuscript text
- The maximum thickness of unit 1c has been included in the main manuscript text
    - **Lines 133-135:** *The uppermost layer of thin clay (<4 cm) relates to this settling material sourced either from ash-fallout or material lofted by density currents or some combination of both (Unit 1c)[45].*
- Location of unit 1c has been illustrated on the sedimentary logs in Figure 1 and Supplementary Figure 1, and the XRF plots in Figure 2 and Supplementary Figure 5.

Comment 06: The use of "high-concentration" should perhaps be discussed a bit more. While it the textural and structural changes upward clearly reflect a decrease in the current's sediment concentration, the concentration near base was not so high that formation of tractional bed-load processes was suppressed

Response 06: Thank you for pointing this out. We agree that the usage of "*high-concentration*" without providing/referencing a definition does introduce ambiguity regarding the flow-structure and behaviour being inferred. To address this issue, we specifically identify the turbidity currents as '*high-density turbidity currents*' using the deposit and flow descriptions outlined in Talling et al., (2012; https://onlinelibrary.wiley.com/doi/full/10.1111/j.1365-3091.2012.01353.x); Turbidity currents dampen but do no inhibit entirely tractional-bed load processes, especially within a high-concentration, denser, near-bed traction carpet.

- **Lines 118 – 129:** *The combination of normal grading, sharp base, poor-to-moderate sorting, planar and ripple laminations, and volcaniclastic material characterising Unit 1 are diagnostic of emplacement by an underwater volcaniclastic sediment density current; more specifically a volcaniclastic turbidity current[36]. These are sediment gravity currents in which sediment is held in suspension by fluid turbulence, driven to lateral flow by their excess density relative to the surrounding ambient water[37]. The bipartite structure of Unit 1 indicates the parental turbidity current was strongly density stratified with a near-bed high-concentration coarse-grained flow regime dominated by tractional flow and bedload transport (Unit 1a), and a dilute upper layer transporting fine material recorded in the beige planar-laminated muds (Unit 1b)[38–41]. The depositional architecture of Unit 1 is typical of that formed by a high-density turbidity current where turbulent and tractional-bed reworking processes are dampened but not inhibited within a dense sediment-concentrated near bed layer (Unit 1a) and there is a distinct transition into an overlying turbulent dilute flow (Unit 1b), which distinguishes these currents from debris flows[36].*

Comment 07: When discussing a possibly pulsed turbidity current more specific reference to the repetitions particularly in core 80 and 100 should be made. For example, the graphic interpretation of core 80 in Fig. 1 suggests 6 pulses but the upward grain size decrease in Fig. 3 appears to be fairly steady.

Response 07: Thank you for this comment. We agree that the grainsize changes in the more distal cores are not ash sharp as the more proximal cores. Whilst the grainsize changes in SW-80 are not as sharp as SW-64 there are noticeable increases and decreases near the base of the deposit that align with black sand layers in the sediment core, before transitioning into a steady decrease in grainsize up-core. At 80 km from source, the overall grainsize of a waning density current in which most of the material was deposited within 8 km of Hunga volcano will be relatively fine and so the grainsize changes will be less drastic.

We do directly reference the number of pulses in the sediment cores:

- **Lines 92-95:** *Directly above Unit 1a, Unit 1b locally features black sand-silt layers (one to six) of varying thickness (centimetre-to-millimetres) separated by layers of beige silt-clay sized material. The black sand-silt layers are texturally and visually similar to the basal black sand of Unit 1a.*

- **Lines 238-244**: *Seismic signals generated during the 2022 Hunga eruption[24] coupled with plume growth models[19,30] indicate maximum MER between ~04:15-05:10, before declining*

from 05:30 onwards. The majority of the 6.5 km^3 of pyroclastic material was deposited onto the seafloor during the 50-minute window of maximum MER, explaining the lack of fine-grained mud capping between black sands. Episodic and waning eruption intensity created variable but overall decreasing delivery of pyroclastic material with time, likely explaining the up-core fining and black sand-silt intervals in Unit 1, with at least six pulses of pyroclastic material delivery recorded (e.g., core SW-80).

Comment 08: What is the origin of the convolute bedding in core 100? Can this be enhanced by topography?

Response 08: Thank you for this comment, having re-evaluated the deposits we have decided that convoluted bedding was the incorrect terminology/descriptor for this sedimentary structure. Instead, this structure has been re-labelled in the *Figure 1 Legend* as disturbed planar bedding – a more appropriate descriptive term; planar laminations are ubiquitous across the 2022 eruption deposits and relate to the deposition from a waning and turbulent density current. The exact cause of the planar lamination disturbance is unknown but may be a result of syn-depositional shear, rapid sediment loading, fluid escape or deformation during coring. We have included reference to disturbed planar lamination in the main manuscript text:

- **Lines 91-92:** *Unit 1a overall displays normal grading, but with some abrupt internal grainsize changes, planar laminations, climbing ripples, and disturbed laminations.*

- **Lines 130-132:** *Both unit 1a and 1b contain disturbed planar laminations (Figure 1 and Supplementary Figure 1). The cause of this disturbance is unknown, but can result from syn-depositional shear, rapid sediment loading, fluid escape, or deformation during coring[42–44].*

Comment 09: The “waning flow” interpretation should make use of the ripple images in suppl. Fig. 1 (otherwise, what are they good for??)

Response 09: Thank you for this suggestion. We agree, these ripple images are an excellent visual indicator of deposition from a waning flow. We address this, but making specific reference to the ripples and Supplementary Figure 1 in the text:

- **Lines 140-142:** *Moving away from the volcanic edifice deposits surface ripples decrease in size before disappearing in the most distal locations (Supplementary Figure 2).*

Comment 10: To be honest, I do not like the section lines 195-240 very much. The first paragraph up to line 204 summarizes the results for this Hunga case. This would be a good place to deepen previous discussion, for example by addressing the question of the different influences on the fining upward and with distance such as progressive loss of coarser particles from the flow, waning eruption, or the distally fining fallout from the plume (Fig. 4).

Response 10: Thank you for this comment. We believe that we do address the topic of different influences on the up-deposit and distal fining of the 2022 submarine eruption density current deposits in the existing manuscript. We illustrate that the structure of the turbidity current, specifically a high-density turbidity current, results in a vertically density and grainsize stratified deposit:

- **Lines 118-129:** *The combination of normal grading, sharp base, poor-to-moderate sorting, planar and ripple laminations, and volcaniclastic material characterising Unit 1 are diagnostic of emplacement by an underwater volcaniclastic sediment density current; more specifically a volcaniclastic turbidity current[36]. These are sediment gravity currents in which sediment is held in suspension by fluid turbulence, driven to lateral flow by their excess density relative to the surrounding ambient water[37]. The bipartite structure of Unit 1 indicates the parental turbidity current was strongly density stratified with a near-bed high-concentration coarse-grained flow regime dominated by tractional flow and bedload transport (Unit 1a), and a dilute upper layer transporting fine material recorded in the beige planar-laminated muds (Unit 1b)[38–41]. The depositional architecture of Unit 1 is typical of that formed by a high-density turbidity current where turbulent and tractional-bed reworking processes are dampened but not inhibited within a dense sediment-concentrated near bed layer (Unit 1a) and there is a distinct transition into an overlying turbulent dilute flow (Unit 1b), which distinguishes these currents from debris flows[36].*

We further go on to illustrate that the uppermost section of the eruption deposit is comprised of ash-fallout.

- **Lines 133-135**: *Continued settling of suspended sediments was documented by video and water-column sampling three months after the eruption[14,15]. The uppermost layer of thin clay (<4 cm) relates to this settling material sourced either from ash-fallout or material lofted by density currents or some combination of both (Unit 1c)[45].*

Distinguishing the source of the fallout remains challenging. Fallout from the subaerial plume likely took over a day to reach the seafloor (Chaknova et al., 2025). Fine material lofted into suspension by underwater density currents can remain in the water column for days to months. Ash from the 2022 eruption was still present suspended in the water-column months after the eruption (Seabrook et al., 2023). In both cases this material would be restricted to the top of the eruption deposit considering the density currents were triggered in a period of < 90 minutes which is consistent with the thin (<4.5 cm) structureless clay topping the sediment cores.

We then demonstrate that the distal fining seen in the eruption deposits, in combination with structural and architectural changes, is a characteristic of waning competency in an underwater density current:

- **Lines 137-144:** *Units 1a and 1b record sedimentary evidence for deposition from a sediment density current maintaining a high-concentration near-bed layer, with progressive current waning towards the SW (Figure 2 and3). Black sand-silt intervals thin and fine distally into centimetre-thick silt-mud layers becoming absent in the most distal core (SW-111). Moving away from the volcanic edifice deposits surface ripples decrease in size before disappearing in the most distal locations (Supplementary Figure 2). Unit 1a black-sand intervals record a transition from planar to ripple laminations distally and up-core, evidencing contemporaneous bedload transport and high suspended load fallout, diagnostic of turbidity current waning[36].*

When discussing the stacked fining upward sequences, we suggest that both the thinning and fining of the black sand layers (representing "*repeated reinvigoration of high-concentration near-bed fluxes";* **Line 160**) and the loss of well-defined black sand layers is representative of *"Episodic and waning eruption intensity"* (**Lines 241-242**). This is directly referenced in the manuscript:

- **Lines 241-244**: *Episodic and waning eruption intensity created variable but overall decreasing delivery of pyroclastic material with time, likely explaining the up-core fining and black sand-silt intervals in Unit 1, with at least six pulses of pyroclastic material delivery recorded (e.g., core SW-80).*

Ultimately, the up-core and distal waning of the 2022 eruption deposit is a combination of density current structure, density current evolution, eruption intensity, and ash-fallout from both plume and lofted material.

We feel that a discussion focused entirely on the specific behaviours and evolution of the density currents triggered during the 2022 eruption, without comparisons to other density currents and density current forming mechanisms, limits the usefulness of our findings.

Comment 11: Or are the “pulses” seen in cores 80 and 100 (but also SSW-48, SW-33 in supplementary) related to unsteady current formation, effects of topography on current, or unsteady eruption? I think such kind of discussions could deepen the impact of the paper.

Response 11: Thank you for this comment. Existing core and geochemical datasets (ITRAX uXRF) make it difficult to definitively identifying the exact cause of the “*pulses*” or fining-upward sequences in the 2022 Hunga eruption deposits. Firstly, “*pulse*” correlation between individual cores is challenging, as no cores share the exact same number of “*pulses*.” Secondly, the broad semi-quantitative approach of ITRAX uXRF was unable to provide any compositional distinction between individual “*pulses*” (Figure 2 & Supplementary Figure 3), and emerging data from the land deposits suggests little geochemical variation during the eruption (Cronin; Pers. Comm.). Identifying processes such as flow reflection or the influence of topography would likely require more (oriented) sediment cores.

Existing core data (e.g., absence of bioturbation, no hemipelagite, no ripple reversal, and distal amalgamation of sands) and the small-time frame in which the density currents were formed (< 90 mins) suggests either a stack of distinctly-pulsed turbidity currents or a single quasi-steady pulsed current that amalgamated distally. This formation mechanism is supported by the seismic record of the eruption (Cronin et al., 2025). As such, we have extended our discussion to highlight this corroboration:

- **Lines 187-190:** *The semi-continuous generation of underwater volcaniclastic density currents, revealed by seismic energy records, supports our interpretation that the repeat fining upward architecture of the 2022 deposits is indicative of deposition from a series of quasi-steady pulsed currents.*

Furthermore, we also relate the “*pulses*” to changing eruption intensity:

- **Lines 238-244*:*** *Seismic signals generated during the 2022 Hunga eruption[24] coupled with plume growth models[19,30] indicate maximum MER between ~04:15-05:10, before declining from 05:30 onwards. The majority of the 6.5 km^3 of pyroclastic material was deposited onto the seafloor during the 50-minute window of maximum MER, explaining the lack of fine-grained mud capping between black sands. Episodic and waning eruption intensity created variable but overall decreasing delivery of pyroclastic material with time, likely explaining*

the up-core fining and black sand-silt intervals in Unit 1, with at least six pulses of pyroclastic material delivery recorded (e.g., core SW-80).

Comment 12: On the other hand, I find comparison with Montserrat not helpful. This is not a typical or representative case for pyroclastic flows entering the ocean globally, it is a small dome eruption 50 times smaller in transported mass than the Honga case. There are other cases of much more voluminous pyroclastic flows that entered the sea radially and produced approximately radial distribution of turbidity currents (e.g., around Gran Canaria). Comparison of Honga with these deposits would yield totally different results.. Similarly with the comparison with flank failures. Naturally these do not occur radially although their turbidity currents can spread sectorially. The spreading of primary volcaniclastic turbidity currents as at Honga depends on the morphology of the volcano and the surrounding seafloor and need not always be radial. So, the major difference to flank failure is the heterogeneity of the volcaniclastic particles, which is not a new recognition. Thus, my advice would be to skip these comparisons and rather focus on a more in-depth interpretation of the Hunga deposits.

Response 12: Thank you for this insightful and critical feedback. Please see my earlier responses to Reviewer 1's comments, specifically Responses 02, 03, 06, and 07, as they address the same issues you have raised.

To summarize:

- Significant reframing & extension of the discussion
- Increased the number of case-studies used in deposit comparison to 16 (previously three)
- Provided rationale for using terrestrial and not directly comparable analogues to interpret the 2022 Hunga eruption deposits
- Addition of Figure 5 to illustrate the different density current forming mechanisms at marine volcanic settings

---

Comment 13: Line 66: the main focus is on the four cores named but the other (more proximal) cores are shown with their data in the supplement. Although most of these do not penetrate the entire deposit, they still provide valuable information, e.g. on where the stratified coarse facies occur, where the upper fine facies are thin or thick, … so I don't quite understand why this information is not exploited in the text.

Response 13: Thank you for this comment. In this study, we chose to focus on the four cores comprising the southwest proximal-to-distal transect as they penetrate the entire thickness of the 2022 eruption deposits, providing the most complete stratigraphic record of the eruption, and enabling the most confident interpretation of density current behaviour and eruption chronology. We feel that the incomplete deposit record in the remaining cores limits our confidence in providing robust interpretations. We agree that many of these cores do record import structural information including fining with distance (e.g., SW-20), stacked fining upward sequences (e.g. NW-26) and repeated injections of coarser darker sand (SSW-48). However, these structures are consistent with those present in the SW transect cores and do not reveal additional information on density current behaviour. Whilst we do not utilise the remaining cores to make direct interpretations about density current structure and evolution, they do provide critical constraints on density current dispersion. As such we directly reference these other sediment cores earlier within the manuscript as Supplementary Figure 1:

- **Lines 77 - 80**: *Repeat pre- and post-eruption bathymetric surveys and deposits sampled from sediment cores collected two months post-eruption reveal that volcaniclastic sediments were dispersed in multiple directions, radiating from Hunga Volcano for over 100 km (Figure 1, Supplementary Figure 1 and Supplementary Table 1).*

However, we recognise the importance of these sediment cores in providing insights into density current behaviours in different directions to the SW transect. As such, they are the focus of ongoing research aimed at correlating individual subunits up-core and between cores using complex geochemical analysis and componentry.

Comment 14: Line 45: "shallow" should be quantified here, indicate the actual water depths/range.

Response 14: Thank you for highlighting this missing information. We agree, that by not including actual water-depth information our definition of shallow-submarine, partially submerged, or submarine regarding the eruption is ambiguous and undefined. As such, this information is now included in the edited section "*The 2022 eruption of Hunga Volcano.*" Additional information on the depth interval of magmatic fragmentation is also provided.

- **Lines 56-59:** *Hunga Volcano is a partially-submerged shallow-submarine caldera on the Tofua Arc (Figure 1)[16]. The almost entirely submerged volcanic edifice rises from water depths of 1800 m, and prior to the 2022 eruption, recorded a ~4 km wide caldera with*

crater floor depths < 200 m below sea level and the rim islands Hunga Tonga and Hunga Ha'apai peaking at 114 m above sea level[16].

- **Lines 64-65:** *Explosive activity was likely triggered by seawater ingress into the magmatic system and the intrusion of a more primitive magma*[21]*. Erupted glass H_2O concentrations are consistent with magmatic fragmentation taking places at a subsurface depth of 400-1000 m*[22].

Comment 15: By the way, in the supplement Fig. 5 the cores SE-45 and SW-33 have been mixed up with respect to Fig. 4.

Response 15: Thank you for noticing the mistake. This has been corrected and the cores correctly labelled.

Comment 16: Line 147: replace "material" by "magma" because I assume that DRE has been calculated as lithic-free as usual.

Response 16: Thank you, we have replaced "*Material"* with "*volcaniclastic material*"

Comment 17: Line 162: …. trigger pulses of density ….

Response 17: Thank you, the sentence has been corrected.

Comment 18: Lines 168 ff. would be one place where reference to the proximal core information from the supplement could be helpful in moving this section a bit closer to observation rather than speculation….

Response 18: Thank you for this comment. We agree, and to address this we add a reference to Supplementary Figure 1 (all core logs):

- **Lines 205-208:** *Cores > 20 km from Hunga record no sedimentological evidence of gas-supported flow or high-temperature (>650ºC) emplacement (e.g., welding); hence we infer at least 20 km from Hunga Volcano the currents were cool, water-supported, turbidity currents (Supplementary Figure 1).*

Comment 19: Line 182: … caused currents to ….

Response 19: Thank you, the sentence has been corrected

Comment 20: Line 190: delete "as"

Response 20: Thank you, the sentence has been corrected

Comment 21: Fig. 1: B,C: replace outlined font by solid font. I found the outlined font very hard to read
C: the bathymetric work and interpretation was originally done in Seabrook et al. (2023) and this should be cited here. In fact, the related elaborate method section (line 413 ff..) suggests that this is original work by the present authors which is apparently not the case. D: insert subunit labels as I suggest in the text… Report water depth below the core label at each profile (the same in suppl. Fig. 4)

Response 21: Thank you for these comments. We agree that the outlined font on Figure 1A-C is rather illegible. As such, it has been replaced by non-outlined text with a transparent background to aid eligibility. We have edited the Figure 1 caption to include reference to both Seabrook et al., 2023 and Clare & Yeo et al., 2023 regarding the bathymetry. On Figure 1D subunits 1a, 1b, and 1c have been identified on the sediment logs, and added to the figure

legend. Water-depth has been added below core label at each profile (also added to Figure 2, Supplementary Figure 1, and Supplementary Figure 5).

The methods section has been re-written and re-structured to make it clear that the seafloor elevation difference layer was previously created by the authors of Seabrook et al., 2023 and Clare & Yeo et al., 2023:

- **Lines 582-584***: Post-eruption acquisition of bathymetric data, seafloor imagery, and sediment cores was previously conducted during the TESMaP (Tonga Eruption Seabed Mapping Project) TAN2206 research cruise onboard the R/V Tangaroa to the site of the Hunga eruption in April to May 2022 [14].*

- **Lines 586 – 598:** *Multibeam bathymetric data detailing the post-eruption topography of Hunga volcano, and the surrounding seafloor (Figure 1) was collected using a Kongsberg Simrad EM302 multibeam echosounder. Thorough description of the multibeam surveys, data collection methods, and data processing workflows are detailed in Mackay (2022). Seabrook et al (2023) and Clare & Yeo et al (2023) illustrated seafloor change following the January 2022 eruption by comparing bathymetric data collected by TESMaP with a pre-eruption digital elevation map (DEM) comprised of bathymetric data acquired both remotely and on two separate research expeditions: (1) 2017 satellite-derived bathymetry[98], (2) the May 2016 FK160407 Expedition on the RV Falkor using a Kongsberd Simrad EM302[99], and (3) November 2015 small boat survey using a WASSP multibeam echosounder[16]. These pre- and post-eruption DEMs were imported into ArcPro GIS where they were differenced to create an elevation difference map of the seafloor around Hunga highlighting regions of elevation loss (erosion) and gain (deposition)[13,15]. A thorough description of the workflow process to produce the elevation difference map is documented in Clare and Yeo et al, (2022).*

Comment 22: In all profile figure replace Depth by “Depth below seafloor” or “Depth in core”, in figures 1-3 as well as in the supplement figure 4 and 5 (supply Fig. 5 has no vertical axis labels at all)

Response 22: Thank you for this comment. We have modified every figure (Figure 1, Figure 2, Supplementary Figure 1, and Supplementary Figure 5) where there is a “*Depth (cm)*” axis, and replaced the label with “*Depth below seafloor (cm)”*

Comment 23: Fig. 2: what is the advantage of showing IMG and BRIGHT core images? Why not show each core image at the exposure that shows best the features which should be seen???
(it is not a true colour presentation anyway…)

Response 23: Thank you for this suggestion. We agree that having both the unmodified image and brightened image is unnecessary. To address this, we have removed the unmodified image from Figure 2 and Supplementary Figure 5. As mentioned in your comment, we keep the brightened image as it best shows the structure and architecture of the material in the cores. We also have replaced the low resolution brightened and laminograph images with higher resolution versions.

Comment 24: Fig. 3: avoid outlined font but, most of all, correct the scale of 500 centimetre ;-)

Response 24: Thank you noticing this error. We have removed all the outlined fonts and corrected the scale bar to micrometres.

Comment 25: Supplementary: Check core 33 and 45 mixed up between Figs. 4 and 5

Response 25: Thank you for noticing this error. These have now been correctly labelled.

Comment 26: Fig. 3 caption has a reference 15 to a citation in the main text but to 1 and 2 in the supplement reference list. Either always refer to references as numbered in the main text, or refer to a supplement reference list for all citations.

Response 26: Thank you for this comment. We have corrected this issue.

Comment 27: I am not sure about the rules for Nature Communications but in my experience other journals expect the full DOI link to be provided for each reference. Reference 32: Cartigny et al. is (2013)

Response 27: Thank you for this comment. We have made sure our references are following the correct journal guidelines.

Overview

This is the response to reviewers for Manuscript NCOMMS-25-43051-B.

All line references in the following document correspond to the version of the manuscript called "*RevisedManuscript_NCOMMS-25-43051-B*" with tracked changes enabled (Simple Markup view in Microsoft Word).

We sincerely appreciate and thank the reviewers for the evaluation of our manuscript and their detailed and constructive comments. These suggestions have contributed to improving the quality, clarity, and precision of this manuscript.

Major manuscript changes

(1) Extended discussion of fallout deposit structure, location, and origin within the 2022 Hunga eruption

(2) Revised the discussion, specifically the section "*Eruption records in underwater density currents",* to improve the clarity of our conclusions and broader implications regarding the Hunga 2022 eruption deposits as a diagnostic tool of explosive shallow submarine explosive eruptions.

Editorial Changes

Abstract

- Minor changes to sentence structure.

Introduction

- Minor changes to sentence structure.

Results & Discussions

Complex flow regimes revealed from seafloor deposits

- Minor changes to sentence structure.
- Renamed Unit 1a-c to Facies 1a-c.
- Extended description of Facies 1c.
- Transferred a modified description of Unit 2 from Supplementary Materials to manuscript.
- Addition of discussion regarding the origin of Facies 1c (fallout material).

Spatial evolution of underwater density currents

- Minor changes to sentence structure.
- Renamed Unit 1a-c to Facies 1a-c.

Rapid entry of voluminous material fuels long-runout currents

- Minor changes to sentence structure.
- Renamed Unit 1a-c to Facies 1a-c.
- Extended discussion of the factors that influence the runout of turbidity currents to include grainsize.

- Extended discussion on the erosivity of the density currents to specifically identify the evidence for the erosion and entrainment of up to 3.5 km^3 of sediment.
- Extended discussion on fallout deposit material appearance within the eruption deposit.

Eruption records in underwater density current deposits

This section was substantially revised and extended. Please see Response 03 to Reviewer 1 for a detailed outline of manuscript changes. To summarise:

- Significantly modified this section to highlight that these deposits are specifically characteristic of large explosive shallow-submarine eruptions that can be easily distinguished from smaller discrete volcanism. However, distinction from volcanic activity of a similar size and scale requires more detailed comparisons using multiple deposit attributes but is possible.

Conclusions

- No changes made.

Methods

- No changes made.

Figures and Tables

Figure 1 – *Seafloor impact of underwater volcaniclastic density currents triggered during the January 15th, 2022, Hunga volcano eruption*

- Unit 1a-c renamed to Facies 1a-c in figure legend.
- Minor changes to legend captions.

Figure 2 – *Submarine volcaniclastic density current deposits*

- Unit 1a-c renamed to Facies 1a-c in figure legend

Figure 3 – *Grainsize analysis of density current deposits along the SW transect*

- Replaced the core images with brightened versions to more clearly illustrate deposit architecture.
- Addition of blue arrows to annotate recurring black sand layers and the base of fining-upward sequences.

Figure 4 – *Model of submarine volcaniclastic density current structure, behaviour, and evolution during the January 15th, 2022, Hunga volcano eruption*

- No changes made.

Figure 5 – *Schematics illustrating the formation of underwater volcaniclastic density currents at different volcanic settings*

- No changes made.

Table 1

- No changes made.

References

Six new references were added to this revised manuscript, and one was removed.

Here we list the new references and briefly identify the rationale for their addition. The number of each reference is identified by the bracketed number at the beginning of the reference e.g., (12):

Seismic record of the eruption and pulsatory T-wave signature:

- (24). Simon, J. D. et al. Hydroacoustic Observations of the 15 January 2022 Hunga Tonga-Hunga Ha'apai Eruption: The Role of Bathymetry Along the Path. JGR Solid Earth 131, e2025JB032996 (2026).

Fallout record, structure, and origin:

- (47). Baker, M. L. et al. Seabed Seismographs Reveal Duration and Structure of Longest Runout Sediment Flows on Earth. Geophysical Research Letters 51, e2024GL111078 (2024).
- (48). Barone, B., Letelier, R. M., Rubin, K. H. & Karl, D. M. Satellite Detection of a Massive Phytoplankton Bloom Following the 2022 Submarine Eruption of the Hunga Tonga-Hunga Haʻapai Volcano. Geophysical Research Letters 49, e2022GL099293 (2022).

Tsunami onset and inundation timings to identify timing of density current formation

- (57). Borrero, J. C. et al. Tsunami Runup and Inundation in Tonga from the January 2022 Eruption of Hunga Volcano. Pure Appl. Geophys. 180, 1–22 (2023).
- (58). Purkis, S. J. et al. The 2022 Hunga-Tonga megatsunami: Near-field simulation of a once-in-a-century event. Science Advances 9, 1–15 (2023).

The role of grainsize in density current runout:

- (65). Salaheldin, T. M., Imran, J., Chaudhry, M. H. & Reed, C. Role of fine-grained sediment in turbidity current flow dynamics and resulting deposits. Marine Geology 171, 21–28 (2001).

One reference was removed due to their being no available version of the manuscript:

- Cronin, S. J. et al. Submarine caldera subsidence creates extreme tsunami hazard. in pre-print (2025)

Supplementary Materials

<u>Editorial Changes</u>

- Removal of description of Unit 2 (pre-eruption seafloor material)

<u>Supplementary Figure 1</u> – *2022 Hunga eruption deposits*

- Minor legend text changes.
- Unit 1a-c renamed to Facies 1a-c in figure legend.

Supplementary Figure 2 – *Seabed images along the Southwest transect following the January 15th, 2022, Hunga volcano eruption*

- No changes made.

Supplementary Figure 3 – *Scanning electron microscope (SEM) images of material within the 2022 Hunga volcano eruption deposits from sediment core SW-80*

- No changes made.

Supplementary Figure 4 – *Volcanic glass composition of the 2022 Hunga submarine eruption deposits*

- No changes made.

Supplementary Figure 5 – *Geochemical Signature of the 2022 Hunga submarine eruption deposits*

- Minor legend text changes.
- Unit 1a-c renamed to Facies 1a-c in figure legend.

Supplementary Table 1 – *Summary of sediment cores collected during the TESMaP TAN2206 Research Cruise*

- No changes made.

Supplementary Table 2 – *Componentry of Sediment Core SW-64*

- No changes made.

Point-to-point Responses

All line references correspond to the updated version of the manuscript (Tracked Changes simple markup)

Reviewer #1

Comment 01: This is a resubmitted manuscript on pyroclastic density currents (PDCs) from the 2022 Hunga Tonga–Hunga Ha'apai (HTHH) eruption. I served as a referee on the previous version. The authors have revised the manuscript substantially in response to comments from both reviewers and have provided a point-by-point rebuttal. The new title more appropriately reflects the content.

My comments fall into two general categories:

(**a**) those concerning the technical details and conclusions of the study, for the authors, and

(**b**) those regarding the fit of the topic and manuscript, as currently constructed, for this particular journal, for the editors.

Response 01: Thank you very much for your review and valuable comments. In the accompanying updated manuscript, we have considered your concerns and suggested revisions and have made changes that aim to better clarify and focus our study conclusions.

Comment 02: Technical Assessment: Overall, the authors have significantly improved the manuscript. The eruption is now better contextualized, and the description of the deposits, including their distribution, lithology, and componentry, is clearer and more detailed. As a specialist, I found these sections particularly valuable. However, I am less certain that this level of detail maintains a smooth narrative flow for a more generalist readership and better be found in a supplement.

Response 02: Thank you for this comment. We acknowledge that the manuscript contains detailed sedimentological descriptions and discussions. However, the eruption deposit structure and architecture are the central findings of this paper, and we believe the level of detail provided is necessary to support and clarify our broader discussions (processes and diagnostic criteria). We do not deem it to supplementary information, instead it provides the requisite contextual information that support our conclusions; hence, its inclusion in the body text. Additional details were added in response to the first round of reviews, including the suggestion of moving deposit descriptions from the supplementary into the main manuscript.

Comment 03: The manuscript's primary conclusion, that voluminous PDC deposits are present at HTHH, is convincingly documented. The addition of the table summarizing similar events elsewhere is helpful. While not comprehensive, it adequately demonstrates that long-runout PDCs are not unique to this eruption and have been recognized at other submarine or mixed submarine–subaerial systems. That said, I am not entirely persuaded by the implication that long-runout PDCs are a characteristic or diagnostic feature of energetic submarine fragmental eruptions in general. The evidence here supports the presence of such deposits at HTHH, but the broader generalization remains somewhat speculative.

Response 03: Thank you for this comment. We agree with the reviewers that long-runout subaqueous volcaniclastic density currents are not diagnostic of all energetic submarine fragmental eruptions as evidenced by many of the case-studies highlighted in Table 1. Instead, we suggest that these density currents are specifically diagnostic of large explosive shallow-submarine eruptions.

This study is part of a growing body of case studies that, on the basis of deposit sampling, recognises voluminous and far-reaching submarine deposits and attributes them to underwater density currents triggered during large explosive marine eruptions:

- Mandeville et al., (1996). Bulletin of Volcanology 57, 512-529
- Schindlbeck et al., (2013). Marine Geology 343, 115-124
- Seabrook et al., (2023), Nature Communications 14, 7881
- Clare and Yeo et al., (2023). Science 38, 1085-1092
- Druitt et al., (2024). Commun Earth Environ 5, 24
- Metcalfe et al., (2024) Science Advances
- Cas et al., (2024). In Volcanology 891-981 (Springer International Publishing, Cham) doi: 0.1007/978-3-319-66613-6_11.

These mostly very recent studies focused on the recognition/interpretation of large and voluminous density currents demonstrate that this is a nascent area of research and are providing new insights into the wider understanding of explosive marine volcanic behaviour. Many of these studies, which focus on historical eruptions (Krakatau, Santorini, Kos etc.), are limited to deposit analysis, and lack real-time documentation and in-situ measurements to provide the categorical link between eruption and deposit. This is not the case for the January 15th, 2022, eruption of Hunga volcano, which was captured by satellite imaging, seismic monitoring stations, and in-situ photography, alongside comprehensive seafloor sampling (sediment cores) and pre- and post-eruption bathymetric surveys. Direct observations coupled with comprehensive sedimentological eruption records make Hunga a uniquely well-constrained case-study for characterizing and diagnosing these density current deposits, building upon earlier deposit-based studies. Currently, diagnostic criteria for identification of these far-reaching and voluminous density current deposits within the rock record have not yet been comprehensively outlined. Consequently, these density currents remain a hazard to maritime communities and infrastructure, especially with the proliferation of subsea telecommunication cables across oceans globally. We believe this emphasizes the importance of Hunga as case study, and this manuscript in outlining and distinguishing density current deposit characteristics.

To address the reviewers concerns regarding our broader conclusions, we have modified the section "*Eruption records in underwater density current deposits*" to highlight that these deposits are characteristic of large, high mass-flux, high mass-eruption rate, explosive, and voluminous eruptions, and can be distinguished from small discrete volcanic activity:

- **Lines 280–288 - "***Submarine deposits are often the only record of marine eruptions, their triggers, and other associated volcanic processes. The characterization of the 2022 Hunga Volcano eruption deposits, combined with repeat seafloor bathymetry and seismic records, offers the most comprehensive record of exceptionally rapid, syn-eruptive, pulsed, and multidirectional, delivery of volcaniclastic material during a large explosive (VEI 6) shallow-submarine eruption to date. As such, the spatial distribution and nature of such deposits can act as a guide for interpreting past comparable processes from stratigraphic records at other volcanic sites. This is particularly*

pertinent as there is an increasing body of work identifying voluminous and far-reaching volcaniclastic deposits and attributing them to density currents generated by large, energetic, and fragmental marine eruptions."

- **Lines 299-308 – "***Deposits of water-entering PDCs are typically unidirectional when parent flows are triggered by smaller, localized, events including dome collapses[3] and crumbling lava flow fronts[78]. In contrast, larger and more explosive eruptions can trigger radial dispersions of water-entering PDCs and ensuing underwater currents (e.g., following column collapse during the VEI 6 1883 Krakatau eruption)[76,79]. For minor, low-explosivity, volcanic activity that triggers discrete shoreline-crossing PDCs (e.g., Soufriere Hills Volcano dome collapses or recent activity at Stromboli), the subsequent unidirectional underwater currents have shorter runouts than identified at Hunga (Table 1). For larger eruptions (of similar VEI or greater to Hunga) density currents have been inferred to travel equivalent (or greater) distances, albeit with primarily unidirectional runouts except in the case of relatively small ocean island volcanoes[55,80]."*

- **Lines 308-315 – "***Near-shore deposition and only minor subaerial erosion associated with individual water-entering PDCs from small and low-explosivity eruptions differs greatly from the proximal flank erosion at Hunga (Figure 1). Distally, the Hunga 2022 deposits record stacked fining-upward sequences distinct from the single-layered, typically normally-graded and massive deposits recording infrequent planar and ripple laminations observed in deposits sampled offshore Montserrat[10,76]. This differing deposit structure likely reflects the contrast between continuous, pulsed, and multidirectional flows sustained by high eruption rates from a shallow vent (e.g. Hunga), as opposed to a discrete PDC generated during small-scale volcanic activity (e.g. Montserrat)."*

- **Lines 366-382 – "***The 2022 Hunga submarine eruption deposits document density currents generated during a large (VEI 5-6), explosive shallow submarine eruption characterized by high mass eruption rates (MER) and large erupted volumes (>6.5 km^3). A clear distinction exists between these deposits and deposits associated with density currents triggered by small-volume and low mass-flux volcanic activity e.g., shoreline-crossing PDCs associated with dome collapses, minor flanks collapses, surtseyan eruptions, or low-explosivity deep-submarine eruptions (Table 1), recognized by deposit extent and dispersion. For volcanic activity of comparable size and mobilized volumes to the January 15th eruption, density current runouts, dispersal, and deposit structures are far more analogous, regardless of current initiation mechanism (large volume mass wasting events, explosive submarine eruptions, and shoreline crossing PDCs; Table 1). Despite some shared characteristics, we conclude that deposits of density currents triggered by the rapid and sustained supply of volcaniclastic material directly onto the submerged flanks of the volcano during a large explosive shallow-submarine eruption, can be reliably distinguished from other density current initiation mechanisms. This is on the basis of combined multidirectional dispersion, extensive runout, stacked fining-upward sequences (following unsteady and pulsed delivery of material), and the proximal erosive scours and bedforms that sculpt the flanks of the volcano (trains of crescentic scours and incised channels)."*

Comment 04: The Missing Context of Fall Deposits: One important aspect remains insufficiently addressed, despite being raised in my previous review: the role, volume, stratigraphic significance, and broader implications of fall deposits in this eruption. Their omission in the prior form of the manuscript, titled "Title: The seafloor record of explosive submarine volcanic eruptions" was inexplicable, and yet even with the narrower retitled focus, I am still at a loss as to why they are not discussed.

In their response to reviewers (specifically comment #4), the authors cite my prior comment, but do not address its substance. This is a missed opportunity for contextualizing the PDCs within the full eruptive sequence.

The stratigraphic columns in the MS clearly show that Unit 1b (PDC deposits) constitutes the majority of the volume, but Unit 1c (fall deposits) are also substantial (approximately ~20% by my visual estimate). This raises several key questions:

- What is the volumetric and spatial relationship between fall deposits and PDC deposits
- Is this ratio diagnostic of the eruption style or specific to this geologic setting?
- Do fall deposits strictly postdate the PDCs, or might they be cryptically interbedded?
- How do thinning, fining, and dispersion patterns in the fall deposits compare with expectations given the dynamics of plume dispersal, wind, and ocean currents?

Although not the central focus of the manuscript, the fall deposits remain an essential component of the eruptive record and tephra-dispersal system, and of other submarine volcanoes generally. Their omission here risks implying that long-runout PDCs are the sole mechanism of regional tephra transport.

Relevant published work underscores Fall deposit significance. Barone et al. (2022) – a paper I referred the authors to previously - document satellite-observed ocean-surface changes (opacity, color, temperature, chlorophyll, plankton count) attributable to ashfall from the eruption, implying a minimum regional ash thickness of ~20–197 mm. Kelly et al. (2024) estimate a minimum fall-deposit volume of ~1.8 km^3 (out of >6.3 km^3 total erupted volume). This proportion is consistent with the stratigraphic estimate I made based on data in the current manuscript.

Given this, the manuscript could at least briefly:

- Describe the fall deposits and cite the relevant studies
- Note their position(s) in the eruptive sequence (Barone et al. report two separate fall events),
- Discuss the partitioning of tephra between PDCs and fallout, and
- Comment on whether this partitioning is typical of submarine, subaerial, or hybrid eruptions.
- Such additions would provide a more complete picture of the eruption and better situate the PDC deposits within the broader volcanological context. They could also highlight useful avenues for future work.

References:

- Barone et al. (2022), Geophysical Research Letters, 49(17), e2022GL099293.
- Kelly et al. (2024), Bulletin of Volcanology, 86:59.

Response 04: Many thanks for this comment. Whilst we agree that fallout is an important aspect of any eruption record it is important to clarify that the fallout record within the sediment cores collected offshore Hunga is incomplete. The estimated arrival times for settling plume material to fully reach the

seafloor is up to six months (Chaknova et al., 2025) and suspended volcaniclastic material (of Hunga origin) within the water column was documented at the time of sediment core recovery (~ 3 months after the eruption; Seabrook et al., 2023). As such, our ability to conclusively address the key questions – volumetric and spatial relationships between fallout and current deposits, determine if this relationship is diagnostic of this eruption style, and the role of atmospheric and ocean influences – is restricted. Hence, our limited discussion of fallout material within this manuscript.

As of writing this response, new sediment cores were collected offshore Hunga in September 2025 at the same sample locations collected shortly after the eruption in 2022. Future research will focus explicitly on identifying and outlining the true thickness(es) and extent of the submarine fall deposits and will hopefully address the key questions outlined.

Despite an incomplete fallout record in the sediment cores, we agree that our description and discussion of the available fall-deposit should be expanded in the current manuscript. As such, we have attempted to address your suggested manuscript additions where possible:

(1) We have extended our description of the fall deposits within the sediment cores, and how they are distinguished from the density current deposit below:

- **Lines 98-101 – "***Unit 1 is consistently overlain by Facies 1c, a thin, centimetre (< 4 cm), layer of poorly sorted clay, distinguished from material below by a subtle colour change with an absence of internal structures (e.g., the planar laminations of Facies 1b).*"

(2) Specifically identified where the fall-deposits are located within the eruption deposit:

- **Lines 101-102 –** *"Facies 1c is not observed/recognised at any other stratigraphic location within the 2022 eruption deposit."*

(3) Added a substantially extended discussion on the possible origins of the fall-deposits:

- **Lines 146-162** – *"Moving up-deposit from Facies 1b to 1c, the change in internal deposit structure indicates a shift in depositional mechanism, which has been previously identified as a transition from emplacement by turbulent density currents to eruption plume fallout[45]. Evidence for plume fallout mantling the density current deposits is threefold: all sediment core locations lie within the 2022 eruption plume radius; Facies 1c grainsize and thicknesses (< 4 cm) match estimated plume material grainsize (<100 μm) and fallout deposit thicknesses (1-6 cm)[46]; and modelled current velocities indicate density currents reached core sites[13,15] before plume fallout settled on the seafloor[45]. However, the ungraded veneer of Facies 1c can also be attributed to the uppermost structural division of the turbidite sequence – fallout of fine material suspended in the water-column by a turbulent density current that can remain in suspension for days-to-months[36,47]. This is supported by the occurrence of lithic and biogenic material within facies 1c that is unlikely to be sourced from plume fallout. Regardless of source, calculated settling rates[45] and continued settling of suspended particulate documented by video and water-column sampling three months after the eruption[15], restricts fallout to the top of the deposit. Facies 1c is likely a combination of both plume fallout and settling of material suspended by density currents, that is challenging to conclusively differentiate between them. Despite fallout into the water surrounding Hunga documented during the precursory volcanism at Hunga there is no evidence of a fallout layer between Unit 1 and Unit 2[48]."*

(4) Cited the suggested relevant studies:

- Barone et al. (2022), Geophysical Research Letters, 49(17), e2022GL099293.
- Kelly et al. (2024), Bulletin of Volcanology, 86:59.

(5) Extended the section "*Rapid entry of voluminous material fuels long-runout currents"* to discuss the position of fallout deposits with the eruption deposit and contextualising them within the eruption. Specifically, we have focused on the absence of ash fallout interspersed through the deposit or at the base of the eruptive sequence (given the observation of fallout prior to the paroxysmal January 15th eruption).

- **Lines 267-270**– *"Given current erosivity, at least proximally, any erupted material subaqueously deposited shortly before the January 15th eruption e.g., ash-fallout on January 13th, was likely eroded, explaining the absence of a preserved record of precursory eruptions between Unit 1 and Unit 2."*

- **Lines 274-276 – "***The majority of the 6.5 km^3 of pyroclastic material was deposited onto the seafloor during the ~60-minute window of maximum MER, explaining the lack of fine-grained mud capping between black sands. Given this timeframe, it seems unlikely that plume-fallout layers are preserved within the main body of the 2022 eruption deposit[45]."*

Reviewer #3

Comment 01: My (and other reviewers ‘) comments appear to have been adequately considered in the revised manuscript. Particularly the discussion section about comparison between various modes of volcaniclastic turbidity current formation and their deposits has been greatly improved. I do, however, still have some minor comments.

Response 01: Thank you for your review and highly valuable comments.

Comment 02: Line 249: another important factor for long runout is fine grain size, i.e. intense fragmentation in/near vent.

Response 02: Thank you for highlighting this missing information. We agree that this is an important factor to include as the ash erupted on January 15th, 2022, was very fine and likely contributed to the extensive runout of the density currents. As such, we have included this information in the manuscript:

- **Lines 252-256 –** *“Generating long-runout turbidity currents requires preservation of excess density which can be achieved through a combination of initially high current speeds[63], large volumes of mobilized material[64], fine sediment grainsize[65], erosion leading to flow bulking (ignition)[66], or a semi-continuous supply of material[67], all of which might have transpired during, intense fragmentation, low-eruption column collapses, and submarine jetting/fountaining, particularly during a period of sustained column growth.”*

Comment 03: A brief description of the pre-eruption unit 2 sediments should be included on page 3. This becomes relevant when entrainment of such sediment is invoked as a means to extend the runout distance of the currents (lines 256 ff).

Response 03: Thank you for this comment. We agree, and have moved our description of Unit 2 pre-eruption seafloor sediments from the Supplementary Material to the main manuscript:

- **Lines 122-129–** “*Beneath Unit 1 is Unit 2, a compacted, clay-rich but volcanic sourced deposit containing abundant volcanic glass shards. The contact between units is characterized by a pronounced grainsize break and visually distinct colour change. Unit 2 is distinguished from the 2022 eruption deposits by contrasting geochemical signatures (low Fe/Ti, high K/Ti, and low Cl), abundant biogenic material (e.g., shell fragments and foraminifera), alongside intense weathering, oxidation, and bioturbation (Figure 1 and Figure 2). Instead, these sediments comprised the seafloor prior to the 2022 eruption with a combined source of pelagic fallout and previous volcanic eruptions (from either Hunga or surrounding Kermadec Arc volcanoes).”*

Comment 04: The volume of ~3.5 km3 of entrained sediment (line 257) needs to be explained and a reference added (presumably no. 15 ??).

Response 04: Thank you for this comment. We have provided additional information and references to explain how the 3.5 km^3 of entrained material was calculated and the role of the density currents:

- **Lines 261-263** – "*Bulking through flank erosion (entraining an additional ~3.5 km*^{3} *as identified by pre- and post-eruption bathymetry*[15] *and erosive sedimentological bedforms*[13]*) would have further enhanced the concentration of these flows, also promoting formation and maintenance of a dense near-bed layer*[50,68,69]*.*"

Comment 05: Can this sediment be distinguished from the newly erupted material, and if so, was it recognized in the cores?

Response 05: This is an interesting and very pertinent question. Can entrained sediment be distinguished from juvenile material within the 2022 Hunga eruption deposits? – yes, just not in this manuscript.

Identification of entrained sediments within the Hunga eruption-related density current deposits is complex as a significant proportion of entrained material is volcanic glass of similar texture and geochemical composition to juvenile material (based on SEM and EDS analysis). There are indicators of entrainment identified in this manuscript, specifically, the presence of biogenic material up-through the eruption deposits that is unlikely to have been sourced from anywhere other than eroded seafloor (see our response above regarding ash fall deposits). To discern between volcanological material (glass, lithics, crystal etc.) requires a much deeper dive into component geochemistry (major and minor elements) and textures.

Ongoing research focuses on volcanic geochemistry (EPMA & LA-ICP-MS) and Fourier transform infrared spectrometry (FTIR) analysis to distinguish entrained vs freshly erupted material with the submarine Hunga eruption deposits and will be published in a subsequent manuscript.

Comment 06: Reference 24 is used several times in the context of seismic signals supporting the present model of initiation and motion of the turbidity currents. However, this information is not accessible (in pre-print but no information where it might eventually be found). Is there a way to briefly show relevant seismic evidence in this paper?

Response 06: Thank you for this comment. Unfortunately, as of writing this response, the reference "*Cronin, S. J. et al. Submarine caldera subsidence creates extreme tsunami hazard. in pre-print (2025)*", is still under review with no online version available. We requested permission from the authors to include the relevant information, either as a modified figure or as data within our supplementary material. However, permission was not granted by the manuscript authors. As such, we have removed the reference, and the manuscript has been revised accordingly. Instead of this reference we have modified our discussion to focus on other studies that outline the timings and semi-continuous nature of the density currents triggered during the eruption on the 15th, allowing us to arrive at the same conclusions. Specifically, we highlight eruption plume analyses, tsunami onset timings, and other seismic studies:

- **Lines 205-223 – "***Despite the evacuation of ~6.5 km*^{3} *of magma (Dense Rock Equivalent, DRE) during the eruption, the 58 km high eruption column was tephra poor (<0.1-0.2 km*^{3} *of erupted volume)*[22,29,30]*. Instead, the bulk of erupted material entered the ocean*[22,46]*. We therefore attribute underwater volcaniclastic density current formation to syn-eruptive and vertical delivery of large*

volumes of erupted material into the ocean via plume collapse[13,15] and submarine or low-level subaerial fountaining and jetting leading to lateral density current dispersal (Figure 4)[22,56]. Direct observations of low column-collapses and tsunami onset timings indicate the continued delivery/supply of pyroclastic material into the ocean and the semi-continuous formation of underwater currents during the period of highest MER (timeframe of most rapid column growth between ~04:00-05:10) shortly after eruption onset[13,57,58], supported by unsteady and pulsatory seismic energy records of the eruption[24]. Semi-continuous generation of underwater volcaniclastic density currents, over a short time period supports our interpretation that the repeated fining upward nature of the 2022 deposits is indicative of deposition from a series of quasi-steady pulsed currents. The close timing between the first major explosion (and rapid column growth), visible plume collapses, and cable damage, support low-column collapses as the primary mechanism of initial current formation[13]. However, the precise initiation mechanism(s) (i.e. column-collapse, fountaining, jetting, or combinations thereof typically associated with a large explosive eruption), are all capable of, and likely all contribute to, rapidly and vertically delivering large volumes of dense material into the ocean directly onto submerged volcano flanks, providing an efficient mechanism to trigger pulses of density currents in multiple directions[13].”

- **Lines 272-274 – “***Eruption plume analyses indicate maximum MER between ~04:10-05:10, before declining [19,25,30]. The majority of the 6.5 km^3 of pyroclastic material was deposited onto the seafloor during the ~60-minute window of maximum MER, explaining the lack of fine-grained mud capping between black sands.”*

Comment 07: This may be important additional evidence because I still find the identification of the “stacked fining-up sequences” a bit weak. Not that I doubt the unsteady nature of material delivery during the eruption but how this is deduced from the deposits is not so easy to follow while this is used as a major distinguishing criterion in the last discussion section. In distal cores it is not seen because of the overall finer grain size, in the most proximal core 64 the laminograph (Fig. 2) shows change from upper plane bed to ripple structure while grain size indicates possible three cycles (Fig. 3). In core 80 there is visual evidence of repeated darker layers (Fig. 2) but no real grain-size evidence (Fig. 3). So, I think it would be worthwhile to more clearly present the evidence for (at least 6) stacked cycles.

Response 07: Thank you for this comment. We agree that the grain-size data presented in Figure 3, particularly for the distal cores (e.g., SW-100), is insufficient in identifying stacked sequences alone, and must be evaluated alongside other criteria. This is largely in part due to grain-size data being collected across centimetre window intervals down core rather than at specific layers which can be < 1 cm thick (recurring black sand layers). Averaging grain-size over the sampled window can lead to subtle variations in grain-size being missed. To address this, we use visual changes across the deposit in combination with grainsize data to identify vertical grain-size variations, and subsequently, stacked sequences:

- **Lines 96-97 – “***Different layers are distinguished by distinct colour (Figure 2) and grainsize changes (Figure 3)”*

However, we acknowledge that our identification of these recurring black sand layers and stacked sequences would benefit from greater visual clarity. We have therefore modified Figure 3 with a series

of blue arrows indicating the location of recurring black-sand layers and the base of a fining upward sequence.

Comment 08: Colour coding in the XRF profiles Fig. 2: Unit 1a is used here as Facies 1a, a unit has a fixed stratigraphic position while a facies can occur repeatedly. Should not be confused. Fig. 2 has nice light core photographs with well-visible structures while those in Fig. 3 are too dark! Lighten up!

Response 08: Thank you for this comment. We have renamed Unit 1a-c, to Facies 1a-c across the manuscript and all relevant figures. Additionally, Figure 3 has been modified, with brightened core images replacing the unedited images to better illustrate the deposit structure and architecture.

Comment 08: Lines 295 ff: distinguishing "*continuous, pulsed*" (Hunga) from "*single large volume PDC*" appears a bit artificial. This can be made for the comparison with some deposits but not with others. Where large PDCs entered the ocean, ignimbrite on land mostly shows several flow units, i.e. "pulse-like" emplacement rather than "at once". Consequently, submarine turbidites from such events can well contain fining-up cycles (e.g., Schindlbeck et al. 2013, Kutterolf et al. 2014, Freundt & Schmincke 1998). One aspect that is not discussed here is that submarine tuffs from large-magnitude PDC eruptions can show vertical density grading of clasts.

Response 08: Thank you for this comment. We recognise the concerns of both reviewers in the section "*Eruption records in underwater density current deposits*" around terminology, comparisons, and broader conclusions. To address these concerns, we have reframed and refined this section to explicitly clarify our meaning (please see Response 03 to reviewer 1).

To briefly summarise the contents of the edited section:

- The deposits characterized in this manuscript are specifically diagnostic of voluminous density currents triggered by large explosive shallow-submarine eruptions, of which there is a growing body of case studies, that can be easily distinguished from smaller, low-explosivity, volcanic events.
- Comparisons with density current deposits from volcanic activity of a similar (or greater) scale to the Hunga 2022 eruption (e.g., mobilised sediment volume, explosivity etc) requires a more detailed comparison, but differences can be identified.

We agree that density grading is a key feature of submarine eruption deposits. We have not included a detailed discussion of this parameter in the present manuscript as doing so requires comprehensive componentry and density analysis across all sediment cores, which at the time of writing this manuscript is incomplete. However, we do include componentry data for sediment core SW-64 in the Supplementary Material (Supplementary Table 2), which provides preliminary insights into density sorting within the Hunga deposits (e.g., variation in proportion of pumice and other components). Detailed componentry analysis across all cores is currently being undertaken, in tandem with geochemical analysis, which will form the basis of entire research paper exploring whether compositional and density variations throughout the density current deposits (vertically and laterally) relates to magmatic or sedimentological processes (or both).

Comment 09: Line 298: Miocene Gran Canaria had radial distribution of PDCs into the ocean, and this is not a particularly small island. The more important factor would be volume flux versus depth of topography, I guess.

Response 09: Thank you for this comment. Please see above response.

Overview

This is the response to reviewers for Manuscript NCOMMS-25-43051C

All line references in the following document correspond to the version of the manuscript called "*RevisedManuscript_NCOMMS-25-43051C*" with tracked changes enabled (Simple Markup view in Microsoft Word).

We sincerely appreciate and thank the reviewers for the evaluation of our manuscript and their detailed and constructive comments. These suggestions have contributed to improving the quality, clarity, and precision of this manuscript.

Major Manuscript Changes

- No major changes

Editorial Changes

Authorship

- Corresponding author email added beneath author list

Title

- Title has been changed from "*Voluminous, far-reaching, and multidirectional volcaniclastic density currents are key records of explosive marine eruptions*" to *"Far-reaching volcaniclastic density currents as evidence of explosive marine eruptions"*
- This change has been implemented to remove exaggerating language (e.g., "*Key*") and to make the title briefer

Abstract

- Minor changes to sentence structure.
- Addition of VEI 6 to indicate the

Introduction

- No changes.

Results & Discussions

The 2022 eruption of Hunga volcano

- No changes.

Complex flow regimes revealed from seafloor deposits

- No changes.

Spatial evolution of underwater density currents

- No changes.

Rapid entry of voluminous material fuels long-runout currents

- No changes.

<u>Eruption records in underwater density current deposits</u>

- Single sentence added discussion the transferability of analysis on the 2022 Hunga eruption deposits to smaller eruptions:

    - **Lines 372-373: "***However, due to the size of the January 15th eruption, not all observations or diagnostic criteria from this event will necessarily be transferrable to smaller eruptions."*

Methods

- Additional details added to the sections "*Bathymetric Surveys"* and "*Componentry"* such that the methods outlined can be reproduced without reference to reference to published papers, specifically the creating of the elevation difference map and the specific Scanning Electron Microscopes used for componentry analysis:

    - **Lines 398-411: "***Multibeam bathymetric data detailing the post-eruption topography of Hunga Volcano, and the surrounding seafloor (Figure 1) was collected using a Kongsberg Simrad EM302 multibeam echosounder on the RV Tangaroa during the TAN2206 Cruise in April and May 2022[14,15,102]. Seafloor elevation changes relating to the January 15th, 2022, eruption of Hunga volcano were determined by comparing this post-eruption bathymetric data with a digital elevation map (DEM) comprised of pre-eruption bathymetric data acquired both remotely and on two separate research expeditions[13,15]: (1) 2017 satellite-derived bathymetry[103], (2) the May 2016 FK160407 Expedition on the RV Falkor using a Kongsberd Simrad EM302[104], and (3) November 2015 small boat survey using a WASSP multibeam echosounder[16]. The vertical resolution of these data is typically 1% of the water depth. The pre-and post-bathymetric data is available online at Zenodo[102]. Bathymetric data from the different surveys were horizontally grided to 50x50 m. These pre- and post-eruption DEMs were imported into ArcPro GIS where the post-eruption surface was subtracted from the pre-eruption surface to create an elevation difference raster of the seafloor around Hunga that visualises regions of erosion (negative values)and deposition (positive values)[13,15]."*

    - **Lines 536-543: "***Analyses were done via particle counting to a minimum of 300 grains using the TESCAN CLARA High-Resolution Field-Emission Gun Scanning Electron Microscope (FEG-SEM) and Leo 1450VP Scanning Electron Microscope at the University of Auckland and NOC, respectively. Componentry was based on macroscopic external characteristics (morphology, vesicularity and crystallinity) and geochemical composition. Geochemical composition was determined from energy-dispersive X-ray spectroscopy (EDS), using the Oxford instruments 65 mm^2 active area energy dispersive spectrometer and Oxford instruments X-act 10mm^2 area SEM-energy dispersive spectrometer at the University of Auckland and NOC, respectively."*

- Additional referencing for the TESMaP data collection and data analysis:

- **Lines 397-399:** *"Multibeam bathymetric data detailing the post-eruption topography of Hunga Volcano, and the surrounding seafloor (Figure 1) was collected using a Kongsberg Simrad EM302 multibeam echosounder on the RV Tangaroa during the TAN2206 Cruise in April and May 2022[14,15,102]."*

- Identified the online data repositories storing that the Hunga pre- and post-eruption bathymetry, Tonga-Kermadec Arc topography, and South Pacific tectonic plate boundaries were sourced from:

  - **Lines 406-407: "***The pre-and post-bathymetric data is available online at Zenodo[102]."*

  - **Lines 411-416: "***As the South-West transect profile crosses areas of seafloor not covered by the 2022 bathymetric survey, we utilise the Global Multi-Resolution Topography (GMRT; dataset Version 4.2, released May 2024[105]), to illustrate topographic change across the transect (Figure 1). The location of Hunga volcano on the Tonga-Kermadec arc, and its relation to local plate tectonics, was illustrated in Figure 1a using General Bathymetric Chart of the Oceans (GEBCO) 2025 Grid[106] and the U.S. Geological Survey plate boundary data[107].*

Figures and Tables

Figure 1 – *Seafloor impact of underwater volcaniclastic density currents triggered during the January 15th, 2022, Hunga volcano eruption*

- Figure 1a – added references to the open access GEBCO (https://www.gebco.net/data-products/gridded-bathymetry-data) and USGS Plate Boundary (https://earthquake.usgs.gov/learn/plate-boundaries.kmz) datasets within the figure caption.

- Figure 1b-c – added reference to open access data (https://zenodo.org/records/745632) and Global Multi-Resolution Topography (GMRT; https://www.gmrt.org/about/#access) within the figure captions.

Figure 2 – *Submarine volcaniclastic density current deposits*

- No Changes.

Figure 3 – *Grainsize analysis of density current deposits along the SW transect*

- No Changes.

Figure 4 – *Model of submarine volcaniclastic density current structure, behaviour, and evolution during the January 15th, 2022, Hunga volcano eruption*

- No Changes.

Figure 5 – *Schematics illustrating the formation of underwater volcaniclastic density currents at different volcanic settings*

- Transferred the text from each image panel to figure caption.

- Modified figure caption to illustrate that these are original figures based on processes discussed in specific publications:
    - **Lines 924-925: "***Schematics are based on interpretations and processes discussed in Cas & Wright (1991)[1], White (2000)[2], and Freundt (2002)[6].*"

Table 1

- No changes made.

Data and Material Availability

- This section was extended to include the location of open-source data used in manuscript figures:

    - **Lines 549-554: "***Pre- and post-eruption bathymetric data of Hunga volcano and the surrounding seafloor can be accessed in Zenodo (https://zenodo.org/records/7456324)[102]. Global Multi-Resolution Topography (GMRT) data is open access (https://www.gmrt.org/about/#access)[105]. General Bathymetric Chart of the Oceans (GEBCO) gridded ocean land and terrain models is open access (gebco.net/data-products/gridded-bathymetry-data)[106]. U.S. Geological Survey (USGS) plate boundaries dataset is open access (https://earthquake.usgs.gov/learn/plate-boundaries.kmz)[107].*"

References

Three new references for open access datasets used in the manuscript figures were added:

- (102) Mackay, K. Digital Elevation Models of Hunga Volcano; pre- and post- 15 January 2022 eruption. https://doi.org/10.5281/zenodo.7456323 (2022) doi:10.5281/zenodo.7456323.

- (106) GEBCO Compilation Group. GEBCO 2025 Grid. https://doi.org/doi:10.5285/37c52e96-24ea-67ce-e063-7086abc05f29 (2025).

- (107) U.S. Geological Survey. Plate Boundaries. (2025).

Acknowledgements

- Minor changes made to relevant funding section, with the addition of *NE/X01519X/1* (IAY, MAC, JH).
- **Lines 859-862:** "*This work was supported by Natural Environmental Research Council grants NE/Z504038/1 (MAC and IAY), NE/X00239X/1 (MAC and IAY), NE/X003272/1 (IAY, MAC, JH), and NE/X003272/1(IAY, MAC, JH) and Jacob Nash acknowledges salary and research support from NE/S007415/1 (JAN).*"

Author Contributions

- Modified author initials after review and editing section.

Competing Interests

- No changes made.

Supplementary Materials

Editorial Changes

- No changes.

Supplementary Figure 1 – *2022 Hunga eruption deposits*

- No changes.

Supplementary Figure 2 – *Seabed images along the Southwest transect following the January 15th, 2022, Hunga volcano eruption*

- Figure 2a - added references to TESMaP TAN2206 Research Cruise, open access bathymetry data(https://zenodo.org/records/745632), and Global Multi-Resolution Topography (GMRT; https://www.gmrt.org/about/#access) within the figure caption. See *Supplementary References* section below or the Supplementary Material Document for more details on the references.

Supplementary Figure 3 – *Scanning electron microscope (SEM) images of material within the 2022 Hunga volcano eruption deposits from sediment core SW-80*

- No changes.

Supplementary Figure 4 – *Volcanic glass composition of the 2022 Hunga submarine eruption deposits*

- No changes.

Supplementary Figure 5 – *Geochemical Signature of the 2022 Hunga submarine eruption deposits*

- No changes.

Supplementary Table 1 – *Summary of sediment cores collected during the TESMaP TAN2206 Research Cruise*

- No changes.

Supplementary Table 2 – *Componentry of Sediment Core SW-64*

- No changes.

Supplementary References

- Added four references new references:

  - 1.Mackay, K. et al. Environmental Impacts of the 2022 Eruption of Hunga Tonga - Hunga Ha'apai: Voyage Report of Part 1 of the TesMAP Survey of the Region in April-May 2022 (TAN2206). (National Institute of Water & Atmospheric Research Ltd, Wellington, 2022).

  - 2.Mackay, K. Digital Elevation Models of Hunga Volcano; pre- and post- 15 January 2022 eruption. https://doi.org/10.5281/zenodo.7456323 (2022) doi:10.5281/zenodo.7456323.

  - 4.Clare, M. et al. Fast and destructive density currents created by ocean-entering volcanic eruptions. Science (New York, N.Y.) **381**, 1085–1092 (2023).

  - 5.Ryan, W. B. F. et al. Global Multi-Resolution Topography synthesis. Geochemistry, Geophysics, Geosystems **10**, 2008GC002332 (2009).

<u>Supplementary Datasets</u>

- Added minor information changes such as core distance from the volcano, water depth of samples, latitude and longitude of cores, to the datasets.

<u>Point-to-point Responses</u>

All line references correspond to the updated version of the manuscript (Tracked Changes simple markup)

<u>Reviewer #1</u>

Comment 01: I have had the opportunity to reread the revised manuscript and the authors' extensive documentation addressing the editorial changes in this version. The manuscript resolves all my previous concerns. The manuscript is now clearly written, methodologically sound, and strikes an appropriate balance between specialist detail and broader accessibility. As I said in prior reviews, the topic is inherently engaging. I feel that this is an important study, one that forms what will likely be a widely read contribution to the journal. I therefore recommend publication in its present form.

I also wish to note that the authors' discussion of fall deposits and the admixture of lithic fragments, including those of biogenic origin, together with the settling-velocity arguments, provides a compelling and well-supported explanation for why these materials were not a primary focus of the paper. I also accept the nuances they have added about sea-surface crossing versus entirely submarine PDCs

Upon reflection, my earlier comments about fall deposits may have been too influenced by my own observations from other volcanic systems. In particular, I believe there is an important distinction between fall deposits derived from eruption columns that breach the air–sea interface and those that do not. The latter may be significant at other volcanoes, especially proximal to the volcanic vent, but they do not appear to have been central to this specific eruption, especially at vent distal locales.

Response 01: Thank you very much for your review, comments, and support for the publication of this manuscript.

Comment 02: Yet, since most of the examples given are for large eruptions, I would be tempted in the body of the paper to recognize in a sentence that not all observations from this event will necessarily be transferable to smaller eruptions.,, and then perhaps even speculate that the proportion of PDC to Fall deposition may be nonlinear with mass discharge rate.

Response 02: Thank you for this comment. We agree that it is important to acknowledge the limits on observation and analysis transferability between the January 15th eruption at Hunga and smaller explosive shallow-submarine eruptions. As such we have added the following sentence:

- **Lines 373-374: "***However, due to the size of the January 15th eruption, not all observations or diagnostic criteria from this event will necessarily be transferrable to smaller eruptions."*

Comment 03: If I were to offer one other minor suggestion to further strengthen an already excellent write-up, it would be to emphasize in the abstract, perhaps even in the very first sentence, that this event represents the largest observed eruption on Earth this century. Its marine setting and exceptional level of documentation provide unprecedented clarity into the behaviour of such large-scale systems.

Response 03: Thank you for this comment. We agree that the scale of the 2022 Hunga eruption is significant.

We have modified the abstract to include reference to size of Hunga eruption:

- **Lines 17:** *"following the explosive, VEI 6, shallow-submarine eruption of Hunga Volcano in 2022"*

We do include reference to the eruption's explosivity relative to other eruptions:

- **Lines 63-64:** ***"****activity at Hunga Volcano rapidly escalated into the most explosive volcanic eruption of the 21st century[19,20]."*

<u>Reviewer #3</u>

Comment 01: My comments on the first revision have now been adequately addressed in the second revision, and I have nothing more to add.

Response 01: Thank you very much for your review, comments and support for the publication of this paper.
